# Antimicrobial resistance in paediatric *Streptococcus pneumoniae* isolates amid global implementation of pneumococcal conjugate vaccines: a systematic review and meta-regression analysis

**DOI:** 10.1016/S2666-5247(21)00064-1

**Published:** 2021-09

**Authors:** Kristin Andrejko, Buddhika Ratnasiri, William P Hausdorff, Ramanan Laxminarayan, Joseph A Lewnard

**Affiliations:** a Division of Epidemiology, School of Public Health, University of California, Berkeley, CA, USA; b Division of Infectious Diseases and Vaccinology, School of Public Health, University of California, Berkeley, CA, USA; c Center for Computational Biology, College of Engineering, University of California, Berkeley, CA, USA; d College of Letters and Science, University of California, Berkeley, CA, USA; e PATH, Washington, DC, USA; f Faculty of Medicine, Université Libre de Bruxelles, Brussels, Belgium; g Center for Disease Dynamics, Economics and Policy, New Delhi, India; h High Meadows Environmental Institute, Princeton University, Princeton, NJ, USA

## Abstract

**Background:**

Pneumococcal diseases are a leading cause of morbidity and mortality among children globally, and the burden of these diseases might be worsened by antimicrobial resistance. To understand the effect of pneumococcal conjugate vaccine (PCV) deployment on antimicrobial resistance in pneumococci, we assessed the susceptibility of paediatric pneumococcal isolates to various antimicrobial drugs before and after PCV implementation.

**Methods:**

We did a systematic review of studies reporting antimicrobial susceptibility profiles of paediatric pneumococcal isolates between 2000 and 2020 using PubMed and the Antimicrobial Testing Leadership and Surveillance database (ATLAS; Pfizer). Population-based studies of invasive pneumococcal disease or nasopharyngeal colonisation were eligible for inclusion. As primary outcome measures, we extracted the proportions of isolates that were non-susceptible or resistant to penicillin, macrolides, sulfamethoxazole–trimethoprim, third-generation cephalosporins, and tetracycline from each study. Where available, we also extracted data on pneumococcal serotypes. We estimated changes in the proportion of isolates with reduced susceptibility or resistance to each antibiotic class using random-effects meta-regression models, adjusting for study-level and region-level heterogeneity, as well as secular trends, invasive or colonising isolate source, and countries' per-capita gross domestic product.

**Findings:**

From 4910 studies screened for inclusion, we extracted data from 559 studies on 312 783 paediatric isolates. Susceptibility of isolates varied substantially across regions both before and after implementation of any PCV product. On average across all regions, we estimated significant absolute reductions in the proportions of pneumococci showing non-susceptibility to penicillin (11·5%, 95% CI 8·6–14·4), sulfamethoxazole–trimethoprim (9·7%, 4·3–15·2), and third-generation cephalosporins (7·5%, 3·1–11·9), over the 10 years after implementation of any PCV product, and absolute reductions in the proportions of pneumococci resistant to penicillin (7·3%, 5·3–9·4), sulfamethoxazole–trimethoprim (16·0%, 11·0–21·2), third-generation cephalosporins (4·5%, 0·3–8·7), macrolides (3·6%, 0·7–6·6) and tetracycline (2·0%, 0·3–3·7). We did not find evidence of changes in the proportion of isolates non-susceptible to macrolides or tetracycline after PCV implementation. Observed changes in penicillin non-susceptibility were driven, in part, by replacement of vaccine-targeted serotypes with non-vaccine serotypes that were less likely to be non-susceptible.

**Interpretation:**

Implementation of PCVs has reduced the proportion of circulating pneumococci resistant to first-line antibiotic treatments for pneumonia. This effect merits consideration in assessments of vaccine impact and investments in coverage improvements.

**Funding:**

Bill & Melinda Gates Foundation.

## Introduction

*Streptococcus pneumoniae* (pneumococcus) causes clinical conditions encompassing upper respiratory infections such as otitis media and sinusitis, non-bacteraemic pneumonia, and severe invasive diseases such as bacteraemic pneumonia, sepsis, and meningitis.^1^ Historically, pneumococcus has been the leading infectious cause of death among children younger than 5 years globally,^2^ and a prominent cause of antibiotic consumption.^3^, ^4^ Antimicrobial resistance in pneumococcus was first reported in 1963,^5^ and prevalence has since increased substantially, with more than 50% of pneumococcal isolates routinely showing diminished susceptibility to penicillin in particular regions.^6^

Pneumococcal conjugate vaccines (PCVs) target serotypes of *S pneumoniae* associated with the majority of both invasive and mucosal disease.^7^ Before vaccine implementation, serotypes included in the first-generation 7-valent conjugate vaccine (PCV7) were 12·5-times as likely as non-PCV7 serotypes to show diminished susceptibility to penicillin in the USA, and accounted for 79% of penicillin-non-susceptible invasive pneumococcal disease (IPD) cases,^8^ consistent with observations in other settings.^9^ Randomised controlled trials and post-licensure observational studies confirmed that PCV-conferred protection against these serotypes reduces the burden of resistant pneumococcal infections.^10^ However, replacement of vaccine-targeted serotypes by non-vaccine serotypes has been reported in nasopharyngeal carriage and invasive disease after PCV implementation.^11^ Concomitant expansions in non-susceptibility or resistance have been reported within non-vaccine serotypes in certain settings.^12^, ^13^, ^14^ Incomplete understanding of the net results of these changes among circulating pneumococci has forestalled assessments of the utility of PCVs in reducing the burden of antimicrobial resistance.^15^

**Research in context**

**Evidence before this study**

We searched for articles using PubMed, supplemented with data from the Antimicrobial Testing Leadership and Surveillance (ATLAS; Pfizer) for studies reporting antimicrobial susceptibility profiles of paediatric pneumococcal isolates between 2000 and 2020. A detailed summary of the search terms is included in the appendix (pp 3–8). The search string of our systematic review revealed no previous reviews synthesising data from multiple settings on the effect of pneumococcal conjugate vaccines (PCVs) on the distribution of antimicrobial susceptibility and resistance in circulating *Streptococcus pneumoniae*. Before PCV implementation, surveillance studies of pneumococcal carriage and invasive pneumococcal disease (IPD) revealed that PCV-targeted serotypes were more probable than non-vaccine serotypes to harbour lineages with diminished susceptibility or resistance to commonly-used antimicrobial drugs. One pre-licensure randomised controlled trial in South Africa and two post-licensure case-control studies in the USA revealed reduced risk for IPD involving antibiotic-resistant pneumococci among children who received PCVs compared with those who did not. Several multiyear surveillance studies of pneumococcal carriage and IPD, predominantly done in high-income settings, revealed increases in the proportion of non-vaccine-serotype pneumococci with reduced susceptibility or resistance to penicillin and macrolides. However, it is uncertain to what extent these patterns were reflected in other settings, and whether changes in susceptibility of circulating pneumococci are causally attributable to the implementation of PCVs rather than secular trends.

**Added value of this study**

We synthesised data from 559 distinct studies across 104 countries to characterise changes over time in susceptibility of pneumococcal isolates from paediatric nasopharyngeal carriage and IPD. Meta-regression models allowed us to control for effects of vaccination on susceptibility of pneumococcal isolates while controlling for heterogeneity in prevalence of non-susceptibility and resistance across regions and individual studies, for secular (PCV-independent) changes in isolate susceptibility over time, and for differences in isolate susceptibility across socioeconomically distinct settings. We identify evidence of reductions in non-susceptibility and resistance to penicillin, sulfamethoxazole-trimethoprim, and third-generation cephalosporins after PCV implementation. Although we identify smaller reductions in prevalence of resistance to tetracycline and macrolides, we did not identify clear changes in prevalence of non-susceptibility to these drugs, suggesting changes might have resulted from attenuation of resistant phenotypes or replacement of resistant by intermediate lineages. Stratified analyses revealed differential reductions in prevalence of penicillin non-susceptibility among vaccine-targeted and non-vaccine serotypes, but found no significant changes in prevalence of macrolide non-susceptibility among both serotype groups.

**Implications of all the available evidence**

Implementation of PCVs has led to reductions in non-susceptibility and resistance of pneumococci to first-line antibiotics used for treatment of community-acquired pneumonia. Post-vaccination changes in the antimicrobial susceptibility of circulating pneumococci should lead to reduced risk of treatment failure among children with pneumococcal diseases.

We assessed changes in antimicrobial susceptibility of paediatric pneumococcal isolates globally amid PCV introduction. We did a systematic review and meta-regression analysis examining susceptibility of pneumococci to various antibiotics, synthesising data from epidemiological studies done over two decades that included the initial rollout of PCVs in all settings where they are presently used.

## Methods

### Search strategy and selection criteria

We did a literature search (completed Nov 24, 2020) to identify studies published between Jan 1, 2000, and Nov 24, 2020, reporting antimicrobial susceptibility distributions in population-based samples of paediatric pneumococcal isolates from upper respiratory colonisation or invasive disease. We restricted our sample to carriage or invasive isolates because of the potential for study-to-study inconsistency in sources of isolates among patients with acute upper or lower respiratory infections (which could include nasopharyngeal isolates or isolates from sputa, middle ear fluid, conjunctiva, or sinus aspirates), and because recent antibiotic receipt among patients with these conditions could lead to bias. Inconsistent recommendations for adult vaccination with PCVs or pneumococcal polysaccharide vaccine, and variable availability of surveillance data from adult age groups, prevented similarly representative assessments of antimicrobial resistance prevalence among pneumococcal isolates from adults.

We searched for articles using the PubMed database, supplemented with data on IPD isolates captured by the Antimicrobial Testing Leadership and Surveillance (ATLAS; Pfizer) database. ATLAS is a global bacterial surveillance programme monitoring antimicrobial resistance longitudinally and across countries, including those where post-licensure epidemiological studies might not yet have been undertaken or published. A detailed description of the search terms is included in the appendix (pp 3–7).

We included English-language studies reporting primary antimicrobial susceptibility data for pneumococcal isolates obtained from children (aged 0–18 years) experiencing nasopharyngeal pneumococcal colonisation or IPD. Studies were ineligible if reporting was premised on findings from any particular assay, including studies of isolates of select serotypes (eg, only serotype 19A isolates), sequence types, susceptibility profiles, or other preselected microbiological attributes. We further excluded studies that did not specify either quantitative susceptibility data or provide references to standardised breakpoint definitions, because this would prevent us from verifying whether outcomes of non-susceptibility and resistance were met.

We included carriage data if studies obtained nasopharyngeal isolates from children who were asymptomatic, or who were not recruited on the basis of being diagnosed with any respiratory condition or symptoms. We included IPD data from primary studies and ATLAS database entries if isolates were obtained from blood, cerebrospinal fluid, or pleural fluid of patients diagnosed with meningitis, sepsis, or bacteraemic pneumonia. We excluded reviews, duplicate publications of individual datasets, and other articles that did not present primary data (appendix p 9). Although we primarily sought to include data from non-interventional studies, data from inactive (ie, standard-of-care) groups of interventional studies were eligible for inclusion if the above criteria were met.

We extracted susceptibility phenotype data on all drugs for which information was available. Based on an initial screen of data availability, we defined the proportion of isolates showing non-susceptibility or resistance to penicillin, erythromycin or other macrolides, sulfamethoxazole–trimethoprim, third-generation cephalosporins, and tetracycline as primary endpoints.

We defined susceptible, intermediate, and resistant classifications based on 2019 Clinical & Laboratory Standards Institute (CLSI) guidelines (appendix p 15); we considered intermediate and resistant phenotypes to be non-susceptible. To avoid changes in definitions associated with the 2009 revision of CLSI standards, and for consistency with other international standards (eg, European Committee on Antimicrobial Susceptibility Testing), we defined penicillin susceptibility according to oral, non-meningitis breakpoints. For studies assessing susceptibility based on phenotypic assays, we verified that breakpoint definitions matched CLSI standards.

### Data analysis

KA and BR screened abstracts and reviewed full texts in consultation with each other and the corresponding author, and recorded and verified reasons for exclusion. JAL made final decisions on study eligibility in the event of uncertainty or disagreement between reviewers. We abstracted data into a Microsoft Excel spreadsheet. For each article, we recorded features including country, study start and end dates, study population, participant ages, isolate source (IPD or carriage), and antibiotic susceptibility testing method and breakpoint definitions (if quantitative assay data were not presented). For each drug class, we extracted the number of susceptible, intermediate, and resistant (or susceptible and non-susceptible) isolates, and pneumococcal serotype (if available). We used the publicly available tool WebPlotDigitizer (version 4.2) to extract numerical data where studies presented results graphically. Where data were presented for multiple years or countries, we extracted data at the most granular level available, recording a single study identifier for all substrata. We used one study identifier for all data abstracted from ATLAS.

To define whether studies described PCV-exposed or PCV-naive populations, we searched the VIEW-Hub database from the International Vaccine Access Center and extracted country-specific PCV introduction dates, PCV7/10/13 product choices, and dates of changes in PCV product (appendix pp 24–32). We also collected per-capita gross domestic product (GDP; measured in constant 2010 US$) in each country for each year from the World Bank database (appendix pp 16–19). Due to limited data availability for longitudinal comparisons within individual countries, we grouped countries into 21 regions and seven super-regions consistent with standard Global Burden of Disease (GBD) designations (appendix pp 16–19).^16^

We did a meta-analysis of data from all studies that met the inclusion criteria. For descriptive presentations, we summed the number of unique studies and isolates with data available for each drug class, geographical region, isolate source (carriage and IPD), and vaccine exposure (PCV-exposed and PCV-naive populations). As a sensitivity analysis, we also generated sums and proportions excluding ATLAS database isolates (appendix pp 33–34).

We first summarised regional prevalence of non-susceptibility and resistance to penicillin and macrolides among colonising and invasive isolates in PCV-naive populations and populations with established PCV programmes. We defined prevalence as the proportion of pneumococcal isolates non-susceptible or resistant to a drug, among all pneumococcal isolates within a relevant sampling stratum (eg, carriage or invasive source, geographical region). To evaluate the effect of PCV compared with no PCV use, we restricted the eligible sample in this analysis to isolates from studies that concluded specimen collection at least 1 year before, or initiated specimen collection at least 3 years after, PCV implementation in each country. Insufficient data were available to permit similar stratification of estimates for other drug classes. We reformatted study-level aggregated data as isolate-level line lists and used linear regression models, accounting for study-level random effects, to estimate prevalence of non-susceptibility and resistance to each drug among pneumococci. We defined strata as GBD regions or super-regions, pre-PCV or post-PCV implementation time period (with respect to any PCV product), and colonising or invasive isolate. Where only one study was available, we computed 95% Cls using the Clopper-Pearson method. We included strata in this analysis only if data were available from at least 20 isolates. Insufficient data were available for comparisons of the prevalence of non-susceptibility or resistance among colonising or invasive isolates within individual countries before and after PCV implementation (appendix pp 21–23).

We next tested hypotheses about differences in the susceptibility of circulating pneumococci before and after PCV implementation. We estimated the adjusted risk difference (and its accompanying 95% CI) for pneumococci to be non-susceptible or resistant to each drug, based on the number of years after PCV implementation. To enable comparisons of changes in the proportions of non-susceptible and resistant isolates, we restricted the analysis to studies that distinguished intermediate and resistant isolates; we included data from all studies reporting susceptibility in a secondary analysis (appendix pp 12, 42).

We fit regression models accounting for random effects at the level of individual studies and GBD regions. We included fixed effects for invasive or carriage isolate source, the year of data collection (or midpoint year for multiyear studies), the country's per-capita GDP (log transformed), and changepoint terms, formulated as the time from vaccine implementation within the country to the initiation of sampling in the study. Values of the post-vaccination changepoint variable were set to zero for pre-vaccination samples. We used a square root transformation of years after PCV implementation to account flexibly for stabilising trends after vaccine implementation, and to avoid overfitting associated with use of higher-order polynomials in the short time period available for analysis (appendix p 10). To report overall changes, we sampled from estimates of the expected absolute difference in prevalence of non-susceptibility or resistance for each drug after 10 years of PCV use.

Finally, we sought to assess changes in antimicrobial resistance prevalence over time among serotypes exposed to vaccine-driven population immunity and among those not contained in PCV products. We defined vaccine-targeted serotypes as the serotypes included in each PCV product formulation (PCV7/10/13) being used in the national immunisation programmes in each country at the initiation of sample collection for a given study (appendix pp 24–32). We repeated meta-regression analyses described above using stratified datasets for non-susceptibility to penicillin and macrolides among PCV-targeted and non-PCV isolates (based on PCV product choices for each country and year). For each serotype, the changepoint term was formulated as years of use of a PCV product for which the serotype could be considered a vaccine-targeted or non-vaccine serotype. Insufficient serotype-level data were available for similar subgroup analyses of other drug classes.

We conducted analyses using R software (version 3.2.3). We fit random effects models using the lme4 package.

### Role of the funding source

The funder of the study played no role in study design, data collection, data analysis, data interpretation, or the writing of the report.

## Results

Of the 4910 studies screened for inclusion, we extracted data from 559 eligible studies (including the ATLAS database) reporting on susceptibility of 312 783 isolates across 104 countries (table 1; appendix pp 11, 20, 45–58) The ATLAS database contributed 14 162 isolates across 58 countries. Studies offered high coverage of both invasive and colonising isolates. Most data (258 [46·2%] of 559 studies; 232 623 [74·4%] of 312 783 isolates) were collected in high-income settings, with 139 studies (supplying 167 829 isolates) done in western Europe. Relative to the populations of these regions, Latin America and the Caribbean; sub-Saharan Africa; north Africa and the Middle East; south Asia; and southeast Asia, east Asia, and Oceania were under-represented in the final set of included studies and isolates. No eligible studies presented data from central Asia. Most data (395 [70·7%] of 559 studies; 214 210 [68·4%] of 312 783 isolates) were collected in PCV-naive populations.

**Table 1:** Features of included studies

		**Studies (n=559)**	**Isolates (n=312 783)**
Global Burden of Disease region and super-region*			
High income			
	Southern Latin America	11	13 300
	Western Europe	139	167 829
	High-income North America	62	35 329
	Australasia	17	9659
	High-income Asia Pacific	32	6506
	All countries	258	232 623
Latin America and Caribbean			
	Caribbean	4	414
	Central Latin America	17	3875
	Tropical Latin America	25	5248
	Andean Latin America	7	2069
	All countries	52	11 606
Sub-Saharan Africa			
	Southern sub-Saharan Africa	10	13 664
	Western sub-Saharan Africa	20	2230
	Central sub-Saharan Africa	4	791
	Eastern sub-Saharan Africa	36	6442
	All countries	68	23 127
North Africa and Middle East			
	All countries	55	7692
South Asia			
	All countries	26	5999
Southeast Asia, east Asia, and Oceania			
	East Asia	54	10 767
	Southeast Asia	23	5447
	Oceania	3	967
	All countries	79	17 181
Central and eastern Europe			
	Eastern Europe	10	4694
	Central Europe	21	9861
	All countries	31	14 555
**Isolate source**			
Invasive disease		271	181 119
Carriage		313	131 664
**Timing relative to vaccine introduction**			
Exclusively before		395	214 210
Exclusively after		83	23 364
Mixed		144	75 209
**Susceptibility testing method**†			
Disk diffusion		241	107 720
E-test		221	149 896
Broth microdilution		112	71 688
Agar dilution		46	13 068
Other		15	5486
Not specified		47	23 906
**Susceptibility standards**			
CLSI		471	278 574
EUCAST		47	21 651
Other		23	6170
Quantitative data or breakpoints provided		18	6387

CLSI=Clinical & Laboratory Standards Institute. EUCAST=European Committee on Antimicrobial Susceptibility Testing.

* We indicate countries belonging to each Global Burden of Disease region and super-region in the appendix (pp 16–19).

† Certain studies used multiple methods to determine susceptibility; thus, the number of isolates for which each method was applied does not necessarily equate to the total number of isolates included in the meta-analysis.

In unadjusted analyses, the proportion of isolates found to be non-susceptible or resistant varied across regions for each drug (table 2). For penicillin, macrolides, third-generation cephalosporins, and tetracycline, the greatest prevalence of non-susceptibility and resistance occurred among studies in southeast Asia, east Asia, and Oceania. The proportion of isolates non-susceptible or resistant to sulfamethoxazole–trimethoprim was highest in south Asia, followed closely by sub-Saharan Africa. High-income settings had the lowest proportion of isolates non-susceptible or resistant to penicillin, sulfamethoxazole–trimethoprim, and tetracycline, although findings varied substantially across subregions. High-income Asia-Pacific countries had among the highest prevalence of non-susceptibility and resistance to all drugs across all subregions, except sulfamethoxazole–trimethoprim and third-generation cephalosporins. Notable differences were observed in overall proportions of isolates non-susceptible and resistant to each drug class when excluding isolates identified via the ATLAS database (table 2; appendix pp 33–34).

**Table 2:** Total non-susceptible and resistant isolates included, by drug class and Global Burden of Disease region

	**Penicillin**		**Macrolides**		**Sulfamethoxazole- trimethoprim**		**Third-generation cephalosporins**		**Tetracycline**	
	Non-susceptible	Resistant	Non-susceptible	Resistant^1^	Non-susceptible	Resistant	Non-susceptible	Resistant	Non-susceptible	Resistant
**High income**										
Southern Latin America	3592 (38%)	1536 (16%)	152 (9%)	233 (6%)	..	929 (49%)	9 (10%)	55 (11%)	37 (41%)	28 (31%)
Western Europe	16 346 (12%)	3117 (8%)	12 434 (24%)	10 640 (20%)	5840 (26%)	2433 (18%)	1375 (5%)	153 (1%)	3393 (16%)	2552 (16%)
High-income North America	5228 (23%)	2013 (10%)	2921 (29%)	1912 (31%)	2789 (34%)	1306 (22%)	1873 (17%)	208 (6%)	667 (14%)	635 (15%)
Australasia	1200 (17%)	345 (8%)	699 (16%)	565 (19%)	1226 (39%)	1174 (36%)	270 (11%)	94 (3%)	347 (11%)	343 (10%)
High-income Asia Pacific	2787 (51%)	1457 (34%)	3134 (86%)	1971 (80%)	1201 (57%)	445 (35%)	1010 (26%)	139 (10%)	1491 (83%)	1434 (79%)
All countries	29 153 (16%)	8468 (11%)	19 340 (27%)	15 321 (23%)	11 056 (31%)	6287 (24%)	4537 (10%)	649 (2%)	5935 (19%)	4992 (20%)
**Latin America and Caribbean**										
Caribbean	46 (18%)	55 (25%)	0 (0%)	91 (37%)	..	93 (40%)	1 (1%)	1 (33%)	2 (67%)	13 (30%)
Central Latin America	1165 (47%)	240 (9%)	486 (29%)	438 (19%)	486 (61%)	747 (40%)	111 (15%)	19 (4%)	81 (24%)	36 (33%)
Tropical Latin America	1457 (31%)	187 (6%)	166 (13%)	130 (18%)	815 (71%)	331 (44%)	46 (4%)	12 (2%)	124 (22%)	65 (19%)
Andean Latin America	574 (28%)	118 (9%)	419 (28%)	287 (21%)	802 (60%)	651 (54%)	65 (8%)	12 (1%)	409 (50%)	340 (41%)
All countries	3242 (34%)	600 (8%)	1071 (24%)	946 (21%)	2103 (64%)	1822 (45%)	223 (8%)	44 (2%)	616 (36%)	454 (34%)
**Sub-Saharan Africa**										
Southern sub-Saharan Africa	5261 (45%)	546 (27%)	223 (21%)	140 (7%)	462 (49%)	95 (18%)	339 (13%)	0 (0%)	60 (38%)	226 (11%)
Western sub-Saharan Africa	357 (24%)	202 (12%)	67 (8%)	151 (11%)	414 (59%)	759 (63%)	20 (2%)	32 (4%)	519 (63%)	690 (61%)
Central sub-Saharan Africa	293 (37%)	30 (7%)	63 (8%)	33 (4%)	443 (57%)	418 (54%)	61 (37%)	20 (12%)	323 (42%)	253 (33%)
Eastern sub-Saharan Africa	1575 (28%)	302 (6%)	346 (7%)	284 (6%)	3612 (76%)	3158 (64%)	11 (0%)	4 (0%)	919 (25%)	673 (22%)
All countries	7486 (38%)	1080 (12%)	699 (9%)	608 (7%)	4931 (69%)	4430 (60%)	431 (7%)	56 (1%)	1821 (33%)	1842 (27%)
**North Africa and Middle East**										
All countries	1379 (28%)	1804 (34%)	737 (34%)	1048 (26%)	651 (55%)	1264 (54%)	190 (11%)	64 (3%)	550 (39%)	716 (40%)
South Asia										
All countries	274 (7%)	209 (34%)	377 (19%)	485 (11%)	1632 (73%)	3444 (74%)	32 (2%)	18 (3%)	334 (33%)	831 (56%)
**Southeast Asia, east Asia, and Oceania**										
East Asia	4404 (56%)	1930 (32%)	3976 (88%)	4622 (89%)	1586 (74%)	1909 (70%)	995 (29%)	886 (19%)	1231 (83%)	1333 (80%)
Southeast Asia	1574 (32%)	426 (16%)	1163 (44%)	1523 (36%)	1189 (53%)	953 (28%)	385 (13%)	33 (1%)	1302 (57%)	1235 (35%)
Oceania	181 (19%)	39 (9%)	4 (1%)	3 (1%)	65 (15%)	34 (8%)	3 (1%)	2 (1%)	4 (4%)	4 (4%)
All countries	6159 (45%)	2395 (26%)	5143 (68%)	6148 (63%)	2840 (59%)	2896 (44%)	1383 (21%)	921 (12%)	2537 (65%)	2572 (49%)
**Central and eastern Europe**										
Eastern Europe	493 (16%)	501 (12%)	209 (7%)	467 (21%)	647 (65%)	1007 (50%)	24 (43%)	0 (0%)	132 (33%)	156 (23%)
Central Europe	1027 (49%)	409 (17%)	510 (32%)	2781 (32%)	171 (80%)	690 (46%)	63 (6%)	1 (0%)	181 (47%)	489 (33%)
All countries	1520 (29%)	910 (14%)	719 (16%)	3248 (30%)	818 (67%)	1697 (48%)	87 (8%)	1 (0%)	313 (40%)	645 (30%)
**All regions**										
All countries	96 773 (21%)	28 917 (13%)	55 058 (28%)	54 065 (25%)	45 779 (42%)	38 931 (38%)	13 544 (11%)	3424 (4%)	23 328 (26%)	22 557 (26%)

Data are n (%). Non-susceptible isolates included all those with an intermediate or resistant phenotype. The proportion of isolates found to be resistant might be higher than the proportion of isolates found to be non-susceptible as a different body of studies, undertaken in different countries or populations, might have presented information on isolate non-susceptibility or resistance.

**Figure 1:**
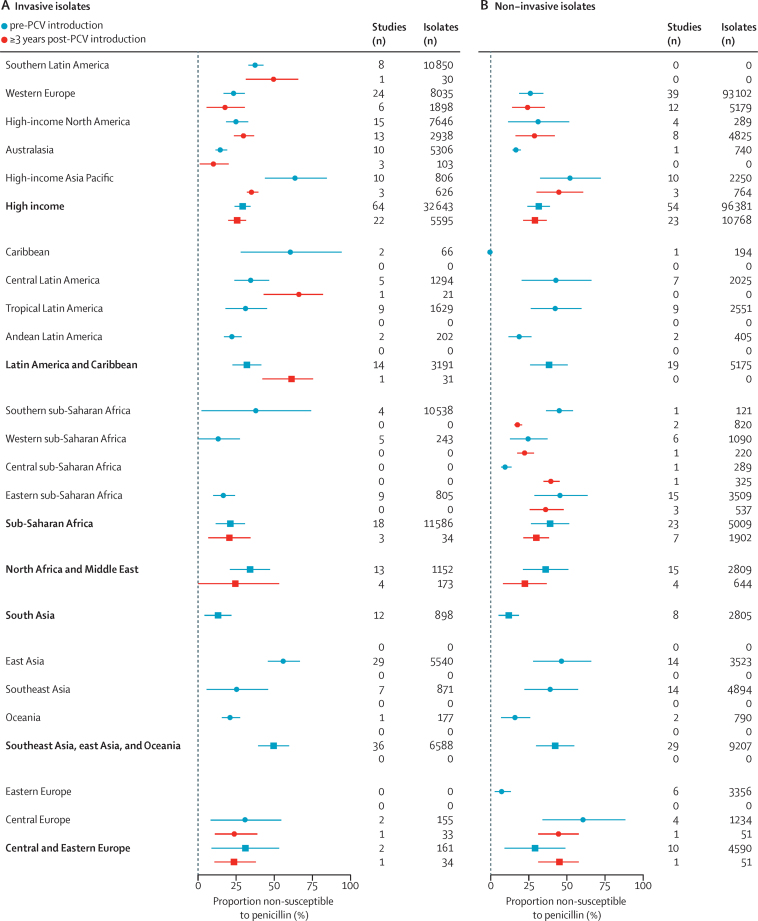
Prevalence of non-susceptibility to penicillin in invasive and non-invasive *Streptococcus pneumoniae* isolates before and after PCV implementation, by Global Burden of Disease region and super-region Data are median (95% CI); pooled estimates for each region account for study-level random effects. Numerical estimates are reported in the appendix (pp 35–36). PCV=pneumococcal conjugate vaccine.

**Figure 2:**
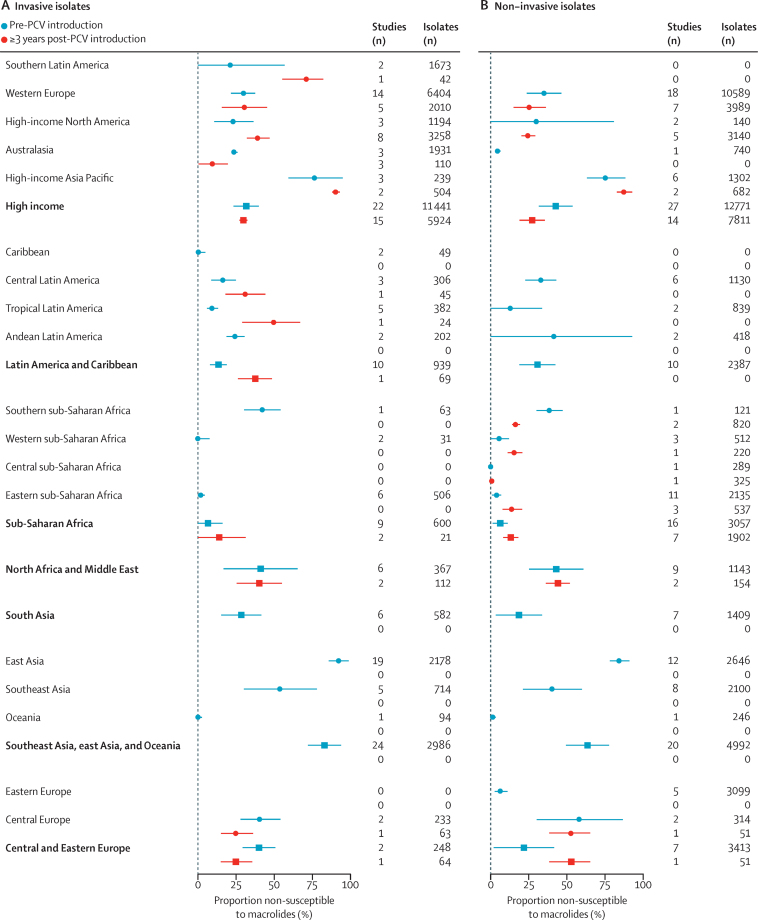
Prevalence of non-susceptibility to macrolides in invasive and non-invasive *Streptococcus pneumoniae* isolates, before and after PCV implementation, by Global Burden of Disease region and super-region Data are median (95% CI); pooled estimates for each region account for study-level random effects. Numerical estimates are reported in the appendix (pp 37–38). PCV=pneumococcal conjugate vaccine.

Random effects models stratifying further for invasive or carriage isolates and pre-PCV or post-PCV implementation time period provided a clearer view into regional differences in susceptibility to penicillin and macrolides (Figure 1, Figure 2; appendix pp 35–38). In PCV-naive populations, pooled estimates of the prevalence of penicillin non-susceptibility among invasive isolates ranged from 13·1% (95% CI 4·3–21·9) in south Asia to 56·3% (45·7–66·9) in east Asia, 61·2% (28·1–94·2) in the Carribean (although this estimate was only informed by two studies), and 64·2% (43·8–84·4) in high-income Asia-Pacific countries. Point estimates generally suggested a higher prevalence of penicillin non-susceptibility among colonising than invasive isolates. We did not identify consistent evidence of differences in the prevalence of non-susceptibility before and after PCV implementation within regional strata, although pre-implementation and post-implementation studies were not necessarily undertaken in the same countries or populations within each region (appendix pp 21–23).

We identified greater regional differences in prevalence of macrolide non-susceptibility (figure 2; appendix pp 37–38). In PCV-naive populations, a majority of invasive isolates were non-susceptible to macrolides in the southeast Asia, east Asia, and Oceania region (83·2%, 95% CI 72·3–94·1); however, 0% (0–2·6%) prevalence of non-susceptibility was observed within the Oceania subregion. Before PCV implementation, invasive isolates were also non-susceptible to macrolides in high-income Asia-Pacific countries (77·3%, 59·5–95·1), compared with 13·5% (8·0–19·0) in Latin America and Caribbean countries, and 6·6% (0·0–16·2) in sub-Saharan African countries. Similar to findings for penicillin, point estimates generally suggested a higher prevalence of macrolide non-susceptibility among colonising isolates, as compared with invasive isolates. Higher prevalence of macrolide non-susceptibility within each region was moderately associated with higher per-capita consumption of macrolides within countries where antibiotic consumption data were available, both before (Spearman's correlation coefficient ρ=0·39) and after (ρ=0·43) PCV implementation (appendix p 13). Non-susceptibility to penicillin was not associated with consumption (appendix p 14).

**Figure 3:**
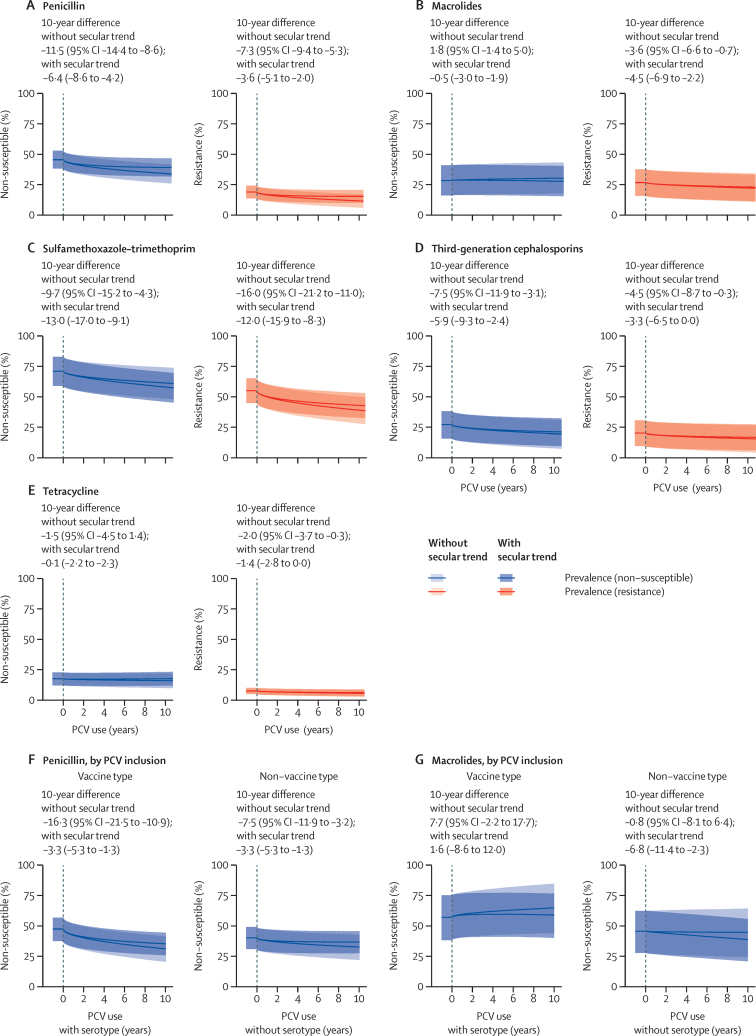
Changes in susceptibility to various drug classes, among all isolates (A–E) and by PCV inclusion (F–G) over a 10-year period after PCV implementation Data are median (95% CI). Meta-regression estimates are adjusted for years passing since vaccine implementation, calendar time (to account for secular trends independent of vaccine implementation), invasive or non-invasive isolate source, and country wealth. Plotted estimates correspond to expected changes in susceptibility among non-invasive pneumococcal isolates sampled from children in a hypothetical country with per-capita gross domestic product equal to US$22 000. (A–E) Includes studies that distinguished intermediate and resistant phenotypes (297 studies and 719 322 isolates). (F–G) Susceptibility to penicillin and macrolides for serotypes included in or not included in vaccine product formulas. In the appendix, we summarise data availability and list coefficient estimates for each covariate included in regression models (pp 40–41). Data are restricted to the corpus of studies presenting data on both non-susceptible and resistant isolates (297 studies); analyses of data from all studies are presented in the appendix (pp 12, 42). PCV=pneumococcal conjugate vaccine.

We next measured changes in susceptibility of isolates after PCV implementation, using meta-regression models to account for confounding factors, as well as heterogeneity across GBD regions and individual studies (figure 3A–E). We identified significant reductions in the prevalence of non-susceptibility and resistance to penicillin, third-generation cephalosporins, and sulfamethaxazole-trimethoprim among all pneumococcal isolates after vaccine implementation. We also identified reductions in the prevalence of tetracycline resistance. We did not find evidence of a change in the prevalence of macrolide non-susceptibility among pneumococcal isolates. Accounting for secular trends attenuated our estimates of the magnitude of reductions in the prevalence of non-susceptibility and resistance to penicillin and third-generation cephalosporin isolates, although post-vaccination trend estimates remained statistically significant.

Estimated changes in non-susceptibility were similar in a sensitivity analysis, including studies that did not distinguish intermediate and resistant phenotypes (appendix pp 12, 42). These analyses also identified significant reductions in the prevalence of non-susceptibility and resistance to penicillin and tetracycline, in addition to finding significant reductions in the prevalence of resistance to sulfamethoxazole–trimethoprim, third-generation cephalosporins, tetracycline, and macrolides in analyses without the inclusion of a secular trend (appendix p 12).

At the time of introduction of each PCV product, we estimated 7·6% (95% CI 4·5–10·8) higher absolute prevalence of non-susceptibility to penicillin and 11·5% (6·7–16·4) higher absolute prevalence of non-susceptibility to macrolides among serotypes included in vaccine formulations than among those not included (appendix p 40). Although we identified reductions in the prevalence of penicillin non-susceptibility among both vaccine-targeted and non-vaccine targeted serotypes, the magnitude of change was greater among vaccine-targeted serotypes (figure 3F–G). Accounting for secular trends reduced the magnitude of estimated changes in the prevalence of penicillin non-susceptibility for both vaccine-targeted and non-vaccine serotypes. We did not observe significant changes in the prevalence of macrolide non-susceptibility among both vaccine-targeted and non-vaccine serotypes after PCV implementation, consistent with our analysis of all isolates. Accounting for secular trends led to an overall reduction in the prevalence of macrolide non-susceptibility among non-vaccine serotype isolates.

## Discussion

In pooled analyses of data from 559 epidemiological studies across 104 countries, we identified reductions in prevalence of non-susceptibility and resistance to penicillin, sulfamethoxazole–trimethoprim, and third-generation cephalosporins among circulating pneumococci after implementation of PCVs. Point estimates of the reductions in prevalence of resistance to macrolides, tetracycline, and sulfamethoxazole–trimethoprim exceeded estimates of the reductions in prevalence of non-susceptibility to the same drugs, suggesting reversion from resistant to intermediate antimicrobial resistance phenotypes or replacement of resistant lineages by those with intermediate non-susceptibility. To reduce risk of confounding, these analyses controlled for study-level, country-level, and regional-level sources of heterogeneity, as well as secular trends in isolate susceptibility. Taken together, our findings are consistent with the hypothesis that implementation of PCVs has altered the antimicrobial susceptibility of circulating pneumococci, leading to reduced prevalence of non-susceptibility or resistance to drugs commonly used in the treatment of pneumonia and other diseases.

In our analyses, implementation of a PCV product was associated with long-term reductions in prevalence of penicillin non-susceptibility among serotypes that were included in local vaccine formulations, and to a lesser extent among non-vaccine serotypes. These changes might reflect reductions in betalactam consumption due to the prevention of acute otitis media and other respiratory infections through PCV use.^3^, ^4^ However, mechanisms accounting for differential effects among vaccine-targeted and non-vaccine serotypes merit close attention. Replacement of PCV-targeted serotypes by non-PCV serotypes, which had lower pre-vaccination prevalence of non-susceptibility in our study, might allow greater relative frequencies of exposure of non-vaccine serotypes to antibiotic treatment.^17^ Repeated cross-sectional studies in Massachusetts (USA) revealed progressive increases in antimicrobial resistance prevalence among non-vaccine serotypes after PCV implementation;^18^ our findings did not confirm similar increases globally, although many settings included in our study had shorter histories of PCV use. The absence of reductions in macrolide non-susceptibility among both PCV targeted and non-PCV serotypes in our study might reflect increasing use of macrolides globally, including azithromycin.^14^, ^19^, ^20^, ^21^

The burden of severe disease outcomes and deaths attributable to resistance in pneumococci remains incompletely understood. In high-income settings, some studies powered to control for differences in risk of death across serotypes, clinical presentations, and patient risk factors have reported higher risk of mortality in pneumonia and IPD among patients with resistant infections.^22^ However, ready access to second-line treatments and high-quality health care might reduce the incremental effects of resistance in high-income settings relative to its effects in settings with constrained access to second-line antibiotics and weaker health-care systems. Studies of meningitis^23^ and otitis media^24^ have reported higher risk of complications and severe sequelae among patients with treatment failure due to antimicrobial resistance, further demonstrating potential clinical implications of treatment failure.

Oral amoxicillin for 3–5 days is the primary treatment recommended by WHO for paediatric community-acquired pneumonia; ceftriaxone is recommended as a second-line treatment for children with severe pneumonia experiencing treatment failure.^25^ Our analysis suggests changes in pneumococcal susceptibility after PCV implementation should reduce risk of treatment failure among children who receive recommended therapies. Although macrolides are commonly used to treat pneumonia in various settings, this practice is not recommended by WHO;^25^ guidelines including macrolide treatment of childhood pneumonia limit this recommendation to children aged 60 months or older, children with atypical pathogens suspected, or children with previous non-response to treatment.^26^ Our findings suggest that empirical therapy with erythromycin, azithromycin, and clarithromycin remains suboptimal for paediatric pneumonia cases with suspected pneumococcal cause.^27^ Both ceftriaxone and macrolides are classified as Watch group antibiotics by WHO in recognition of concerns about emerging resistance to these drugs.^28^

Our study has several limitations. Insufficient data were available to address susceptibility to fluoroquinolones, lincosamides, vancomycin, and multidrug resistance. Although meta-regression provided a strategy to account for study-level and regional-level sources of heterogeneity, factors such as PCV coverage within countries were not addressed. Aggregating studies by region precluded meaningful evaluation of intra-country differences in isolate susceptibility amid vaccine implementation. Additionally, global trends summarising data across heterogeneous settings might be less sensitive to detecting changes in susceptibility than local studies. Further surveillance is needed to quantify effects of PCVs on antimicrobial susceptibility of pneumococci in low-income settings, where PCV programmes have shorter histories.^29^ Lastly, although it is difficult to ascertain publication bias in descriptive studies such as those we have assessed, investigators might have been more likely to publish findings documenting local changes in prevalence of non-susceptibility or resistance, because these would suggest a need for changes to treatment guidelines.

In addition to reducing mortality and severe complications associated with resistant pneumococcal infections, preserving susceptibility of pneumococci to first-line treatments might simplify treatment protocols for paediatric pneumonia and avert the need for use of last-resort antibiotics for this common condition. Although an important tool, PCVs have not eliminated the burden of disease associated with drug-resistant pneumococci, underscoring the need for further interventions to support antimicrobial stewardship.

For **data and code access on GitHub** see github.com/joelewnard/amrPneumo

## Data sharing

Data and code files to reproduce figures are available on GitHub.

## Declaration of interests

JAL has received grants and consulting fees from Pfizer and Merck Sharp & Dohme; and consulting fees from VaxCyte and Kaiser Permanente, unrelated to the submitted work. All other authors declare no competing interests.
