# Supplementary material for: Antimicrobial resistance in paediatric Streptococcus pneumoniae isolates amid global implementation of pneumococcal conjugate vaccines: a systematic review and meta-regression analysis
Source: Lancet Microbe. 2021 Sep;2(9):e450–60. doi: 10.1016/S2666-5247(21)00064-1 (PMC8410609; doi:10.1016/S2666-5247(21)00064-1)
Supplement: Supplementary appendix [file mmc1.pdf]

THE LANCET
Microbe

Supplementary appendix

This appendix formed part of the original submission and has been peer reviewed. We post it as supplied by the authors.

Supplement to: Andrejko K, Ratnasiri B, Hausdorff WP, Laxminarayan R, Lewnard JA. Antimicrobial resistance in paediatric *Streptococcus pneumoniae* isolates amid global implementation of pneumococcal conjugate vaccines: a systematic review and meta-regression analysis. *Lancet Microbe* 2021; published online June 22. https://doi.org/10.1016/S2666-5247(21)00064-1.

SUPPORTING INFORMATION

Prevalence of antimicrobial resistance in pediatric *Streptococcus pneumoniae* isolates amid global implementation of pneumococcal conjugate vaccines: a systematic review and meta-regression analysis

Kristin Andrejko, BS[1], Buddhika Ratnasiri, BA[2], William P. Hausdorff, PhD[3,4], Ramanan Laxminarayan, PhD[5,6], Joseph A. Lewnard, PhD[1,7,8]

1. Division of Epidemiology, School of Public Health, University of California, Berkeley, Berkeley, California, United States
2. College of Letters & Science, University of California, Berkeley, Berkeley, California, United States
3. PATH, Washington, D.C., United States
4. Faculty of Medicine, Université Libre de Bruxelles, Brussels, Belgium
5. Center for Disease Dynamics, Economics & Policy, New Delhi, India
6. Princeton Environmental Institute, Princeton University, Princeton, New Jersey, United States
7. Division of Infectious Diseases & Vaccinology, School of Public Health, University of California, Berkeley, Berkeley, California, United States
8. Center for Computational Biology, College of Engineering, University of California, Berkeley, Berkeley, California, United States

Contents

MESH search string for PubMed article search. 3

ATLAS database filtering criteria. 8

PRISMA exclusion criteria. 9

Meta-regression models. 10

Figure S1. PRISMA flowchart. 11

Figure S2. Post-vaccination changes in susceptibility to various drug classes. 12

Figure S3. Changes in susceptibility to macrolides (part A) and penicillin (part B) by country specific rates of consumption. 13

Table S1: Susceptibility breakpoints. 15

Table S2: Regional designations. 16

Table S3: Total number of studies included in systematic review by region. 20

Table S4: Summary of available studies ≥1 year before PCV introduction and ≥3 years after PCV introduction. 21

Table S5: Summary of PCV implementation status of the 104 countries included in the systematic review, sourced from ViewHub. 24

Table S6: Total non-susceptible and resistant isolates included, by drug class and Global Burden of Disease region, excluding ATLAS database. 33

Table S7: Stratified estimates of prevalence of non-susceptibility to penicillin, by region and isolate source. 35

Table S8: Stratified estimates of prevalence of non-susceptibility to macrolides, by region and isolate source. 37

Table S9: Meta-regression model parameters for analysis of pneumococcal isolates, restricted to corpus of studies distinguishing intermediate and resistant isolates (*N*=297). 39

Table S10: Total studies and isolates included in analyses of nonsusceptibility and resistance of vaccine-targeted and non-vaccine serotypes. 40

Table S11: Meta-regression model parameters for analysis of vaccine-targeted and non-vaccine serotype isolates. 41

Table S12: Meta-regression model parameters for analysis of all pneumococcal isolates. 42

Table S13: PRISMA checklist. 43

Table S14: Summary table of 558 studies included in systematic review and meta-analysis (excludes ATLAS database). 45

MESH search terms for PubMed article search

Search last updated November 24, 2020 by Kristin Andrejko

("Streptococcus pneumoniae"[All Fields]

OR

(pneumococ[All Fields] OR pneumococaal[All Fields] OR pneumococal[All Fields] OR pneumococc[All Fields] OR pneumococca[All Fields] OR pneumococcaemia[All Fields] OR pneumococcaemic[All Fields] OR pneumococcal[All Fields] OR pneumococcal'[All Fields] OR pneumococcall[All Fields] OR pneumococcalmeningitis[All Fields] OR pneumococcalpneumonia[All Fields] OR pneumococcalvaccine[All Fields] OR pneumococccal[All Fields] OR pneumococcccal[All Fields] OR pneumococcemia[All Fields] OR pneumococcemias[All Fields] OR pneumococcemic[All Fields] OR pneumococcemie[All Fields] OR pneumococcemies[All Fields] OR pneumococchi[All Fields] OR pneumococci[All Fields] OR pneumococci's[All Fields] OR pneumococcia[All Fields] OR pneumococcic[All Fields] OR pneumococcica[All Fields] OR pneumococcicas[All Fields] OR pneumococciche[All Fields] OR pneumococcicidal[All Fields] OR pneumococcicide[All Fields] OR pneumococcico[All Fields] OR pneumococcidal[All Fields] OR pneumococcie[All Fields] OR pneumococcies[All Fields] OR pneumococcique[All Fields] OR pneumococciques[All Fields] OR pneumococcis[All Fields] OR pneumococclea[All Fields] OR pneumococco[All Fields] OR pneumococcoal[All Fields] OR pneumococcol[All Fields] OR pneumococcosis[All Fields] OR pneumococcosuria[All Fields] OR pneumococcous[All Fields] OR pneumococctyper[All Fields] OR pneumococcu[All Fields] OR pneumococcuria[All Fields] OR pneumococcus[All Fields] OR pneumococcus'[All Fields] OR pneumococcus's[All Fields] OR pneumococcus19f[All Fields] OR pneumococcusbetegseg[All Fields] OR pneumococcusclinical[All Fields] OR pneumococcusfertozes[All Fields] OR pneumococcusok[All Fields] OR pneumococcusperitonitis[All Fields] OR pneumococcusprevencio[All Fields] OR pneumococcusuria[All Fields] OR pneumococcusvakcina[All Fields] OR pneumococcusvakcinacio[All Fields] OR pneumococcuswere[All Fields] OR pneumococcusx[All Fields] OR pneumococcy[All Fields] OR pneumococeal[All Fields] OR pneumocochlea[All Fields] OR pneumococi[All Fields] OR pneumococic[All Fields] OR pneumococica[All Fields] OR pneumococicas[All Fields] OR pneumococice[All Fields] OR pneumococicos[All Fields] OR pneumococilor[All Fields] OR pneumococique[All Fields] OR pneumococo[All Fields] OR pneumocococal[All Fields] OR pneumococococcal[All Fields] OR pneumococos[All Fields] OR pneumococul[All Fields] OR pneumococus[All Fields]))

AND

((resistan[All Fields] OR resistanc[All Fields] OR resistanca[All Fields] OR resistancce[All Fields] OR resistance[All Fields] OR resistance'[All Fields] OR resistance''[All Fields] OR resistance's[All Fields] OR resistance,[All Fields] OR resistance1[All Fields] OR resistance11[All Fields] OR resistance14[All Fields] OR resistance2[All Fields] OR resistance3[All Fields] OR resistance4[All Fields] OR resistance6[All Fields] OR resistance8[All Fields] OR resistance9[All Fields] OR resistanceamong[All Fields] OR resistanceand[All Fields] OR resistanceare[All Fields] OR resistanceassociated[All Fields] OR resistancebreaking[All Fields] OR resistancecaliber[All Fields] OR resistancecan[All Fields] OR resistanced[All Fields] OR resistancedetermining[All Fields] OR resistancedomaincontaining[All Fields] OR resistancedurability[All Fields] OR resistanceesophageal[All Fields] OR resistanceetween[All Fields] OR resistanceevolution[All Fields] OR resistancega[All Fields] OR resistancein[All Fields] OR resistanceindex[All Fields] OR resistanceir[All Fields] OR resistancekurven[All Fields] OR resistancel[All Fields] OR resistancelocuso[All Fields] OR resistancemap[All Fields] OR resistancemcr[All Fields] OR resistancemediated[All Fields] OR resistancemeter[All Fields] OR resistancenutrition[All Fields] OR resistanceof[All Fields] OR resistanceopen[All Fields] OR resistanceplus[All Fields] OR resistancerate[All Fields] OR resistancerelated[All Fields] OR resistanceright[All Fields] OR resistances[All Fields] OR resistances'[All Fields] OR resistancesim[All Fields] OR resistancesubmitted[All Fields] OR resistancesusceptibility[All Fields] OR resistancethis[All Fields] OR resistanceto[All Fields] OR resistancetype[All Fields] OR resistancevars[All Fields] OR resistancewas[All Fields] OR resistancewere[All Fields] OR resistancewith[All Fields] OR resistancex[All Fields] OR resistancexcompliance[All Fields] OR resistancexpert[All Fields] OR resistancia[All Fields] OR resistancies[All Fields] OR resistanct[All Fields] OR resistancto[All Fields] OR resistancy[All Fields] OR

resistand[All Fields] OR resistane[All Fields] OR resistanec[All Fields] OR resistaneza[All Fields] OR resistanfce[All Fields] OR resistange[All Fields] OR resistanhe[All Fields] OR resistanr[All Fields] OR resistans[All Fields] OR resistanse[All Fields] OR resistant[All Fields] OR resistant'[All Fields] OR resistant"[All Fields] OR resistant's[All Fields] OR resistant,[All Fields] OR resistant1[All Fields] OR resistant1's[All Fields] OR resistant11[All Fields] OR resistant12[All Fields] OR resistant2[All Fields] OR resistant2,3[All Fields] OR resistant3[All Fields] OR resistant30[All Fields] OR resistant4[All Fields] OR resistant41[All Fields] OR resistant5[All Fields] OR resistant6[All Fields] OR resistanta[All Fields] OR resistantacid[All Fields] OR resistantacinetobacter[All Fields] OR resistantance[All Fields] OR resistantassociated[All Fields] OR resistantb[All Fields] OR resistantc[All Fields] OR resistantcases[All Fields] OR resistantce[All Fields] OR resistantcell[All Fields] OR resistantcells[All Fields] OR resistante[All Fields] OR resistantenterococci[All Fields] OR resistantenterococcus[All Fields] OR resistantenterococcusdetected[All Fields] OR resistantes[All Fields] OR resistantescherichia[All Fields] OR resistantescherichiacolifrom[All Fields] OR resistantgastric[All Fields] OR resistantgroup[All Fields] OR resistanth[All Fields] OR resistanthypertension[All Fields] OR resistantiae[All Fields] OR resistantin[All Fields] OR resistantl[All Fields] OR resistantlike[All Fields] OR resistantlpr[All Fields] OR resistantly[All Fields] OR resistantm[All Fields] OR resistantmalaria[All Fields] OR resistantmedicago[All Fields] OR resistantmgrbmutants[All Fields] OR resistantmicroorganisms[All Fields] OR resistantmycobacterium[All Fields] OR resistantmycoplasma[All Fields] OR resistantn[All Fields] OR resistantneisseria[All Fields] OR resistantnicotiana[All Fields] OR resistantp[All Fields] OR resistantpathogens[All Fields] OR resistantpathogensidentified[All Fields] OR resistantphenotype[All Fields] OR resistantplants[All Fields] OR resistantplus[All Fields] OR resistantprimary[All Fields] OR resistantproteus[All Fields] OR resistantpseudomonas[All Fields] OR resistantpsuedomonas[All Fields] OR resistantr[All Fields] OR resistants[All Fields] OR resistantstaphylococcus[All Fields] OR resistantstrains[All Fields] OR resistantstreptococcus[All Fields] OR resistantttb[All Fields] OR resistantto[All Fields] OR resistanttodegradationby[All Fields] OR resistanttuberculosis[All Fields] OR resistantu[All Fields] OR resistantwomen[All Fields] OR resistanz[All Fields] OR resistanza[All Fields] OR resistanzce[All Fields] OR resistanzspektrum[All Fields])

OR

(susceptib[All Fields] OR susceptibbility[All Fields] OR susceptibble[All Fields] OR susceptibe[All Fields] OR susceptibel[All Fields] OR susceptibiblity[All Fields] OR susceptibie[All Fields] OR susceptibiity[All Fields] OR susceptibil[All Fields] OR susceptibile[All Fields] OR susceptibili[All Fields] OR susceptibilidad[All Fields] OR susceptibilidade[All Fields] OR susceptibilidades[All Fields] OR susceptibilies[All Fields] OR susceptibilify[All Fields] OR susceptibililly[All Fields] OR susceptibililties[All Fields] OR susceptibililty[All Fields] OR susceptibilily[All Fields] OR susceptibilit[All Fields] OR susceptibilita[All Fields] OR susceptibilitat[All Fields] OR susceptibilitate[All Fields] OR susceptibilitatea[All Fields] OR susceptibilitatii[All Fields] OR susceptibilite[All Fields] OR susceptibilites[All Fields] OR susceptibilities[All Fields] OR susceptibilities'[All Fields] OR susceptibilitities[All Fields] OR susceptibilitity[All Fields] OR susceptibilitiy[All Fields] OR susceptibilitv[All Fields] OR susceptibility[All Fields] OR susceptibility'[All Fields] OR susceptibility's[All Fields] OR susceptibility'testing[All Fields] OR susceptibility,[All Fields] OR susceptibility0020might[All Fields] OR susceptibility1[All Fields] OR susceptibility16[All Fields] OR susceptibility2[All Fields] OR susceptibility5[All Fields] OR susceptibilitygene[All Fields] OR susceptibilityof[All Fields] OR susceptibilityrelated[All Fields] OR susceptibilitystatus[All Fields] OR susceptibilityto[All Fields] OR susceptibilitywas[All Fields] OR susceptibilityweighted[All Fields] OR susceptibiliy[All Fields] OR susceptibillities[All Fields] OR susceptibillty[All Fields] OR susceptibilties[All Fields] OR susceptibiltity[All Fields] OR susceptibiltiy[All Fields] OR susceptibilty[All Fields] OR susceptibily[All Fields] OR susceptibitity[All Fields] OR susceptibity[All Fields] OR susceptibiulity[All Fields] OR susceptible[All Fields] OR susceptible'[All Fields] OR susceptible'p[All Fields] OR susceptible1[All Fields] OR susceptible3[All Fields] OR susceptibleinfected[All Fields] OR susceptiblel[All Fields] OR susceptibleleucaena[All Fields] OR susceptiblelycopersicon[All Fields] OR susceptiblemycobacterium[All Fields] OR susceptiblen[All Fields] OR susceptibleness[All Fields] OR susceptibles[All Fields] OR susceptibles'[All Fields] OR susceptiblet[All Fields] OR susceptibleto[All Fields] OR susceptiblexresistant[All Fields] OR susceptibliity[All Fields] OR susceptiblility[All Fields]

OR susceptiblilty[All Fields] OR susceptiblities[All Fields] OR susceptiblity[All Fields] OR susceptibly[All Fields])

OR

(sensitiv[All Fields] OR sensitiva[All Fields] OR sensitivae[All Fields] OR sensitivas[All Fields] OR sensitivatat[All Fields] OR sensitivation[All Fields] OR sensitivdty[All Fields] OR sensitive[All Fields] OR sensitive'[All Fields] OR sensitive''[All Fields] OR sensitive's[All Fields] OR sensitive1[All Fields] OR sensitive2[All Fields] OR sensitive21[All Fields] OR sensitive23[All Fields] OR sensitive23d[All Fields] OR sensitive3[All Fields] OR sensitive3a[All Fields] OR sensitive4[All Fields] OR sensitive5[All Fields] OR sensitive51[All Fields] OR sensitive51c[All Fields] OR sensitive52[All Fields] OR sensitive81[All Fields] OR sensitive9[All Fields] OR sensitivebiochemical[All Fields] OR sensitivebut[All Fields] OR sensitivecardiac[All Fields] OR sensitived[All Fields] OR sensitivedatura[All Fields] OR sensitiveefflux[All Fields] OR sensitivefsgs[All Fields] OR sensitivehsd[All Fields] OR sensitivelethal[All Fields] OR sensitively[All Fields] OR sensitivem[All Fields] OR sensitivemess[All Fields] OR sensitivemutants[All Fields] OR sensitivemutations[All Fields] OR sensitiven[All Fields] OR sensitivend[All Fields] OR sensitiveness[All Fields] OR sensitivenested[All Fields] OR sensitivepatient[All Fields] OR sensitivepro[All Fields] OR sensitiveprostate[All Fields] OR sensitiver[All Fields] OR sensitives[All Fields] OR sensitives'[All Fields] OR sensitivesensorial[All Fields] OR sensitiveshibire[All Fields] OR sensitiveslow[All Fields] OR sensitivetest[All Fields] OR sensitiveto[All Fields] OR sensitivetoolsforevaluating[All Fields] OR sensitivetu[All Fields] OR sensitivety[All Fields] OR sensitivi[All Fields] OR sensitivi'[All Fields] OR sensitividade[All Fields] OR sensitivie[All Fields] OR sensitiviertem[All Fields] OR sensitivies[All Fields] OR sensitiviity[All Fields] OR sensitivily[All Fields] OR sensitiviness[All Fields] OR sensitiving[All Fields] OR sensitivisation[All Fields] OR sensitivisering[All Fields] OR sensitivit[All Fields] OR sensitivita[All Fields] OR sensitivitas[All Fields] OR sensitivitat[All Fields] OR sensitivitats[All Fields] OR sensitivitatsanalyse[All Fields] OR sensitivitatsanderungen[All Fields] OR sensitivitatsindex[All Fields] OR sensitivitatssteigerung[All Fields] OR sensitivitatsstudie[All Fields] OR sensitivitatsstudien[All Fields] OR sensitivitatstest[All Fields] OR sensitivitatstestung[All Fields] OR sensitivitatsvergleich[All Fields] OR sensitivitatsverlust[All Fields] OR sensitivite[All Fields] OR sensitivites[All Fields] OR sensitivitet[All Fields] OR sensitivitets[All Fields] OR sensitivitetsforvirring[All Fields] OR sensitivitetskurser[All Fields] OR sensitivitetstranas[All Fields] OR sensitivitetstraning[All Fields] OR sensitivitetstraningen[All Fields] OR sensitivitetstrening[All Fields] OR sensitivitiable[All Fields] OR sensitivitied[All Fields] OR sensitivities[All Fields] OR sensitivities'[All Fields] OR sensitivitiesor[All Fields] OR sensitivitiness[All Fields] OR sensitivitites[All Fields] OR sensitivitities[All Fields] OR sensitivitity[All Fields] OR sensitivitive[All Fields] OR sensitivitives[All Fields] OR sensitivitivity[All Fields] OR sensitivitiy[All Fields] OR sensitivitty[All Fields] OR sensitivitv[All Fields] OR sensitivity[All Fields] OR sensitivity'[All Fields] OR sensitivity''[All Fields] OR sensitivity'comt[All Fields] OR sensitivity's[All Fields] OR sensitivity,[All Fields] OR sensitivity0[All Fields] OR sensitivity1[All Fields] OR sensitivity100[All Fields] OR sensitivity26[All Fields] OR sensitivity28[All Fields] OR sensitivity2x2xk[All Fields] OR sensitivity5[All Fields] OR sensitivity60[All Fields] OR sensitivityanalyses[All Fields] OR sensitivityand[All Fields] OR sensitivityc[All Fields] OR sensitivitycalizationand[All Fields] OR sensitivitycardiac[All Fields] OR sensitivitycasecontrol[All Fields] OR sensitivitycontrast[All Fields] OR sensitivityfor[All Fields] OR sensitivityfunction[All Fields] OR sensitivityhits[All Fields] OR sensitivityinconclusive[All Fields] OR sensitivityincreased[All Fields] OR sensitivitykij[All Fields] OR sensitivitylimit[All Fields] OR sensitivitymagnetic[All Fields] OR sensitivitymult[All Fields] OR sensitivityno[All Fields] OR sensitivityof[All Fields] OR sensitivitypowered[All Fields] OR sensitivityr[All Fields] OR sensitivitys[All Fields] OR sensitivitysers[All Fields] OR sensitivityspecificity[All Fields] OR sensitivityspecificitydiagnostic[All Fields] OR sensitivityspecificitypositive[All Fields] OR sensitivitytesting[All Fields] OR sensitivityto[All Fields] OR sensitivitytroponin[All Fields] OR sensitivityvery[All Fields] OR sensitivitywarfarin[All Fields] OR sensitivityxspecificity[All Fields] OR sensitivityxstress[All Fields] OR sensitivive[All Fields] OR sensitiviy[All Fields] OR sensitiviyy[All Fields] OR sensitivization[All Fields] OR sensitivized[All Fields] OR sensitivly[All Fields] OR sensitivnach[All Fields] OR sensitivnaia[All Fields] OR sensitivni[All Fields] OR sensitivniia[All Fields] OR sensitivnogo[All Fields] OR sensitivnom[All Fields] OR sensitivnosti[All Fields] OR sensitivnyi[All Fields] OR sensitivnym[All Fields] OR sensitivnymi[All Fields] OR sensitivo[All Fields] OR sensitivomoteurs[All Fields] OR sensitivomotor[All Fields] OR

sensitivomotora[All Fields] OR sensitivomotoras[All Fields] OR sensitivomotrice[All Fields] OR sensitivomotrices[All Fields] OR sensitivomotriz[All Fields] OR sensitivos[All Fields] OR sensitivovegetatif[All Fields] OR sensitivovegetatives[All Fields] OR sensitivrty[All Fields] OR sensitivste[All Fields] OR sensitivt[All Fields] OR sensitivtities[All Fields] OR sensitivtity[All Fields] OR sensitivty[All Fields] OR sensitivum[All Fields] OR sensitivus[All Fields] OR sensitivy[All Fields] OR sensitivyt[All Fields]))

AND

(carriage[All Fields]

OR

colonization[All Fields]

OR

(invasiv[All Fields] OR invasiva[All Fields] OR invasivally[All Fields] OR invasivas[All Fields] OR invasivasui[All Fields] OR invasive[All Fields] OR invasive'[All Fields] OR invasive"[All Fields] OR invasive1[All Fields] OR invasive1,2[All Fields] OR invasive3[All Fields] OR invasive3,4[All Fields] OR invasiveacacia[All Fields] OR invasiveapproach[All Fields] OR invasivebladder[All Fields] OR invasivebrain[All Fields] OR invasivecardiac[All Fields] OR invasivecollection[All Fields] OR invasived[All Fields] OR invasivediagnosis[All Fields] OR invasivedoc[All Fields] OR invasiveescherichia[All Fields] OR invasiveive[All Fields] OR invasiveless[All Fields] OR invasivelesslessbenefit[All Fields] OR invasivelly[All Fields] OR invasively[All Fields] OR invasively'[All Fields] OR invasivem[All Fields] OR invasivemethods[All Fields] OR invasivemodality[All Fields] OR invasivemole[All Fields] OR invasiven[All Fields] OR invasivencss[All Fields] OR invasivene[All Fields] OR invasivenes[All Fields] OR invasiveness[All Fields] OR invasiveness'[All Fields] OR invasivenessdagger[All Fields] OR invasivenesss[All Fields] OR invasivenoninvasive[All Fields] OR invasivenss[All Fields] OR invasiveo[All Fields] OR invasivep[All Fields] OR invasivepossible[All Fields] OR invasiver[All Fields] OR invasiverespiratory[All Fields] OR invasives[All Fields] OR invasives'[All Fields] OR invasivesess[All Fields] OR invasivesness[All Fields] OR invasivespeciesinfo[All Fields] OR invasivesurgery[All Fields] OR invasivetechniques[All Fields] OR invasivetka[All Fields] OR invasivetreatment[All Fields] OR invasivetype[All Fields] OR invasiveusing[All Fields] OR invasivewhile[All Fields] OR invasivi[All Fields] OR invasividad[All Fields] OR invasividade[All Fields] OR invasiviness[All Fields] OR invasivion[All Fields] OR invasivita[All Fields] OR invasivitat[All Fields] OR invasivitatsreduktion[All Fields] OR invasivite[All Fields] OR invasivitet[All Fields] OR invasivities[All Fields] OR invasivity[All Fields] OR invasivkardiologen[All Fields] OR invasivkardiologie[All Fields] OR invasivly[All Fields] OR invasivness[All Fields] OR invasivni[All Fields] OR invasivnykh[All Fields] OR invasivo[All Fields] OR invasivos[All Fields] OR invasivt[All Fields] OR invasivum[All Fields])

OR ("blood"[Subheading] OR "blood"[All Fields] OR "blood"[MeSH Terms]) OR CSF[All Fields] OR (cerebrospin[All Fields] OR cerebrospina[All Fields] OR cerebrospinaie[All Fields] OR cerebrospinaiis[All Fields] OR cerebrospinal[All Fields] OR cerebrospinala[All Fields] OR cerebrospinale[All Fields] OR cerebrospinalen[All Fields] OR cerebrospinaler[All Fields] OR cerebrospinales[All Fields] OR cerebrospinalfluessigkeit[All Fields] OR cerebrospinalfluid[All Fields] OR cerebrospinalflusigkeit[All Fields] OR cerebrospinalflussigkeit[All Fields] OR cerebrospinali[All Fields] OR cerebrospinalis[All Fields] OR cerebrospinalis1[All Fields] OR cerebrospinalisban[All Fields] OR cerebrospinalliquor[All Fields] OR cerebrospinalmeningitt[All Fields] OR cerebrospinalna[All Fields] OR cerebrospinalnaho[All Fields] OR cerebrospinalne[All Fields] OR cerebrospinalneho[All Fields] OR cerebrospinalni[All Fields] OR cerebrospinalniho[All Fields] OR cerebrospinalnim[All Fields] OR cerebrospinalnog[All Fields] OR cerebrospinalnoi[All Fields] OR cerebrospinalnoj[All Fields] OR cerebrospinalnom[All Fields] OR cerebrospinals[All Fields] OR cerebrospinalvaeske[All Fields] OR cerebrospinalvaeskelaekage[All Fields] OR cerebrospinalvaesken[All Fields] OR cerebrospinalvaeskens[All Fields] OR cerebrospinalvatska[All Fields] OR cerebrospinalvatskan[All Fields] OR cerebrospinla[All Fields] OR cerebrospinmal[All Fields]

OR cerebrospino[All Fields] OR cerebrospinous[All Fields] OR cerebrospinsl[All Fields] OR cerebrospinslnoj[All Fields])

OR

(nasopharyn[All Fields] OR nasopharyneal[All Fields] OR nasopharynectomy[All Fields] OR nasopharynegal[All Fields] OR nasopharynegeal[All Fields] OR nasopharyng[All Fields] OR nasopharyngaeal[All Fields] OR nasopharyngael[All Fields] OR nasopharyngaitis[All Fields] OR nasopharyngal[All Fields] OR nasopharynge[All Fields] OR nasopharyngea[All Fields] OR nasopharyngead[All Fields] OR nasopharyngeal[All Fields] OR nasopharyngeal'[All Fields] OR nasopharyngealbordetella[All Fields] OR nasopharyngealcarcinoma[All Fields] OR nasopharyngeale[All Fields] OR nasopharyngealelektroden[All Fields] OR nasopharyngealem[All Fields] OR nasopharyngealen[All Fields] OR nasopharyngealer[All Fields] OR nasopharyngeales[All Fields] OR nasopharyngealis[All Fields] OR nasopharyngeally[All Fields] OR nasopharyngealpassages[All Fields] OR nasopharyngealpodning[All Fields] OR nasopharyngealt[All Fields] OR nasopharyngeat[All Fields] OR nasopharyngectomies[All Fields] OR nasopharyngectomy[All Fields] OR nasopharyngee[All Fields] OR nasopharyngel[All Fields] OR nasopharyngeoscopy[All Fields] OR nasopharyngerl[All Fields] OR nasopharynges[All Fields] OR nasopharyngeum[All Fields] OR nasopharyngeus[All Fields] OR nasopharyngheal[All Fields] OR nasopharyngial[All Fields] OR nasopharyngien[All Fields] OR nasopharyngienne[All Fields] OR nasopharyngiens[All Fields] OR nasopharyngioma[All Fields] OR nasopharyngis[All Fields] OR nasopharyngital[All Fields] OR nasopharyngitidis[All Fields] OR nasopharyngitis[All Fields] OR nasopharyngitis'[All Fields] OR nasopharyngitises[All Fields] OR nasopharyngo[All Fields] OR nasopharyngoal[All Fields] OR nasopharyngobronchial[All Fields] OR nasopharyngoendoscopy[All Fields] OR nasopharyngofiberoscope[All Fields] OR nasopharyngofiberscopy[All Fields] OR nasopharyngogram[All Fields] OR nasopharyngograms[All Fields] OR nasopharyngography[All Fields] OR nasopharyngolarygnoscope[All Fields] OR nasopharyngolaryngeal[All Fields] OR nasopharyngolaryngee[All Fields] OR nasopharyngolaryngoscope[All Fields] OR nasopharyngolaryngoscopes[All Fields] OR nasopharyngolaryngoscopic[All Fields] OR nasopharyngolaryngoscopie[All Fields] OR nasopharyngolaryngoscopies[All Fields] OR nasopharyngolaryngoscopy[All Fields] OR nasopharyngolaryngovideoscope[All Fields] OR nasopharyngolarynx[All Fields] OR nasopharyngometry[All Fields] OR nasopharyngoscope[All Fields] OR nasopharyngoscopes[All Fields] OR nasopharyngoscopic[All Fields] OR nasopharyngoscopically[All Fields] OR nasopharyngoscopies[All Fields] OR nasopharyngoscopy[All Fields] OR nasopharyngoskop[All Fields] OR nasopharyngosopic[All Fields] OR nasopharyngotis[All Fields] OR nasopharyngral[All Fields] OR nasopharyngs[All Fields] OR nasopharyngscopy[All Fields] OR nasopharyngtis[All Fields] OR nasopharyngus[All Fields] OR nasopharyngx[All Fields] OR nasopharynlaryngoscope[All Fields] OR nasopharynogoscopy[All Fields] OR nasopharyns[All Fields] OR nasopharynx[All Fields] OR nasopharynx'[All Fields] OR nasopharynx's[All Fields] OR nasopharynx2[All Fields] OR nasopharynxandnasopharyngeal[All Fields] OR nasopharynxaspirat[All Fields] OR nasopharynxbefall[All Fields] OR nasopharynxduring[All Fields] OR nasopharynxelektrode[All Fields] OR nasopharynxes[All Fields] OR nasopharynxkarcinomer[All Fields] OR nasopharynxkarzinom[All Fields] OR nasopharynxkarzinome[All Fields] OR nasopharynxkarzinomen[All Fields] OR nasopharynxkarzinompatienten[All Fields] OR nasopharynxkarzinoms[All Fields] OR nasopharynxmalignom[All Fields] OR nasopharynxmalignome[All Fields] OR nasopharynxprozessen[All Fields] OR nasopharynxtuberkulos[All Fields] OR nasopharynxtuberkulose[All Fields] OR nasopharynxtumor[All Fields] OR nasopharynxtumoren[All Fields] OR nasopharynxtumors[All Fields] OR nasopharyny[All Fields] OR nasopharynyeal[All Fields] OR nasopharynz[All Fields]))

AND ("2000/01/01"[PDAT] : "2020/11/24"[PDAT])

ATLAS database filtering criteria

Search executed on 13 September, 2019 by Kristin Andrejko

Region: Worldwide
Pathogen: *Streptococcus pneumoniae*
Antimicrobial: Tetracycline, Penicillin, Ampicillin, Azithromycin, Clarithromycin, Erythromycin, Ceftriaxone, Clindamycin, Vancomycin, Levofloaxcin

Years: All Years

Data source: All

Breakpoints: CLSI

All Phenotypes
All Genotypes

Age: 0-18

All WARD sources (hospitalized patients)
All Resistance Patterns

Specimen Source:

Bodily Fluids
Bodily Fluids: Thoracentesis
Bodily Fluids: Peritoneal
Bodily Fluids: Pleural
Bodily Fluids: Exudate
Bodily Fluids: CSF
Bodily Fluids: Other
Bodily Fluids: Abscess / Pus
Bodily Fluids: Synovial
Bodily Fluids: Tissue
Bodily Fluids: Abdominal
Bodily Fluids: Bile
Bodily Fluids: Feces/Stool
Bodily Fluids: Pericardial

Central Nervous (CNS)
CNS: Brain
CNS: Spinal Cord
CNS: Peripheral Nerves
CNS: Other

CardioVascular (CVS)
CVS: Blood
CVS: Other
CVS: Blood Vessels
CVS: Heart

PRISMA Exclusion Criteria

<u>Age restrictions not met (n = 268)</u>

- Data not stratified by age (n = 223)
- No pediatric isolates (n = 45)

<u>Data on isolate susceptibility not presented/aggregated (n = 149)</u>

- No susceptibility data presented (n = 107)
- No denominator data presented (n = 32)
- Only multidrug resistant data presented (n = 10)

<u>Invasive and noninvasive criteria not met (n = 110)</u>

- Symptomatic nasopharyngeal isolates (n = 83)
- Data not stratified by invasive and noninvasive Isolates (n = 27)

<u>Microbiological/diagnostic criteria not met (n = 28)</u>

<u>Other (n = 119)</u>

- Not in English (n = 40)
- No primary data presented (n = 40)
- Full text inaccessible (n = 20)
- Case report (n = 12)
- Animal model (n = 4)
- Isolates not from *Streptococcus Pneumoniae* (n = 2)
- No sample collection dates provided (n = 1)

Meta-Regression models

Candidate models tested linear, quadratic, cubic and square root terms for years since vaccine introduction. Final models fit using the lme4() package in R include:

Figure 4:
lmer(ns ~ sqrt(YearsSinceVaxIntro) + MidpointYear + log(GDP) + IsolateType + (1|studyID) + (1|region) , data = data

Figure 5:
lmer(ns ~ MidpointYear + log(GDP) + IsolateType + VaccineType + sqrt(YearsSinceVaxIntro)*VaccineType + (1|studyID) + (1|region) , data = data

Where:

- YearsSinceVaxIntro: time elapsing between vaccine implementation within the country and the initiation of sampling in the study
- MidpointYear: calendar time, calculated as the median of sample collection to account for secular trends independent of time since vaccine implementation
- GDP: log per-capita gross domestic product [GDP], normalized to 2010 US dollars
- Isolate Type: whether isolate was invasive or non-invasive
- VaccineType: whether serotype was considered Vaccine Type or Non-Vaccine type based off of PCV product introduced in country at start year of sampling
- studyID: unique study identifier
- region: Global Burden of Disease region corresponding to each study

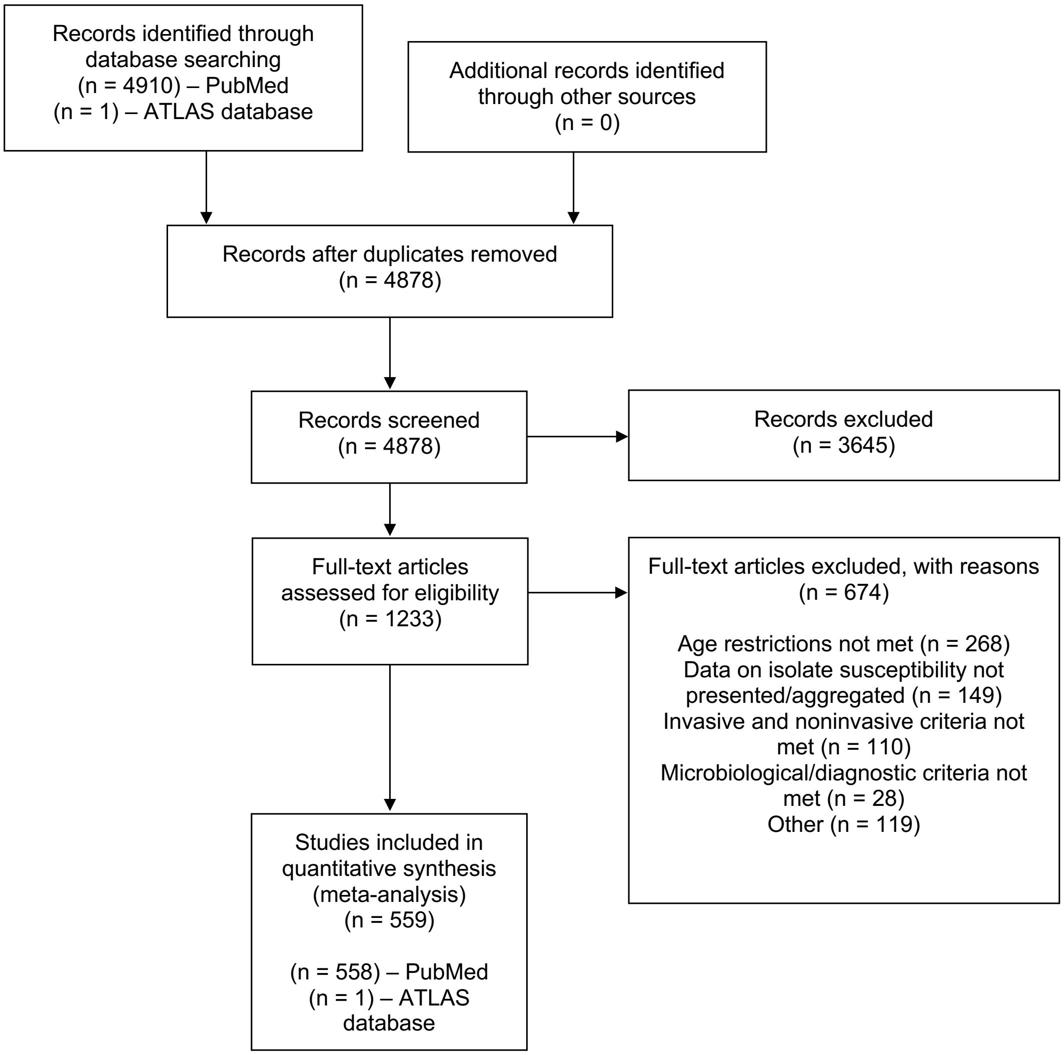

Figure S1: PRISMA flowchart. We illustrate studies included and excluded through literature the search and screening process. We identified 4910 records by searching the PubMed database for articles published between January 1, 2000 and November 24, 2020, and further included data from the Pfizer ATLAS global surveillance database. We detail reasons for exclusion of studies in the supplementary information (**Supplement Page 9).**

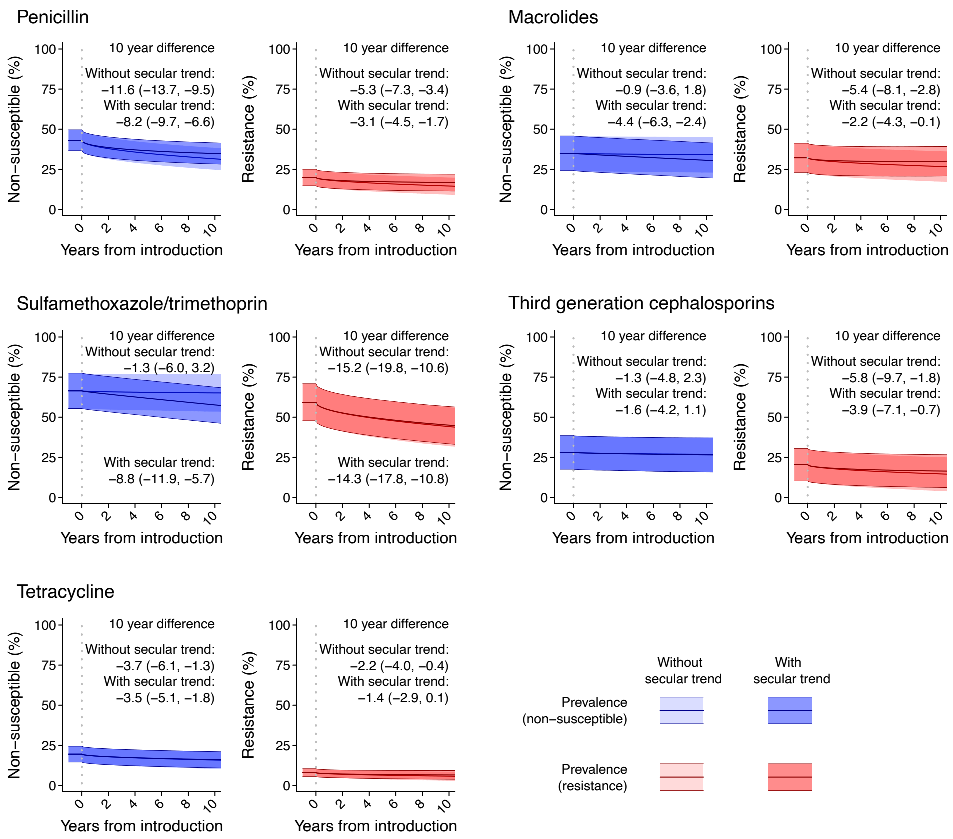

Figure S2. Post-vaccination changes in susceptibility to various drug classes, in analyses including all isolates. Plots illustrate changes in the proportion of isolates showing nonsusceptibility or resistance to each drug class over a 10-year period following PCV implementation, for all data included in the study. Estimates are adjusted for years passing since vaccine implementation, calendar time (to account for secular trends independent of time since vaccine implementation), invasive or non-invasive isolate source, and country wealth (expressed in log per-capita gross domestic product [GDP], normalized to 2010 US dollars); random effects are specified for each Global Burden of Disease region and for each study. Plotted estimates correspond to expected changes in susceptibility among non-invasive pneumococcal isolates sampled from children in a hypothetical country with per-capita GDP equal to $22,000. Shaded areas denote 95% confidence intervals around median point estimates (center lines); each panel also indicates the median estimated difference (and 95% confidence interval) in the absolute proportion of isolates showing nonsusceptibility or resistance to each drug class over a 10-year period following PCV implementation without secular trends. Analyses make use of data from all isolates summarized in **Table 1, Table 2**, and **Table S3.**

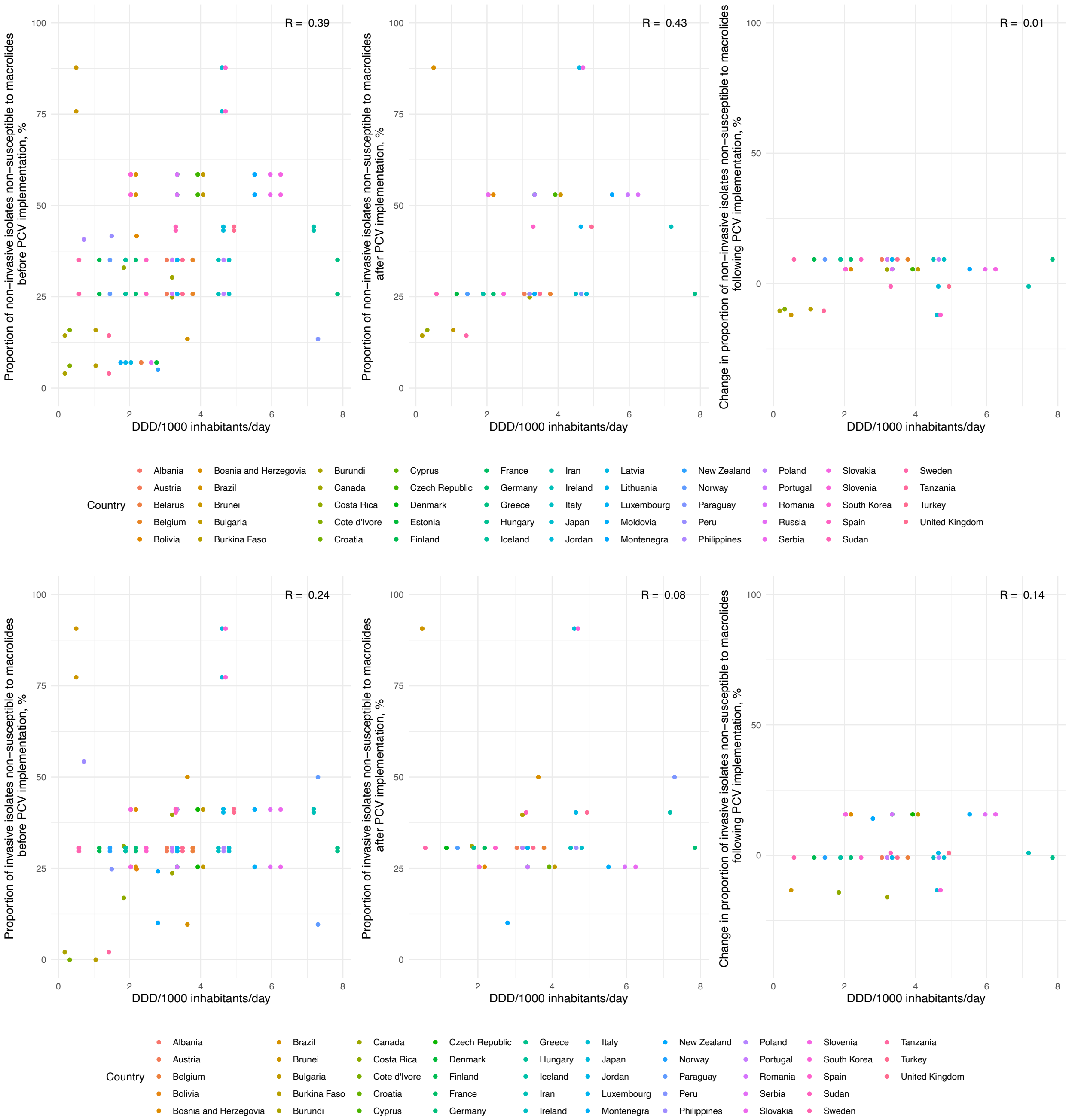

Figure S3 (part A). Changes in susceptibility to macrolides by country specific rates of consumption. Plots illustrate drug consumption by country (in DDD/person-years, sourced from The WHO Report on Surveillance of Antibiotic Consumption) on the x axis plotted against (1) the pre- (2) post-PCV proportion of nonsusceptible isolates by region and (3) the change in proportion of isolates that were nonsusceptible. The proportion of isolates nonsusceptible (y axis) are measured at the GBD regional level and were available from the systematic review. Each point estimate corresponds to countries within GBD regions for which antibiotic consumption data were available from the WHO Report on Surveillance of Antibiotic Consumption. Correlation coefficients were calculated using Spearman's Rank Correlation coefficient.

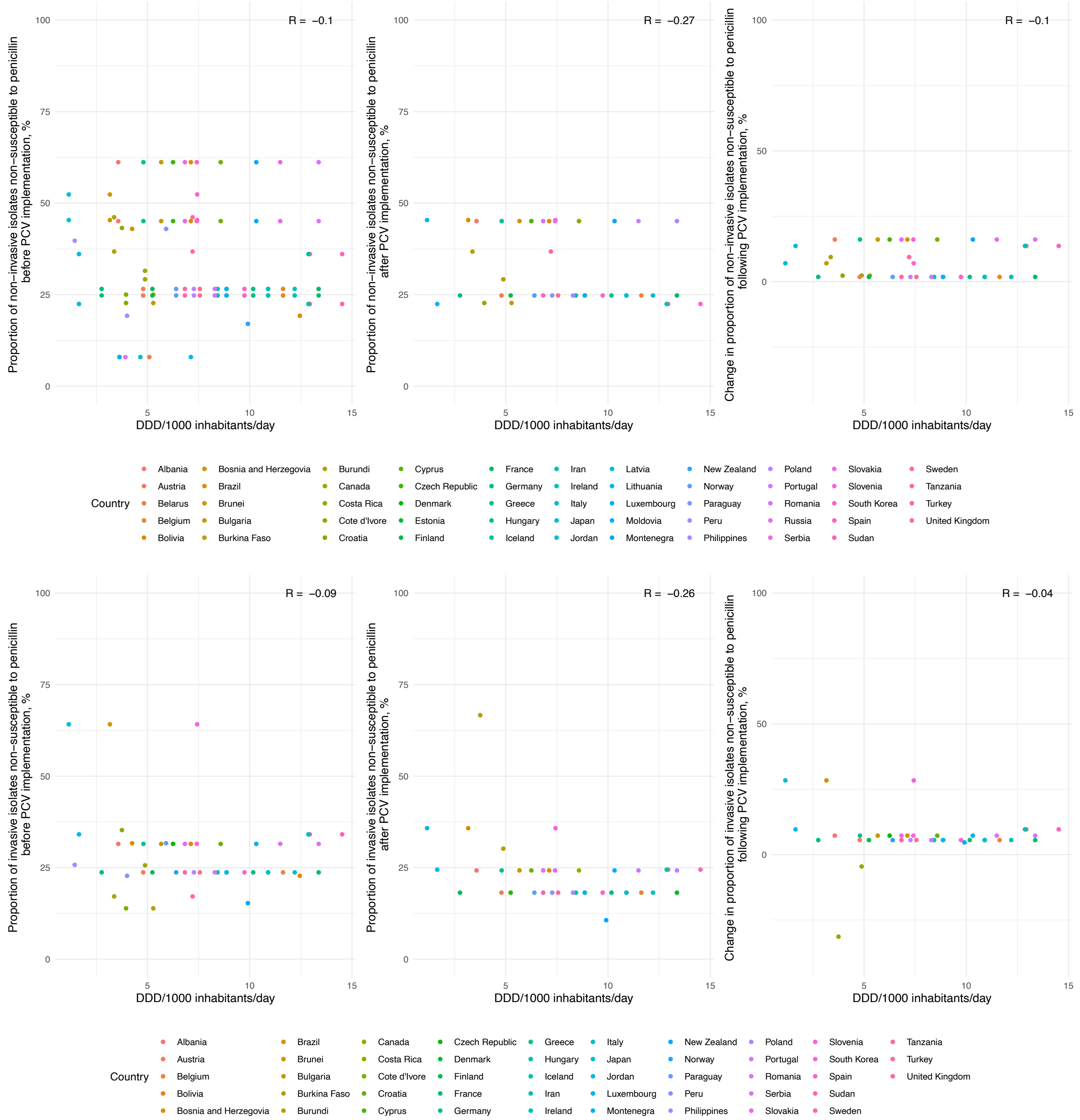

Figure S3 (part B). Changes in susceptibility to penicillin by country specific rates of consumption. Plots illustrate drug consumption by country (in DDD/person-years, sourced from The WHO Report on Surveillance of Antibiotic Consumption) on the x axis plotted against (1) the pre- (2) post-PCV proportion of nonsusceptible isolates by region and (3) the change in proportion of isolates that were nonsusceptible. The proportion of isolates nonsusceptible (y axis) are measured at the GBD regional level and were available from the systematic review. Each point estimate corresponds to countries within GBD regions for which antibiotic consumption data were available from the WHO Report on Surveillance of Antibiotic Consumption. Correlation coefficients were calculated using Spearman's Rank Correlation coefficient.

Table S1: Susceptibility breakpoints.

Drug	Classification	Measurement	
		MIC[1] (µg/mL)	Zone diameter (mm)
Penicillin (Benzylpenicillin)			(oxacillin disk)
	Susceptible	≤0.06	≥20
	Intermediate	0.12-2	11-19
	Resistant	>2	≤10
Macrolides (erythromycin, clarithromycin, azithromycin)			
	Susceptible	≤0.25	≥22
	Intermediate	0.5	20-21
	Resistant	>0.5	≤19
Third generation cephalosporins			
Cefotaxime			
	Susceptible	≤0.5	– –
	Intermediate	1-2	– –
	Resistant	>2	– –
Cefpodoxime			
	Susceptible	≤0.25	– –
	Intermediate	0.5	– –
	Resistant	>0.5	– –
Ceftriaxone			
	Susceptible	≤0.5	– –
	Intermediate	1-2	– –
	Resistant	>2	– –
Tetracycline (tetracycline, doxycycline)			
	Susceptible	≤1	≥20
	Intermediate	2	– –
	Resistant	>2	≤19
Sulfamethoxazole/trimethoprim			
	Susceptible	≤1	≥17
	Intermediate	2	– –
	Resistant	>2	≤16

Values in the table indicate ranges used to categorize isolates as susceptible, intermediate, or resistant to each drug. Determinations of susceptibility to third-generation cephalosporins were based on the first drug for which data were presented in the original source. Breakpoints presented above are consistent under CLSI and EUCAST standards.

[1]Minimum inhibitory concentration

Table S2: Regional designations.

Super regions	Regions	Countries	GDP/capita, 2000-2018 (range, 2010 USD)
High income			
	Southern Latin America		
		Argentina	8224-10043
		Childe	9420-5130
		Uruguay	9002-14617
	Western Europe		
		Andorra	40801-44569
		Austria	42001-50019
		Belgium	39588-47166
		Cyprus	26688-30926
		Denmark	55850-53873
		Finland	40403-48749
		France	38309-43663
		Germany	37930-47477
		Greece	23275-23558
		Greenland	32193-49310
		Iceland	37465-52103
		Ireland	44101-76880
		Israel	27741-34745
		Italy	36329-35431
		Luxembourg	93462-110742
		Malta	18323-28594
		Netherlands	46435-55022
		Norway	81653-92077
		Portugal	21497-23994
		Spain	28408-32949
		Sweden	44694-57921
		Switzerland	67807-79214
		United Kingdom	35672-43324
	Australasia		
		Australia	44334-50019
		New Zealand	29374-37997
	High income North America		
		Canada	39340-51391
		United States	44726-54759
	High income Asia-Pacific		
		Brunei	35931-31436
		Japan	42169-48919
		Singapore	33850-58247
		South Korea	15104-26761
Latin America and Caribbean			
	Caribbean		
		Antigua and Barbuda	13184-15143
		The Bahamas	31570-27261
		Barbados	15569-16018
		Belize	3783-4248
		Bermuda	79988-79251
		Cuba	3480-6816
		Dominica	5578-6694
		Dominican Republic	4051-7698
		Grenada	6285-9096
		Guyana	2403-3992
		Haiti	755-730
		Jamaica	4658-4855
		Puerto Rico	24161-27341
		Saint Lucia	7347-8485
		Saint Vincent and the Grenadines	4753-6852
		Suriname	5726-8040
		Trinidad and Tobago	9785-15161
	Central Latin America		
		Colombia	4857-7691
		Costa Rica	6172-9889
		El Salvador	2666-3511
		Guatemala	2555-3160

		Honduras	1607-2219
		Mexico	9253-10403
		Nicaragua	1294-1860
		Panama	5493-11723
		Venezuela	11944-7697
	Tropical Latin America		
		Brazil	8803-11026
		Paraguay	3570-5396
	Andean Latin America		
Sub-Saharan Africa		Bolivia	1601-2559
		Ecuador	3664-4185
		Peru	3242-6454
	Southern sub-Saharan Africa		
		Botswana	5211-8031
		Lesotho	809-1411
		Namibia	4012-6044
		South Africa	5938-7434
		Swaziland	3117-4774
		Zimbabwe	
	Western sub-Saharan Africa		
		Benin	694-897
		Burkina Faso	435-712
		Cameroon	1145-1500
		Cape Verde	2215-3759
		Chad	461-813
		Cote d'Ivoire	1355-1692
		The Gambia	832-786
		Ghana	952-1807
		Guinea	612-896
		Guinea-Bissau	549-622
		Liberia	614-541
		Mali	553-778
		Mauritania	1026-1334
		Niger	322-403
		Nigeria	1383-2396
		Sao Tome and Principe	844-1297
		Senegal	1114-1546
		Sierra Leone	302-407
		Togo	530-676
	Central sub-Saharan Africa		
		Angola	2195-3229
		Central African Republic	422-378
		Republic of Congo	2438-2651
		Democratic Republic of the Congo	289-418
		Equatorial Guinea	5558-10255
		Gabon	10160-9042
	Eastern sub-Saharan Africa		
		Burundi	229-210
		Comoros	1294-1041
		Djibouti	
		Eritrea	845-715
		Ethiopia	197-570
		Kenya	819-1202
		Madagascar	492-490
		Malawi	392-517
		Mozambique	289-591
		Rwanda	330-824
		Somalia	NA
		South Sudan	1508-730
		Tanzania	522-957
		Uganda	418-710
		Zambia	948-1672
North Africa and Middle East			
		Afghanistan	330-563
		Algeria	3557-4764

		Bahrain	22955-21438
		Egypt	1980-2907
		Iran	4818-6952
		Iraq	4323-5477
		Jordan	2838-3266
		Kuwait	36068- 33112
		Lebanon	5716-6249
		Libya	8963-7536
		Morocco	1976-3357
		Palestine	
		Oman	18698-15796
		Qatar	60837-63260
		Saudi Arabia	18352-20819
		Sudan	1017-1855
		Syria	
		Tunisia	3001-4401
		Turkey	8237-15068
		United Arab Emirates	63251-40782
		Yemen	1168-667
South Asia			
		Bangladesh	524-1203
		Bhutan	1165-3172
		India	826-2100
		Nepal	455-817
		Pakistan	824-1197
Southeast, Asia, East Asia, Oceania			
	East Asia		
		China	1767-7752
		North Korea	NA
		Taiwan	NA
	Southeast Asia		
		Cambodia	431-1205
		Indonesia	2143-4284
		Lao People's Democratic Republic	672-1785
		Malaysia	7007-12120
		Maldives	5797-8033
		Mauritius	4664-10578
		Myanmar	341-1571
		Philippines	1607-3021
		Seychelles	9790-14385
		Sri Lanka	1825-3936
		Thailand	3458-6361
		Timor-Leste	1325-2759
		Vietnam	765-1964
	Oceania		
		American Samoa	10551-9271
		Federated States of Micronesia	2707-2728
		Fiji	3315-4795
		Guam	27723-31477
		Kiribati	1722-1762
		Marshall Islands	2696-3066
		Papua New Guinea	1643-2416
		Samoa	2855-3748
		Solomon Islands	1252-1482
		Tonga	3279-4054
		Vanuatu	2841-2875
Central Europe, Eastern Europe, Central Asia			
	Central Asia		
		Armenia	1404-4406
		Azerbaijan	1658-5768
		Georgia	1633-4721
		Kazakhstan	4491-11165
		Kyrgyzstan	654-1087
		Mongolia	1600-4210
		Tajikistan	415-1073
		Turkmenistan	2381-7647
		Uzbekistan	979-2366

	Central Europe		
		Albania	2244-5079
		Bosnia and Herzegovina	3018-6032
		Croatia	10458-15889
		Czech Republic	14806-23358
		Hungary	10465-16647
		Macedonia	3448-5394
		Montenegro	4950-8244
		Poland	8526-16659
		Romania	4896-11532
		Serbia	3405-6886
		Slovakia	10320-20599
		Slovenia	18523-26768
	Eastern Europe		
		Belarus	2879-6744
		Estonia	10056-19954
		Latvia	6959-16268
		Lithuania	6926-17708
		Republic of Moldova	1162-2668
		Russian Federation	6591-11729
		Ukraine	1817-3110

Table S3: Total number of studies included in systematic review by region.

Region[1]		Penicillin		Macrolides		Sulfamethoxazole/ trimethoprim		Third-generation cephalosporins		Tetracycline	
		NS	RES	NS	RES	NS	RES	NS	RES	NS	RES
High Income											
	Southern Latin America	9	8	2	4	--	2	1	2	1	1
	Western Europe	114	69	68	66	27	22	42	30	29	29
	High Income North America	49	29	19	12	18	9	20	9	7	8
	Australasia	15	9	7	5	2	2	5	4	4	3
	High Income Asia Pacific	31	26	17	15	10	7	16	11	10	10
	**All high income**	213	135	108	96	56	42	80	52	47	47
Latin America & Caribbean											
	Caribbean	3	4	2	3	--	2	2	1	1	2
	Central Latin America	14	13	11	8	6	6	7	5	3	1
	Tropical Latin America	22	19	8	6	6	6	7	3	4	5
	Andean Latin America	7	5	6	5	5	4	3	3	2	2
	**All Latin America & Caribbean**	42	37	25	20	17	18	17	10	8	8
Sub-Saharan Africa											
	Southern sub-Saharan Africa	9	4	5	3	4	2	2	1	2	3
	Western sub-Saharan Africa	14	12	9	10	9	11	10	8	9	10
	Central sub-Saharan Africa	4	2	3	3	3	3	1	1	3	3
	Eastern sub-Saharan Africa	30	24	24	21	21	23	13	11	13	15
	**All sub-Saharan Africa**	54	41	38	36	35	39	25	20	26	30
North Africa & Middle East											
	**All North Africa & Middle East**	42	39	23	29	18	20	19	17	14	14
South Asia											
	**All South Asia**	21	14	14	15	19	19	13	10	7	5
Southeast Asia, East Asia & Oceania											
	East Asia	44	34	31	29	18	17	28	25	16	16
	Southeast Asia	20	14	13	14	10	11	15	13	10	11
	Oceania	3	2	2	2	2	2	2	2	1	1
	**All Southeast Asia, East Asia & Oceania**	65	48	44	43	29	29	43	38	25	26
Central Europe, Eastern Europe											
	Eastern Europe	8	6	7	6	5	6	2	2	4	4
	Central Europe	11	12	9	16	2	8	6	5	4	6
	**All Central Europe & Eastern Europe**	18	17	15	21	7	14	7	6	7	9

Entries indicate the number of distinct publications meeting inclusion criteria; the ATLAS Database is counted as one study throughout. Entries may not sum to the super-region total if a single study presented data from multiple countries.

[1]We indicate countries belonging to each Global Burden of Disease region and super-region in **Table S2**.

Table S4: Summary of available studies for comparisons of isolate susceptibility ≥1 year before and ≥3 years after PCV implementation.

Super-region	Region	Country	PCV Intro Status	Number of Studies	Number of Isolates
High Income	Southern Latin America	Argentina	Pre PCV Intro	6	6102
		Argentina	3 years post PCV Intro	1	23
		Chile	Pre PCV Intro	6	11740
		Chile	3 years post PCV Intro	1	199
		Uruguay	Pre PCV Intro	4	1502
	Western Europe	Belgium	Pre PCV Intro	5	2170
		Belgium	3 years post PCV Intro	2	7203
		Cyprus	3 years post PCV Intro	1	1960
		Denmark	Pre PCV Intro	1	803
		Denmark	3 years post PCV Intro	1	77
		Finland	Pre PCV Intro	2	458
		Finland	3 years post PCV Intro	1	238
		France	Pre PCV Intro	9	9365
		France	3 years post PCV Intro	4	2454
		Germany	Pre PCV Intro	5	6738
		Germany	3 years post PCV Intro	2	4742
		Greece	Pre PCV Intro	12	21431
		Greece	3 years post PCV Intro	3	2505
		Iceland	Pre PCV Intro	7	21750
		Ireland	Pre PCV Intro	1	36
		Ireland	3 years post PCV Intro	1	28
		Israel	Pre PCV Intro	9	20582
		Israel	3 years post PCV Intro	2	1192
		Italy	Pre PCV Intro	6	1334
		Italy	3 years post PCV Intro	4	3963
		Malta	Pre PCV Intro	1	0
		Netherlands	3 years post PCV Intro	1	18
		Norway	Pre PCV Intro	1	136
		Norway	3 years post PCV Intro	1	1687
		Portugal	Pre PCV Intro	13	22564
		Spain	Pre PCV Intro	6	4246
		Spain	3 years post PCV Intro	5	3333
		Sweden	Pre PCV Intro	7	73505
		Sweden	3 years post PCV Intro	2	2422
		Switzerland	Pre PCV Intro	1	9
		Switzerland	3 years post PCV Intro	1	45
		United Kingdom	Pre PCV Intro	4	6083
		United Kingdom	3 years post PCV Intro	1	169
	High Income North America	Canada	Pre PCV Intro	6	6632
		Canada	3 years post PCV Intro	4	7409
		United States of America	Pre PCV Intro	18	12804
		United States of America	3 years post PCV Intro	24	42152
	Australasia	Australia	Pre PCV Intro	12	14182
		Australia	3 years post PCV Intro	3	391
		New Zealand	Pre PCV Intro	1	8528
	High Income Asia Pacific	Japan	Pre PCV Intro	12	3344
		Japan	3 years post PCV Intro	4	6562
		Singapore	Pre PCV Intro	4	2264

		South Korea	Pre PCV Intro	6	5518
		South Korea	3 years post PCV Intro	2	1783
Latin American & Carribean	Carribean	Cuba	Pre PCV Intro	1	970
		Dominican Republic	Pre PCV Intro	1	63
		Jamaica	Pre PCV Intro	2	144
		Trinidad and Tobago	Pre PCV Intro	1	160
	Central Latin America	Colombia	Pre PCV Intro	5	2972
		Colombia	3 years post PCV Intro	1	39
		Costa Rica	Pre PCV Intro	1	150
		Guatemala	Pre PCV Intro	2	1725
		Honduras	Pre PCV Intro	1	9
		Mexico	Pre PCV Intro	10	4387
		Mexico	3 years post PCV Intro	1	132
		Panama	Pre PCV Intro	1	18
		Venezuela	Pre PCV Intro	4	1150
	Tropical Latin America	Brazil	Pre PCV Intro	21	10797
		Brazil	3 years post PCV Intro	2	674
	Andean Latin America	Peru	Pre PCV Intro	4	2296
Sub-Saharan Africa	Southern sub-Saharan Africa	South Africa	Pre PCV Intro	7	20965
		South Africa	3 years post PCV Intro	3	2537
		Burkina Faso	Pre PCV Intro	1	1160
		Cameroon	Pre PCV Intro	1	120
	Western sub-Saharan Africa	Cote d Ivoire	Pre PCV Intro	1	966
		Gambia	3 years post PCV Intro	1	1338
		Ghana	Pre PCV Intro	5	2309
		Ghana	3 years post PCV Intro	3	1515
		Mali	Pre PCV Intro	1	106
		Nigeria	Pre PCV Intro	3	176
		Senegal	Pre PCV Intro	1	358
	Central sub-Saharan Africa	Angola	3 years post PCV Intro	2	1329
		Central African Republic	Pre PCV Intro	1	1734
	Eastern sub-Saharan Africa	Ethiopia	Pre PCV Intro	2	638
		Ethiopia	3 years post PCV Intro	5	2913
		Kenya	Pre PCV Intro	6	11098
		Kenya	3 years post PCV Intro	1	21
		Malawi	Pre PCV Intro	4	3171
		Mozambique	Pre PCV Intro	3	2346
		Tanzania	Pre PCV Intro	4	1979
		Uganda	Pre PCV Intro	6	1751
		Zambia	Pre PCV Intro	1	448
North Africa and Middle East	North Africa and Middle East	Algeria	Pre PCV Intro	3	423
		Egypt	Pre PCV Intro	5	2177
		Iran	Pre PCV Intro	7	3552
		Jordan	Pre PCV Intro	3	2865
		Kuwait	Pre PCV Intro	2	257
		Kuwait	3 years post PCV Intro	2	270
		Lebanon	Pre PCV Intro	1	303
		Morocco	Pre PCV Intro	3	1854
		Morocco	3 years post PCV Intro	2	206
		Oman	Pre PCV Intro	1	18

		Oman	3 years post PCV Intro	1	350
		Saudi Arabia	Pre PCV Intro	6	2831
		Saudi Arabia	3 years post PCV Intro	1	45
		Tunisia	Pre PCV Intro	2	555
		Turkey	Pre PCV Intro	11	4597
		Turkey	3 years post PCV Intro	6	1160
		Yemen	Pre PCV Intro	1	126
South Asia	South Asia	Bangladesh	Pre PCV Intro	5	6353
		India	Pre PCV Intro	16	18645
		Nepal	Pre PCV Intro	1	230
		Pakistan	Pre PCV Intro	2	106
		Pakistan	3 years post PCV Intro	1	32
Southeast/East Asia & Oceania	East Asia	China	Pre PCV Intro	52	41792
	Southeast Asia	Cambodia	Pre PCV Intro	1	250
		Indonesia	Pre PCV Intro	3	3566
		Malaysia	Pre PCV Intro	6	2958
		Mauritius	Pre PCV Intro	1	27
		Philippines	Pre PCV Intro	4	6197
		Philippines	3 years post PCV Intro	1	7
		Sri Lanka	Pre PCV Intro	2	1516
		Thailand	Pre PCV Intro	8	4393
		Vietnam	Pre PCV Intro	5	9118
	Oceania	Fiji	Pre PCV Intro	1	984
		New Caledonia	Pre PCV Intro	1	544
		Papua New Guinea	Pre PCV Intro	1	843
Central & Eastern Europe	Eastern Europe	Estonia	Pre PCV Intro	2	1027
		Latvia	Pre PCV Intro	1	32
		Lithuania	Pre PCV Intro	2	1303
		Russia	Pre PCV Intro	5	7631
		Russia	3 years post PCV Intro	2	1368
	Central Europe	Bosnia and Herzegovina	Pre PCV Intro	2	5234
		Bulgaria	Pre PCV Intro	2	537
		Croatia	Pre PCV Intro	2	553
		Czech Republic	Pre PCV Intro	2	1196
		Czech Republic	3 years post PCV Intro	1	48
		Hungary	Pre PCV Intro	2	79
		Hungary	3 years post PCV Intro	1	89
		Poland	Pre PCV Intro	5	6775
		Poland	3 years post PCV Intro	3	798
		Romania	Pre PCV Intro	3	1707
		Slovak Republic	Pre PCV Intro	1	18
		Slovenia	Pre PCV Intro	2	597
		Slovenia	3 years post PCV Intro	2	586

Table S5: Summary of PCV implementation status of the 104 countries included in the systematic review, sourced from ViewHub.

Country	Super Region	Region	Current Vaccine Introduction Status	Current Immunization Program Type	Vaccine Introduction Date	Current or Planned Formulation	Current Formulation Start Date	Product Switches	Date of product switch
Algeria	North Africa and Middle East	North Africa and Middle East	No Decision	None					
Angola	sub-Saharan Africa	Central sub-Saharan Africa	No Decision	None					
Argentina	High Income	Southern Latin America	Introduced into national immunization program	Universal	1/1/12	PCV13	1/1/12		
Australia	High Income	Australasia	Introduced into national immunization program	Universal	1/1/05	PCV13	1/1/11	PCV7 to PCV13	1/1/05
Austria	High Income	Western Europe	Introduced into national immunization program	Universal	1/1/02	PCV10 & PCV13	2/1/12	PCV7 to PCV10 & PCV13	1/1/12
Bangladesh	South Asia	South Asia	Introduced into national immunization program	Universal	3/21/15	PCV10	3/21/15		
Belgium	High Income	Western Europe	Introduced into national immunization program	Universal	1/1/06	PCV10	1/1/16	13 to 10	
Bosnia And Herzegovina	Central Europe, Eastern Europe, and Central Asia	Central Europe	No Decision	None					
Brazil	Latin America and Carribean	Tropical Latin America	Introduced into national immunization program	Universal	11/1/10	PCV10	11/1/10		
Bulgaria	Central Europe, Eastern Europe, and Central Asia	Central Europe	Introduced into national immunization program	Universal	6/1/10	PCV13	6/1/10		
Burkina Faso	sub-Saharan Africa	Western sub-Saharan Africa	Introduced into national immunization program	Universal	10/31/13	PCV13	10/31/13		
Cambodia	Southeast Asia, East Asia, Oceania	Southeast Asia	Introduced into national immunization program	Universal	1/14/15	PCV13	1/14/15		

Cameroon	sub-Saharan Africa	Western sub-Saharan Africa	Introduced into national immunization program	Universal	7/1/13	PCV13	7/1/13		
Canada	High Income	High Income North America	Introduced into national immunization program	Universal	1/1/02	PCV13	1/1/10	7 to 10/13 to 13	1/1/02
Central African Republic	Sub-Saharan Africa	Central sub-Saharan Africa	Introduced into national immunization program	Universal	6/30/11	PCV13	6/30/11		
Chile	High Income	Southern Latin America	Introduced into national immunization program	Universal	1/1/11	PCV13	1/1/17	10 to 13	1/1/12
China	Southeast Asia, East Asia, Oceania	East Asia	No Decision	None					
Colombia	Latin America and Carribean	Central Latin America	Introduced into national immunization program	Universal	9/1/11	PCV10	9/1/11		
Congo, The Democratic Republic Of The	Sub-Saharan Africa	Central sub-Saharan Africa	Introduced into national immunization program	Universal	1/1/12	PCV13	1/1/12		
Costa Rica	Latin America and Carribean	Central Latin America	Introduced into national immunization program	Universal	1/1/08	PCV13	Unknown		1/1/08
Cote d Ivoire	Sub-Saharan Africa	Western sub-Saharan Africa	Introduced into national immunization program	Universal	9/30/14	PCV13	9/30/14		
Croatia	Central Europe, Eastern Europe, and Central Asia	Central Europe	No Decision	None					
Cuba	Latin America and Carribean	Caribbean	Non-Gavi planning introduction	None					
Cyprus	High Income	Western Europe	Introduced into national immunization program	Universal	1/1/07	PCV10	Unknown	7 to 10	1/1/07
Czech Republic	Central Europe, Eastern Europe, and Central Asia	Central Europe	Introduced into national immunization program	Universal	1/1/10	PCV13	1/1/10	7 to 13	
Denmark	High Income	Western Europe	Introduced into national	Universal	10/1/07	PCV13	4/15/10		10/1/07

			immunization program						
Dominican Republic	Latin America and Carribean	Caribbean	Introduced into national immunization program	Universal	7/1/13	PCV13	7/1/13		
Egypt	North Africa and Middle East	North Africa and Middle East	No Decision	None					
Estonia	Central Europe, Eastern Europe, and Central Asia	Eastern Europe	Introduced into national immunization program	Risk	7/1/14	PCV10 & PCV13	7/1/14		
Ethiopia	Sub-Saharan Africa	Eastern sub-Saharan Africa	Introduced into national immunization program	Universal	10/15/11	PCV10	10/15/11		
Fiji	Southeast Asia, East Asia, Oceania	Oceania	Introduced into national immunization program	Universal	10/29/12	PCV10	10/29/12		
Finland	High Income	Western Europe	Introduced into national immunization program	Universal	9/1/10	PCV10	9/1/10		
France	High Income	Western Europe	Introduced into national immunization program	Universal	5/1/06	PCV13	6/1/10	7 to 13	5/1/06
Gambia	Sub-Saharan Africa	Western sub-Saharan Africa	Introduced into national immunization program	Universal	8/19/09	PCV13	4/1/11	7 to 13	8/19/09
Germany	High Income	Western Europe	Introduced into national immunization program	Universal	7/1/06	PCV10 & PCV13	4/1/09	7 to 10/13	7/1/06
Ghana	Sub-Saharan Africa	Western sub-Saharan Africa	Introduced into national immunization program	Universal	4/26/12	PCV13	4/26/12		
Greece	High Income	Western Europe	Introduced into national immunization program	Universal	1/1/06	PCV13	1/1/10		1/1/06
Guatemala	Latin America and Carribean	Central Latin America	Introduced into national immunization program	Universal	1/1/12	PCV13	1/1/12		

Honduras	Latin America and Carribean	Central Latin America	Introduced into national immunization program	Universal	4/1/11	PCV13		4/1/11		
Hungary	Central Europe, Eastern Europe, and Central Asia	Central Europe	Introduced into national immunization program	Universal	4/1/09	PCV13		1/1/10	7 to 13	4/1/09
Iceland	High Income	Western Europe	Introduced into national immunization program	Universal	4/1/11	PCV10		4/1/11	7 to 10	
India	South Asia	South Asia	Introduced into national immunization program	Phased	5/13/17	PCV13		5/13/17		
Indonesia	Southeast Asia, East Asia, Oceania	Southeast Asia	Introduced into national immunization program	Phased	3/22/18	PCV13		3/22/18		
Iran	North Africa and Middle East	North Africa and Middle East	No Decision	None						
Ireland	High Income	Western Europe	Introduced into national immunization program	Universal	9/1/08	PCV13		10/1/10	7 to 13	9/1/08
Israel	High Income	Western Europe	Introduced into national immunization program	Universal	7/1/09	PCV13		11/1/10	7 to 13	7/1/09
Italy	High Income	Western Europe	Introduced into national immunization program	Universal	5/1/05	PCV13		1/1/12	7 to 13	5/1/05
Jamaica	Latin America and Carribean	Caribbean	Introduced into national immunization program	Risk	7/1/10	PCV13		7/1/10		
Japan	High Income	High Income Asia Pacific	Introduced into national immunization program	Universal	1/1/11	PCV13		1/1/11	7 to 13	
Jordan	North Africa and Middle East	North Africa and Middle East	Non-Gavi planning introduction	None						1/1/11
Kenya	Sub-Saharan Africa	Eastern sub-Saharan Africa	Introduced into national immunization program	Universal	2/14/11	PCV10		2/14/11		

South Korea	High Income	High Income Asia Pacific	Introduced into national immunization program	Universal	5/1/14	PCV10 & PCV13	5/1/14		3/1/10
Kuwait	North Africa and Middle East	North Africa and Middle East	Introduced into national immunization program	Universal	1/1/07	PCV13	8/1/10	7 to 13	1/1/07
Latvia	Central Europe, Eastern Europe, and Central Asia	Eastern Europe	Introduced into national immunization program	Universal	1/1/10	PCV10	Unknown	7 to 10	1/1/10
Lebanon	North Africa and Middle East	North Africa and Middle East	Introduced into national immunization program	Universal	6/1/15	PCV13	6/1/15		
Lithuania	Central Europe, Eastern Europe, and Central Asia	Eastern Europe	Introduced into national immunization program	Universal	10/1/14	PCV10	10/1/14		
Malawi	Sub-Saharan Africa	Eastern sub-Saharan Africa	Introduced into national immunization program	Universal	11/12/11	PCV13	11/12/11		
Malaysia	Southeast Asia, East Asia, Oceania	Southeast Asia	No Decision	None					
Mali	Sub-Saharan Africa	Western sub-Saharan Africa	Introduced into national immunization program	Universal	3/15/11	PCV13	3/15/11		
Malta	High Income	Western Europe	Non-Gavi planning introduction	None					
Mauritius	Southeast Asia, East Asia, Oceania	Southeast Asia	Introduced into national immunization program	Universal	3/24/16	PCV13	3/24/16		
Mexico	Latin America and Carribean	Central Latin America	Introduced into national immunization program	Universal	3/1/08	PCV13	1/1/11	7 to 13	1/1/09
Morocco	North Africa and Middle East	North Africa and Middle East	Introduced into national immunization program	Universal	10/20/10	PCV10	10/20/10		
Mozambique	Sub-Saharan Africa	Eastern sub-Saharan Africa	Introduced into national immunization program	Universal	4/1/13	PCV13	1/1/16	10 to 13	4/1/13
New Caledonia	Southeast Asia, East Asia, Oceania	Oceania	Introduced into national	Universal	5/1/06	PCV13	6/1/10	7 to 13	5/1/06

			immunization program						
Nepal	South Asia	South Asia	Introduced into national immunization program	Universal	1/19/15	PCV10	1/19/15		
Netherlands	High Income	Western Europe	Introduced into national immunization program	Universal	6/1/06	PCV10	5/1/11	7 to 10	6/1/06
New Zealand	High Income	Australasia	Introduced into national immunization program	Universal	7/1/08	PCV13	7/1/11	7 to 10 to 13	1/1/11
Niger	Sub-Saharan Africa	Western sub-Saharan Africa	Introduced into national immunization program	Universal	8/5/14	PCV13	8/5/14		
Nigeria	Sub-Saharan Africa	Western sub-Saharan Africa	Introduced into national immunization program	Universal	12/22/14	PCV10	12/22/14		
Norway	High Income	Western Europe	Introduced into national immunization program	Universal	7/1/06	PCV13	1/1/11	7 to 13	7/1/06
Oman	North Africa and Middle East	North Africa and Middle East	Introduced into national immunization program	Universal	1/1/08	PCV13	1/1/08		
Pakistan	South Asia	South Asia	Introduced into national immunization program	Universal	10/9/12	PCV10	10/9/12		
Panama	Latin America and Carribean	Central Latin America	Introduced into national immunization program	Universal	1/1/10	PCV13	1/1/11	7 to 13	1/1/10
Papua New Guinea	Southeast Asia, East Asia, Oceania	Oceania	Introduced into national immunization program	Universal	11/12/13	PCV13	11/12/13		
Peru	Latin America and Carribean	Andrean Latin America	Introduced into national immunization program	Universal	1/1/09	PCV13	10/20/15	7 to 10 to 13	1/1/09
Philippines	Southeast Asia, East Asia, Oceania	Southeast Asia	Introduced into national immunization program	Phased	7/17/13	PCV10 & PCV13	7/17/13		

Poland	Central Europe, Eastern Europe, and Central Asia	Central Europe	Introduced into national immunization program	Universal	3/1/06	PCV13	1/1/11	7 to 13	3/1/06
Portugal	High Income	Western Europe	Introduced into national immunization program	Universal	7/1/15	PCV13	7/1/15		
Romania	Central Europe, Eastern Europe, and Central Asia	Central Europe	No Decision	None					
Russian Federation	Central Europe, Eastern Europe, and Central Asia	Eastern Europe	Introduced into national immunization program	Universal	3/1/14	PCV13	3/1/14		3/1/13
Saudi Arabia	North Africa and Middle East	North Africa and Middle East	Introduced into national immunization program	Universal	3/1/09	PCV13	1/1/13	7 to 13	3/1/09
Senegal	Sub-Saharan Africa	Western sub-Saharan Africa	Introduced into national immunization program	Universal	11/5/13	PCV13	11/5/13		
Singapore	High Income	High Income Asia Pacific	Introduced into national immunization program	Universal	11/1/09	PCV13	12/1/11	7 to 13	11/1/09
Slovakia	Central Europe, Eastern Europe, and Central Asia	Central Europe	Introduced into national immunization program	Universal	1/1/09	PCV10 & PCV13	1/1/11	7 to 10/13	1/1/09
Slovenia	Central Europe, Eastern Europe, and Central Asia	Central Europe	Introduced into national immunization program	Universal	1/1/05	PCV13	1/1/15	7 to 10/13 to 13	1/1/05
South Africa	Sub-Saharan Africa	Southern sub-Saharan Africa	Introduced into national immunization program	Universal	4/1/09	PCV13	5/1/11	7 to 13	4/1/09
Spain	High Income	Western Europe	Introduced into national immunization program	Regional	6/1/01	PCV13	1/1/10	7 to 13	6/1/01
Sri Lanka	Southeast Asia, East Asia, Oceania	Southeast Asia	No Decision	None					
Sweden	High Income	Western Europe	Introduced into national immunization program	Universal	1/1/09	PCV10	1/1/19	7 to 10/13 to 10	1/1/09

Switzerland	High Income	Western Europe	Introduced into national immunization program	Universal	1/1/06	PCV13	1/1/11	7 to 13	1/1/06
Tanzania, United Republic Of	Sub-Saharan Africa	Eastern sub-Saharan Africa	Introduced into national immunization program	Universal	12/6/12	PCV13	12/6/12		
Thailand	Southeast Asia, East Asia, Oceania	Southeast Asia	No Decision	None					
Trinidad And Tobago	Latin America and Carribean	Caribbean	Introduced into national immunization program	Universal	1/1/09	PCV13	1/1/15	10 to 13	
Tunisia	North Africa and Middle East	North Africa and Middle East	Introduced into national immunization program	Universal	4/1/19	PCV10	4/1/19		
Turkey	North Africa and Middle East	North Africa and Middle East	Introduced into national immunization program	Universal	11/1/08	PCV13	1/1/11	7 to 13	11/1/08
Uganda	Sub-Saharan Africa	Eastern sub-Saharan Africa	Introduced into national immunization program	Universal	4/27/13	PCV10	4/27/13		
United Kingdom	High Income	Western Europe	Introduced into national immunization program	Universal	9/1/06	PCV13	4/1/10	7 to 13	9/1/06
United States	High Income	High Income North America	Introduced into national immunization program	Universal	7/1/00	PCV13	1/1/10	7 to 13	7/1/00
Uruguay	High Income	Southern Latin America	Introduced into national immunization program	Universal	3/1/08	PCV13	3/1/10	7 to 13	3/1/08
Venezuela	Latin America and Carribean	Central Latin America	Introduced into national immunization program	Universal	7/1/14	PCV13	7/1/14		
Viet Nam	Southeast Asia, East Asia, Oceania	Southeast Asia	No Decision	None					
Yemen	North Africa and Middle East	North Africa and Middle East	Introduced into national immunization program	Universal	1/29/11	PCV13	1/29/11		

Zambia	Sub-Saharan Africa	Eastern sub-Saharan Africa	Introduced into national immunization program	Universal	5/10/13	PCV10	5/10/13

Table S6: Total non-susceptible and resistant isolates included, by drug class and Global Burden of Disease region, excluding ATLAS database.

Region		Penicillin		Macrolides		Sulfamethoxazole/ trimethoprim		Third-generation cephalosporins		Tetracycline	
		Non-susceptible[1] n (%)	Resistant[1] n (%)	Non-susceptible[1] n (%)	Resistant[1] n (%)	Non-susceptible[1] n (%)	Resistant[1] n (%)	Non-susceptible[1] n (%)	Resistant[1] n (%)	Non-susceptible[1] n (%)	Resistant[1] n (%)
High Income											
	Southern Latin America	3,558 (38)	1,521 (16)	50 (3)	134 (4)	--	929 (49)	--	54 (14)	--	--
	Western Europe	16,140 (12)	3,048 (8)	12,011 (23)	10,220 (20)	5,840 (26)	2,433 (18)	1,349 (5)	147 (1)	3,176 (15)	2,389 (16)
	High Income North America	5,086 (23)	1,953 (10)	2,627 (29)	1,619 (31)	2,789 (34)	1,306 (22)	1,852 (18)	200 (7)	584 (14)	579 (14)
	Australasia	1,197 (17)	345 (8)	696 (16)	562 (19)	1,226 (39)	1,174 (36)	270 (11)	94 (3)	346 (11)	342 (10)
	High Income Asia Pacific	2,775 (51)	1,451 (34)	3,106 (86)	1,943 (80)	1,201 (57)	445 (35)	1,007 (26)	139 (10)	1,481 (83)	1,425 (79)
	**All countries**	**28,756 (16)**	**8,318 (11)**	**18,490 (27)**	**14,478 (22)**	**11,056 (31)**	**6,287 (24)**	**4,478 (10)**	**634 (2)**	**5,587 (18)**	**4,735 (19)**
Latin America and Caribbean											
	Caribbean	45 (18)	54 (25)	0 (0)	91 (37)	--	93 (40)	0 (0)	--	--	12 (30)
	Central Latin America	1,104 (46)	219 (8)	408 (29)	362 (18)	486 (61)	747 (40)	105 (17)	19 (4)	39 (17)	--
	Tropical Latin America	1,440 (31)	183 (6)	147 (13)	114 (18)	815 (71)	331 (44)	45 (4)	12 (3)	109 (20)	52 (17)
	Andean Latin America	574 (28)	118 (9)	419 (28)	287 (21)	802 (60)	651 (54)	65 (8)	12 (1)	409 (50)	340 (41)
	**All countries**	**3,163 (34)**	**574 (8)**	**974 (24)**	**854 (20)**	**2,103 (64)**	**1,822 (45)**	**215 (8)**	**43 (2)**	**557 (35)**	**404 (34)**
Sub-Saharan Africa											
	Southern sub-Saharan Africa	5,229 (45)	539 (28)	185 (20)	103 (5)	462 (49)	95 (18)	338 (13)	--	50 (41)	219 (11)
	Western sub-Saharan Africa	357 (24)	202 (12)	67 (8)	151 (11)	414 (59)	759 (63)	20 (2)	32 (4)	519 (63)	690 (61)
	Central sub-Saharan Africa	293 (37)	30 (7)	63 (8)	33 (4)	443 (57)	418 (54)	61 (37)	20 (12)	323 (42)	253 (33)
	Eastern sub-Saharan Africa	1,573 (28)	301 (6)	345 (7)	283 (6)	3,612 (76)	3,158 (64)	11 (0)	4 (0)	918 (25)	672 (22)
	**All countries**	**7,452 (38)**	**1,072 (12)**	**660 (9)**	**570 (7)**	**4,931 (69)**	**4,430 (60)**	**430 (7)**	**56 (1)**	**1,810 (33)**	**1,834 (27)**

North Africa and Middle East											
	**All countries**	**1,318 (27)**	**1,788 (35)**	**622 (33)**	**935 (25)**	**651 (55)**	**1,264 (54)**	**185 (11)**	**63 (3)**	**507 (38)**	**689 (41)**
South Asia											
	**All countries**	**265 (7)**	**206 (8)**	**364 (19)**	**462 (11)**	**1,632 (73)**	**3403 (74)**	**32 (2)**	**18 (3)**	**327 (32)**	**825 (56)**
Southeast Asia, East Asia & Oceania											
	East Asia	4,367 (56)	1,908 (32)	3,929 (90)	4,575 (90)	1,586 (74)	1,909 (70)	984 (29)	884 (19)	1,192 (84)	1,306 (82)
	Southeast Asia	1,558 (32)	420 (16)	1,143 (44)	1,503 (37)	1,189 (53)	953 (28)	383 (14)	33 (1)	1,284 (57)	1,221 (35)
	Oceania	181 (19)	39 (9)	4 (1)	3 (1)	65 (15)	34 (8)	3 (1)	2 (1)	4 (4)	4 (4)
	**All countries**	**6,106 (45)**	**2,367 (26)**	**5,076 (70)**	**6,081 (64)**	**2,840 (59)**	**2,896 (44)**	**1,370 (21)**	**919 (12)**	**2,480 (66)**	**2,531 (49)**
Central Europe, Eastern Europe											
	Eastern Europe	491 (16)	499 (12)	206 (7)	464 (21)	647 (65)	1,007 (50)	23 (47)	0 (0)	129 (32)	154 (23)
	Central Europe	991 (50)	396 (17)	413 (31)	2684 (32)	171 (80)	690 (46)	57 (6)	0 (0)	136 (49)	456 (34)
	**All countries**	**1,482 (29)**	**895 (14)**	**619 (15)**	**3148 (29)**	**818 (67)**	**1,697 (48)**	**80 (8)**	**0 (0)**	**265 (39)**	**610 (30)**
**All regions**											
	**All countries**	**95,501 (20)**	**28,446 (13)**	**52,624 (28)**	**51,659 (25)**	**45,779 (42)**	**38,931 (38)**	**13,363 (11)**	**3,385 (4)**	**23,232 (26)**	**21,742 (26)**

Numbers entered in red text indicate cells with values that changed relative to **Table 2** due to exclusion of ATLAS database isolates.

Table S7: Stratified estimates of prevalence of non-susceptibility to penicillin, by region and isolate source.

Super region	Region	Period	Estimated prevalence (95% CI)	
			Invasive isolates	Non-invasive isolates
High income				
	Southern Latin America			
		≥1yr pre-implementation	37.9 (33, 42.9)	– –
		≥3yr post-implementation	50 (31.3, 65.7)	– –
	Western Europe			
		≥1yr pre-implementation	23.7 (16.8, 30.6)	26.6 (18.6, 34.5)
		≥3yr post-implementation	18.2 (5.6, 30.7)	24.8 (14.1, 35.4)
	High income North America			
		≥1yr pre-implementation	25.6 (18.4, 32.9)	31.5 (11.5, 51.5)
		≥3yr post-implementation	30.2 (23.5, 36.8)	29.2 (16.3, 42.1)
	Australasia			
		≥1yr pre-implementation	15.3 (11.5, 19.1)	17 (14.4, 19.8)
		≥3yr post-implementation	10.7 (1.2, 20)	– –
	High income Asia-Pacific			
		≥1yr pre-implementation	64.2 (43.8, 84.4)	52.4 (32.5, 72.2)
		≥3yr post-implementation	35.8 (32, 39.5)	45.4 (30, 60.6)
	All high income			
		≥1yr pre-implementation	29.3 (24, 34.5)	31.5 (24.1, 38.9)
		≥3yr post-implementation	25.7 (19.8, 31.5)	29 (21.3, 36.7)
Latin America and Caribbean				
	Caribbean			
		≥1yr pre-implementation	61.2 (28.1, 94.2)	0 (0, 1.3)
		≥3yr post-implementation	– –	– –
	Central Latin America			
		≥1yr pre-implementation	35.3 (23.9, 46.6)	43.2 (20.3, 66)
		≥3yr post-implementation	66.7 (43, 81.9)	– –
	Tropical Latin America			
		≥1yr pre-implementation	31.7 (18, 45.3)	43 (26.3, 59.6)
		≥3yr post-implementation	– –	– –
	Andean Latin America			
		≥1yr pre-implementation	22.8 (17, 28.6)	19.2 (11.7, 26.8)
		≥3yr post-implementation	– –	– –
	All Latin America and Caribbean			
		≥1yr pre-implementation	32 (22.6, 41.4)	38.3 (25.9, 50.6)
		≥3yr post-implementation	61.3 (42.2, 75.5)	– –
Sub-Saharan Africa				
	Southern sub-Saharan Africa			
		≥1yr pre-implementation	38.3 (2.3, 74.2)	45.5 (36.4, 53.9)
		≥3yr post-implementation	– –	18.1 (15.4, 20.7)
	Western sub-Saharan Africa			
		≥1yr pre-implementation	13.9 (0.2, 27.5)	25 (12.6, 37.3)
		≥3yr post-implementation	– –	22.7 (17.4, 28.4)
	Central sub-Saharan Africa			
		≥1yr pre-implementation	– –	10 (6.8, 13.7)
		≥3yr post-implementation	– –	40 (34.6, 45.2)
	Eastern sub-Saharan Africa			
		≥1yr pre-implementation	17.1 (9.9, 24.3)	46.2 (28.7, 63.5)

		≥3yr post-implementation	– –	36.8 (25.7, 47.9)
	All sub-Saharan Africa			
		≥1yr pre-implementation	21.2 (11.6, 30.8)	39 (26.5, 51.4)
		≥3yr post-implementation	20.6 (6.8, 34.4)	29.9 (21.5, 38.2)
North Africa and Middle East				
		≥1yr pre-implementation	34.1 (21, 47.2)	36.1 (21.2, 50.9)
		≥3yr post-implementation	24.5 (0, 53.1)	22.5 (8.1, 36.7)
South Asia				
		≥1yr pre-implementation	13.1 (4.3, 21.9)	11.9 (5.2, 18.5)
		≥3yr post-implementation	– –	– –
Southeast and East Asia and Oceania				
	East Asia			
		≥1yr pre-implementation	56.3 (45.7, 66.9)	46.9 (27.9, 65.9)
		≥3yr post-implementation	– –	– –
	Southeast Asia			
		≥1yr pre-implementation	25.8 (5.6, 45.9)	39.7 (22.1, 57.3)
		≥3yr post-implementation	– –	– –
	Oceania			
		≥1yr pre-implementation	21.5 (15.7, 27.6)	16.4 (6.9, 26)
		≥3yr post-implementation	– –	– –
	All Southeast and East Asia and Oceania			
		≥1yr pre-implementation	49.6 (39.4, 59.7)	42.3 (29.8, 54.8)
		≥3yr post-implementation	– –	– –
Central and Eastern Europe				
	Eastern Europe			
		≥1yr pre-implementation	– –	7.9 (2.7, 13.2)
		≥3yr post-implementation	– –	– –
	Central Europe			
		≥1yr pre-implementation	31.5 (8.3, 54.6)	61.2 (33.9, 88.4)
		≥3yr post-implementation	24.2 (11.1, 38.9)	45.1 (31.1, 57.8)
	All Central and Eastern Europe			
		≥1yr pre-implementation	31 (8.9, 53.2)	29.1 (9, 49)
		≥3yr post-implementation	23.5 (10.7, 37.9)	45.1 (31.1, 57.8)

Numerical estimates are plotted in **Figure 1**. Estimates are aggregated from strata with ≥20 isolates and pooled using random effects for individual studies. We use the Clopper-Pearson method to compute 95% confidence intervals for strata with only one study.

Table S8: Stratified estimates of prevalence of non-susceptibility to macrolides, by region and isolate source.

**Super region**	**Region**	**Period**	**Estimated prevalence (95% CI)**	
			Invasive isolates	Non-invasive isolates
High income				
	Southern Latin America			
		≥1yr pre-implementation	21.5 (0, 57)	– –
		≥3yr post-implementation	71.4 (55.4, 82.4)	– –
	Western Europe			
		≥1yr pre-implementation	29.7 (21.8, 37.6)	35.1 (23.8, 46.4)
		≥3yr post-implementation	30.6 (15.7, 45.4)	25.8 (15.2, 36.3)
	High income North America			
		≥1yr pre-implementation	23.7 (10.8, 36.6)	30.3 (0, 80.8)
		≥3yr post-implementation	39.7 (32.2, 47.2)	24.8 (20.4, 29.3)
	Australasia			
		≥1yr pre-implementation	24.2 (22.3, 26.1)	5 (3.5, 6.7)
		≥3yr post-implementation	10.1 (0.4, 19.7)	– –
	High income Asia-Pacific			
		≥1yr pre-implementation	77.3 (59.5, 95.1)	75.8 (63.2, 88.4)
		≥3yr post-implementation	90.7 (88.1, 93.2)	87.7 (82.7, 92.7)
	All high income			
		≥1yr pre-implementation	31.8 (23.5, 40)	42.7 (31.7, 53.8)
		≥3yr post-implementation	29.9 (27.3, 32.5)	27.3 (19, 35.6)
Latin America and Caribbean				
	Caribbean			
		≥1yr pre-implementation	0.0 (0.0, 5.0)	– –
		≥3yr post-implementation	– –	– –
	Central Latin America			
		≥1yr pre-implementation	16.9 (8.8, 25)	33 (22.8, 43.1)
		≥3yr post-implementation	31.1 (18.2, 44.3)	– –
	Tropical Latin America			
		≥1yr pre-implementation	9.6 (6, 13.3)	13.4 (0, 33.8)
		≥3yr post-implementation	50 (29.1, 67.2)	– –
	Andean Latin America			
		≥1yr pre-implementation	24.8 (18.8, 30.7)	41.6 (0, 92.8)
		≥3yr post-implementation	– –	– –
	All Latin America and Caribbean			
		≥1yr pre-implementation	13.5 (8, 19)	30.8 (19, 42.6)
		≥3yr post-implementation	37.7 (26.3, 48.7)	– –
Sub-Saharan Africa				
	Southern sub-Saharan Africa			
		≥1yr pre-implementation	42.9 (30.5, 54.4)	38.8 (30.1, 47.3)
		≥3yr post-implementation	– –	16.8 (14.3, 19.4)
	Western sub-Saharan Africa			
		≥1yr pre-implementation	0 (0, 7.7)	6.1 (0, 12.1)
		≥3yr post-implementation	– –	15.9 (11.3, 20.9)
	Central sub-Saharan Africa			
		≥1yr pre-implementation	– –	0 (0, 0.9)
		≥3yr post-implementation	– –	0.9 (0.2, 2.2)
	Eastern sub-Saharan Africa			
		≥1yr pre-implementation	2.1 (0, 4.6)	4 (1, 6.9)

		≥3yr post-implementation	– –	14.4 (8, 20.8)
	All sub-Saharan Africa			
		≥1yr pre-implementation	6.6 (0, 16.2)	6.4 (1.5, 11.4)
		≥3yr post-implementation	14.1 (0, 31.4)	13.3 (8.4, 18.3)
North Africa and Middle East				
		≥1yr pre-implementation	41.3 (16.9, 65.5)	43.1 (25.3, 60.9)
		≥3yr post-implementation	40.3 (25.5, 55.1)	44.2 (36.3, 52)
South Asia				
		≥1yr pre-implementation	28.5 (15.3, 41.7)	18.7 (3.5, 33.7)
		≥3yr post-implementation	– –	– –
Southeast and East Asia and Oceania				
	East Asia			
		≥1yr pre-implementation	92.6 (86, 99.1)	84.6 (78.2, 90.9)
		≥3yr post-implementation	– –	– –
	Southeast Asia			
		≥1yr pre-implementation	54.3 (30.3, 78.2)	40.7 (21.3, 59.9)
		≥3yr post-implementation	– –	– –
	Oceania			
		≥1yr pre-implementation	0.0 (0.0, 2.6)	1.6 (0.4, 3.5)
		≥3yr post-implementation	– –	– –
	All Southeast and East Asia and Oceania			
		≥1yr pre-implementation	83.2 (72.3, 94.1)	63.5 (49.4, 77.6)
		≥3yr post-implementation	– –	– –
Central and Eastern Europe				
	Eastern Europe			
		≥1yr pre-implementation	– –	7 (2.8, 11.2)
		≥3yr post-implementation	– –	– –
	Central Europe			
		≥1yr pre-implementation	41.1 (28.1, 54.2)	58.5 (30.3, 86.6)
		≥3yr post-implementation	25.4 (15.3, 36.2)	52.9 (38.5, 65.2)
	All Central and Eastern Europe			
		≥1yr pre-implementation	40.2 (29.5, 50.8)	21.9 (2.1, 41.6)
		≥3yr post-implementation	25 (15, 35.7)	52.9 (38.5, 65.2)

Numerical estimates are plotted in **Figure 2**. Estimates are aggregated from strata with ≥20 isolates and pooled using random effects for individual studies. We use the Clopper-Pearson method to compute 95% confidence intervals for strata with only one study.

Table S9: Meta-regression model parameters for analysis of pneumococcal isolates, restricted to corpus of studies distinguishing intermediate and resistant isolates (*N*=297).

Drug class	Outcome phenotype	Predictors		
		Calendar time (years)	GDP per capita (log)	Invasive source
Penicillin				
	Nonsusceptibility	0.51%<br>(0.33%, 0.58%)	0.52%<br>(–1.48%, 2.57%)	–17.0%<br>(–14.8%, –19.2%)
	Resistance	0.37%<br>(0.24%, 0.50%)	1.14%<br>(–0.30%, 2.55%)	–2.31%<br>(–3.84%, –0.69%)
Macrolides				
	Nonsusceptibility	–0.23%<br>(–0.44%, –0.03%)	–5.78%<br>(–8.30%, –3.28%)	–8.22%<br>(–11.75%, –4.71%)
	Resistance	–0.09 %<br>(–0.28%, 0.10%)	–4.65%<br>(–6.99%, –2.25%)	–6.14%<br>(–9.13%, –3.07%)
Sulfamethoxazole/trimethoprim				
	Nonsusceptibility	–0.33%<br>(–0.67%, 0.01%)	5.29%<br>(1.61%, 8.89%)	–11.45%<br>(–16.59%, –6.26%)
	Resistance	0.40%<br>(0.09%, 0.72%)	4.24%<br>(0.86%,7.55%)	–1.49%<br>(–6.71%, 3.96%)
Third-generation cephalosporins				
	Nonsusceptibility	0.16%<br>(–0.03%, 0.34%)	0.38%<br>(–1.33%, 2.11%)	–5.89%<br>(–8.53%, –3.28%)
	Resistance	0.06%<br>(–0.04%, 0.16%)	0.90%<br>(0.23%, 1.60%)	–0.18%<br>(–1.87%, 1.50%)
Tetracycline				
	Nonsusceptibility	0.16%<br>(–0.09%, 0.41%)	–11.09%<br>(–13.94%, –8.23%)	5.60%<br>(–3.58%, 14.81%)
	Resistance	0.12%<br>(–0.11%, 0.36%)	–11.30%<br>(–14.04, –8.58%)	3.67%<br>(–2.97%, 10.43%)

Estimates indicate the adjusted increase in prevalence of the outcome estimated per unit change in the covariate, estimated via a linear mixed effects model with random effect terms for each study and global burden of disease region and fixed effects for the years passing from PCV implementation (transformed with square root function), midpoint year of the sampling interval for each study (calendar time (years)), country's per-capita gross domestic product (GDP) at the midpoint year of each study (log transformed), and source of isolate (invasive vs. noninvasive); resulting estimates are plotted in **Figure 3**. This analysis was restricted to the corpus of studies (*N*=297) presenting whether isolates were both resistant and/or nonsusceptible. A description of the functional form of the model from R is provided in supplemental file "Meta-Regression Models".

Table S10: Total studies and isolates included in analyses of nonsusceptibility and resistance of vaccine-targeted and non-vaccine serotypes.

Serotype group	Nonsusceptibility (132 studies; 77,113 isolates)						Resistance (71 studies; 23,681 isolates)					
	Penicillin		Macrolides		Sulfamethoxazole/ trimethoprim		Penicillin		Macrolides		Sulfamethoxazole/ trimethoprim	
	Studies	Isolates	Studies	Isolates	Studies	Isolates	Studies	Isolates	Studies	Isolates	Studies	Isolates
PCV-targeted serotypes	23	2,567	15	783	11	731	13	1,108	11	1,025	8	864
Non-PCV targeted serotypes	125	71,583	52	16,824	33	10,858	58	16,701	32	11,357	20	6,653

PCV: pneumococcal conjugate vaccine

Table entries indicate the number of distinct studies and number of isolates available for meta-regression analyses of the prevalence of nonsusceptibility and resistance to the penicillin, macrolides, and sulfamethoxazole/trimethoprim stratified PCV and non-PCV targeted serotypes, defined using the serotypes included in the PCV product in use during sample collection for each study. If a PCV product switch occurred during the study, the PCV product after the product switch used to determine vaccine-type and non-vaccine serotype classifications, unless the product switch occurred in the last year of sample collection. Meta-regression analyses of the proportion of isolates resistant to penicillin, macrolides, and sulfamethoxazole were not undertaken due to limited data availability, nor was meta-regression analysis of the proportion of isolates nonsusceptible to sulfamethoxazole.

Table S11: Meta-regression model parameters for analysis of vaccine-targeted and non-vaccine serotype isolates.

**Drug class**	**Predictors**			
	Calendar time (years)	GDP per capita (log)	Invasive source	Vaccine type isolate[1]
Penicillin	0.42% (0.05%, 0.79%)	5.91% (2.01%, 9.70%)	–12.89% (–17.61%, –7.93%)	7.66% (4.47%, 10.83%)
Macrolides	–0.6% (–1.2%, 0.0%)	5.80% (–0.81%, 12.52%)	–7.79% (–19.09%, 3.51%)	11.55% (6.65%, 16.42%)

Estimates indicate the adjusted increase in prevalence of the outcome estimated per unit change in the covariate, estimated via a linear mixed model with random effect terms for each study and global burden of disease region and fixed effects for the years passing from PCV implementation (defined as the years of PCV use with a serotype included in the formula for vaccine-type serotypes, and years of PCV use without a serotype included in the formula for non-vaccine types serotypes), midpoint year of the sampling interval for each study (calendar time (years)), country's per-capita gross domestic product (GDP) at the midpoint year of each study (log transformed), source of isolate (invasive vs. noninvasive), and an interaction term of years passing from vaccine implementation and whether isolate was considered vaccine type or non-vaccine type. A description of the functional form of models from R is provided in supplemental file "Meta-Regression Models". We selected an optimal functional form for the relationship between time since vaccine introduction and prevalence of nonsusceptibility by comparing the Akaike Information Criterion from fitted models; resulting estimates are plotted in **Figure 3**.

[1]The vaccine type isolate intercept indicates the difference in prevalence of nonsusceptibility for vaccine type isolates, versus non-vaccine type isolates, where vaccine-targeted serotypes were defined the serotypes included in the formulation of the PCV product introduced at initiation of sample collection for a given study. If no PCV product was introduced at the initiation of sample collection for a given study, all serotypes were considered non-vaccine type. If a PCV product switch occurred during the study, the PCV product after the product switch used to determine vaccine-type and non-vaccine serotype classifications, unless the product switch occurred in the last year of sample collection, in which case the PCV product before the product switch was used.

Table S12: Meta-regression model parameters for analysis of pneumococcal isolates, for all studies.

Drug class	Outcome phenotype	Predictors		
		Calendar time (years)	GDP per capita (log)	Invasive source
Penicillin				
	Nonsusceptibility	0.34%<br>(0.21%, 0.47%)	1.4%<br>(–0.34%, 3.12%)	–13.67%<br>(–15.3%, –12.7%)
	Resistance	0.22%<br>(0.09%, 0.36%)	0.64%<br>(–0.85%, 2.07%)	–0.06%<br>(–1.51%, 1.37%)
Macrolides				
	Nonsusceptibility	–0.35%<br>(–0.53%, –0.17%)	–1.97%<br>(–4.31%, 0.35%)	–11.08%<br>(–13.69%, –8.44%)
	Resistance	0.32 %<br>(0.15%, 0.50%)	–0.36%<br>(–2.55%, 1.84%)	–3.14%<br>(–5.53%, –0.70%)
Sulfamethoxazole/trimethoprim				
	Nonsusceptibility	–0.75%<br>(–1.06%, –0.43%)	3.79%<br>(0.23%, 7.27%)	–6.99%<br>(–10.91%, –3.01%)
	Resistance	0.09%<br>(–0.20%, 0.39%)	7.82%<br>(4.54%, 11.04%)	–1.98%<br>(–6.88%, 3.12%)
Third-generation cephalosporins				
	Nonsusceptibility	0.02%<br>(–0.15%, 0.19%)	0.96%<br>(–0.72%, 2.64%)	–6.42%<br>(–8.39%, –4.46%)
	Resistance	0.08%<br>(–0.03%, 0.19%)	0.88%<br>(0.15%, 1.63%)	–0.25%<br>(–2.04%, 1.54%)
Tetracycline				
	Nonsusceptibility	–0.03%<br>(–0.26%, 0.19%)	–10.75%<br>(–13.47%, –8.02%)	–1.65%<br>(–6.38%, 3.12%)
	Resistance	0.18%<br>(–0.03%, 0.41%)	–11.15%<br>(–13.84, –8.49%)	2.24%<br>(–3.39%, 8.17%)

Estimates indicate the adjusted increase in prevalence of the outcome estimated per unit change in the covariate, estimated via a linear mixed effects model with random effect terms for each study and global burden of disease region and fixed effects for the years passing from PCV implementation (transformed with square root function), midpoint year of the sampling interval for each study (calendar time (years)), country's per-capita gross domestic product (GDP) at the midpoint year of each study (log transformed), and source of isolate (invasive vs. noninvasive); resulting estimates are plotted in **Figure S2**. A description of the functional form of the model from R is provided in supplemental file "Meta-Regression Models".

Table S13: PRISMA Checklist.

Section/topic	#	Checklist item	Reported on page #
**TITLE**			
Title	1	Identify the report as a systematic review, meta-analysis, or both.	1
**ABSTRACT**			
Structured summary	2	Provide a structured summary including, as applicable: background; objectives; data sources; study eligibility criteria, participants, and interventions; study appraisal and synthesis methods; results; limitations; conclusions and implications of key findings; systematic review registration number.	2
**INTRODUCTION**			
Rationale	3	Describe the rationale for the review in the context of what is already known.	3
Objectives	4	Provide an explicit statement of questions being addressed with reference to participants, interventions, comparisons, outcomes, and study design (PICOS).	3
**METHODS**			
Protocol and registration	5	Indicate if a review protocol exists, if and where it can be accessed (e.g., Web address), and, if available, provide registration information including registration number.	3 (not registered)
Eligibility criteria	6	Specify study characteristics (e.g., PICOS, length of follow-up) and report characteristics (e.g., years considered, language, publication status) used as criteria for eligibility, giving rationale.	4
Information sources	7	Describe all information sources (e.g., databases with dates of coverage, contact with study authors to identify additional studies) in the search and date last searched.	3
Search	8	Present full electronic search strategy for at least one database, including any limits used, such that it could be repeated.	S3-S8
Study selection	9	State the process for selecting studies (i.e., screening, eligibility, included in systematic review, and, if applicable, included in the meta-analysis).	4
Data collection process	10	Describe method of data extraction from reports (e.g., piloted forms, independently, in duplicate) and any processes for obtaining and confirming data from investigators.	4-5
Data items	11	List and define all variables for which data were sought (e.g., PICOS, funding sources) and any assumptions and simplifications made.	4
Risk of bias in individual studies	12	Describe methods used for assessing risk of bias of individual studies (including specification of whether this was done at the study or outcome level), and how this information is to be used in any data synthesis.	5
Summary measures	13	State the principal summary measures (e.g., risk ratio, difference in means).	5
Synthesis of results	14	Describe the methods of handling data and combining results of studies, if done, including measures of consistency (e.g., I^2) for each meta-analysis.	5

Section/topic	#	Checklist item	Reported on page #
Risk of bias across studies	15	Specify any assessment of risk of bias that may affect the cumulative evidence (e.g., publication bias, selective reporting within studies).	5
Additional analyses	16	Describe methods of additional analyses (e.g., sensitivity or subgroup analyses, meta-regression), if done, indicating which were pre-specified.	5-6
**RESULTS**			
Study selection	17	Give numbers of studies screened, assessed for eligibility, and included in the review, with reasons for exclusions at each stage, ideally with a flow diagram.	6, S11
Study characteristics	18	For each study, present characteristics for which data were extracted (e.g., study size, PICOS, follow-up period) and provide the citations.	S45
Risk of bias within studies	19	Present data on risk of bias of each study and, if available, any outcome level assessment (see item 12).	N/A
Results of individual studies	20	For all outcomes considered (benefits or harms), present, for each study: (a) simple summary data for each intervention group (b) effect estimates and confidence intervals, ideally with a forest plot.	6-7
Synthesis of results	21	Present results of each meta-analysis done, including confidence intervals and measures of consistency.	6-7
Risk of bias across studies	22	Present results of any assessment of risk of bias across studies (see Item 15).	N/A
Additional analysis	23	Give results of additional analyses, if done (e.g., sensitivity or subgroup analyses, meta-regression [see Item 16]).	6-7
**DISCUSSION**			
Summary of evidence	24	Summarize the main findings including the strength of evidence for each main outcome; consider their relevance to key groups (e.g., healthcare providers, users, and policy makers).	7-8
Limitations	25	Discuss limitations at study and outcome level (e.g., risk of bias), and at review-level (e.g., incomplete retrieval of identified research, reporting bias).	8
Conclusions	26	Provide a general interpretation of the results in the context of other evidence, and implications for future research.	7-8
**FUNDING**			
Funding	27	Describe sources of funding for the systematic review and other support (e.g., supply of data); role of funders for the systematic review.	6

Table S14. Summary of 558 Studies Included in Systematic Review & Meta-Analysis (excludes ATLAS database).

							Analysis	
**Author**	**Year of Publication**	**Country**	**Sample Collection Dates**	**Total Isolates**	**Pre-Post**		**Meta-Regression**	**Serotype**
**El-Kholy et al.** [1]	2020	Egypt	2015-2017	110		X	X	
**Al-Lahham et al.**[2]	2020	Jordan	2017-2018	721		X	X	X
**Vidanapathirana et al.**[3]	2020	Sri Lanka	2017-2018	143		X	X	
**Udden et al.**[4]	2020	Angola	2017-2017	325		X	X	
**Ustundag et al.**[5]	2020	Turkey	2019-2019	9		X	X	
**Goh et al.**[6]	2020	Malaysia	2013-2015	3		X	X	X
**Tsai et al.**[7]	2020	China	2013-2017	25		X	X	X
**Zhao et al**[8]	2020	China	2016-2017	140		X	X	
**Wouters et al.**[9]	2020	Belgium	2016-2018	666		X	X	X
**Dai et al.**[10]	2020	Vietnam	2014-2015	234		X	X	
**Kawaguchiya et al.**[11]	2020	Japan	2018-2019	460		X	X	
**Vorobieva et al.**[12]	2020	Russia	2006-2006	168		X	X	X
**Hernstadt et al.**[13]	2020	Australia	2011-2017	83		X	X	
**Manenzhe et al.**[14]	2020	South Africa	2012-2013	149		X	X	
**Pelkonen et al.**[15]	2020	Angola	2016-2017	14		X	X	
**Lo et al.**[16]	2019	Multiple countries	1995-2015	898				X
**Mohammadi Gharibani et al.**[17]	2019	Iran	2015-2015	43		X	X	
**Kaplan et al.**[18]	2019		2014-2017	444		X	X	
**Danino et al.**[19]	2019	Israel	2009 - 2010	1068			X	X
**Nakano et al.**[20]	2019	Japan	2015 - 2017	497	X		X	
**Huang et al.**[21]	2019	China	2008 - 2017	449	X		X	
**Corcoran et al.**[22]	2019	Ireland	2007 - 2018	16			X	X
**Meropol et al.**[23]	2019	United States of America	2013 - 2014	38	X		X	
**Dilagui et al.**[24]	2019	Morocco	2017 - 2017	116	X		X	X
**Bao et al.**[25]	2019	China	2013 - 2017	94	X		X	
**Kourna Hama et al.**[26]	2019	Niger	2010 - 2016	25			X	
**Dayie et al.**[27]	2019	Ghana	2016 - 2016	288	X		X	
**Chen et al.**[28]	2019	China	2012 - 2016	470	X		X	
**Sutcliffe et al.**[29]	2019	India	2016 - 2017	272			X	X
**Shen et al.**[30]	2019	China	2015 - 2018	4	X		X	
**Takeuchi et al.**[31]	2019	Japan	2012 - 2015	18			X	
**Malaker et al.**[32]	2019	Bangladesh	2013 - 2015	33			X	
**Haile et al.**[33]	2019	Ethiopia	2018 - 2018	68	X		X	
**Wada et al.**[34]	2019	Ethiopia	2016 - 2016	311	X		X	
**Lee and Kuo**[35]	2019	China	2010 - 2016	46	X		X	
**Manenzhe et al.**[36]	2019	South Africa	2012 - 2014	661	X		X	X
**Kobayashi et al.**[37]	2019	Kenya	2013 - 2013	181			X	
**Mayanskiy et al.**[38]	2019	Russia	2013 - 2014	115			X	X

**Luna-Muschi et al.[39]**	2019	Peru	2006 - 2011	157		X	
**Negash et al.[40]**	2019	Ethiopia	2016 - 2017	1	X	X	
**Polkowska et al.[41]**	2019	Poland	2005 - 2015	110		X	X
**Shakur et al.[42]**	2019	Australia	2011 - 2014	12	X	X	
**Yan et al.[43]**	2019	China	2014 - 2016	51	X	X	
**Shi et al.[44]**	2019	China	2012 - 2017	111	X	X	
**Al-Jardani et al.[45]**	2019	Oman	2014 - 2016	35	X	X	
**Kavalari et al.[46]**	2019	Denmark	1999 - 2016	26			X
**Pourakbari et al.[47]**	2018	Iran	2011-2016	91	X	X	
**Yun et al.[48]**	2018	South Korea	1995 - 2013	183	X	X	
**Verani et al.[49]**	2018	Mozambique	2012 - 2013	343		X	
**Ubukata et al.[50]**	2018	Japan	2010 - 2010	300	X	X	
**Mulu et al.[51]**	2018	Ethiopia	2016 - 2017	31	X	X	
**Debess Magnussen et al.[52]**	2018	Iceland	2009 - 2011	271	X	X	
**Khan et al.[53]**	2018	Pakistan	2014 - 2015	38		X	
**Karlowsky et al.[54]**	2018	Canada	2011 - 2015	851	X	X	
**Chan et al.[55]**	2018	China	2013 - 2014	82	X	X	X
**Bojang et al.[56]**	2018	Gambia	2014 - 2015	235	X	X	
**Santana Hernández et al.[57]**	2018	Spain	2001 - 2016	190		X	
**Arushothy et al.[58]**	2018	Malaysia	2014 - 2017	245	X	X	
**Cai et al.[59]**	2018	China	2008 - 2017	123	X	X	
**Berger et al.[60]**	2018	Israel	2007 - 2015	252		X	
**Birindwa et al.[61]**	2018	Democratic Republic of Congo	2014 - 2015	163		X	
**Kastrin et al.[62]**	2018	Slovenia	1997 - 2017	992	X	X	
**Quirk et al.[63]**	2018	Iceland	2009 - 2017	3020		X	
**Ho et al.[64]**	2018	China	2015 - 2017	52			X
**Cassiolato et al.[65]**	2018	Brazil	2005 - 2017	260			X
**Rockett et al.[66]**	2018	Australia	2004 - 2014	112			X
**John et al.[67]**	2018	India	2007 - 2016	44			X
**Chen et al.[68]**	2018	China	2012 - 2015	20			X
**Zhang et al.[69]**	2017	China	2010 - 2015	80	X	X	
**Wang et al.[70]**	2017	China	2011 - 2014	284	X	X	
**Toledo et al.[71]**	2017	Cuba	2013 - 2013	194	X	X	
**Sihvonen et al.[72]**	2017	Finland	2009 - 2014	339	X	X	
**Sigurdsson et al.[73]**	2017	Iceland	2009 - 2015	1234		X	X
**Sampane-Donkor et al.[74]**	2017	Ghana	2015 - 2015	118	X	X	
**Raman et al.[75]**	2017	India	2012 - 2013	45	X	X	X
**Neves et al.[76]**	2017	Brazil	2014 - 2014	118	X	X	X
**Nagaraj et al.[77]**	2017	India	2011 - 2013	14	X	X	X
**Mayanskiy et al.[78]**	2017	Russia	2002 - 2013	49	X	X	X
**Manoharan et al.[79]**	2017	India	2011 - 2015	361	X	X	
**Lee et al.[80]**	2017	South Korea	2010 - 2015	267		X	X

**Kobayashi et al.[81]**	2017	Kenya	2009 - 2009	633	X	X	X
**Jiang et al.[82]**	2017	China	2012 - 2015	32	X	X	
**Houri et al.[83]**	2017	Iran	2013 - 2016	53	X	X	
**Gebre et al.[84]**	2017	Ethiopia	2014 - 2014	158	X	X	
**Gaviria-Agudelo et al.[85]**	2017	United States of America	1999 - 2014	738		X	
**Cho et al.[86]**	2017	China	2010 - 2015	498	X	X	
**Bloch et al.[87]**	2017	Tanzania	2014 - 2015	454		X	
**Arvas et al.[88]**	2017	Turkey	2014 - 2014	21	X	X	
**Ziane et al.[89]**	2016	Algeria	2010 - 2014	25	X	X	
**Wysocki et al.[90]**	2016	Poland	2008 - 2009	5		X	X
**Tomczyk et al.[91]**	2016	United States of America	2013 - 2013	243	X	X	
**Tantivitayakul et al.[92]**	2016	Thailand	2014 - 2014	77	X	X	
**Swedan et al.[93]**	2016	Jordan	2012 - 2013	53	X	X	
**Soysal et al.[94]**	2016	Turkey	2011 - 2013	133	X	X	X
**Soto-Noguerón et al.[95]**	2016	Mexico	2000 - 2007	37	X	X	
**Saha et al.[96]**	2016	Bangladesh	2007 - 2013	243	X	X	
**Nunes et al.[97]**	2016	Portugal	2009 - 2009	1092	X	X	
**Nor Azizah et al.[98]**	2016	Malaysia	2001 - 2011	28	X	X	
**Nhantumbo et al.[99]**	2016	Mozambique	2013 - 2014	17		X	X
**Moore et al.[100]**	2016	Cambodia	2007 - 2012	50	X	X	X
**Mokaddas et al.[101]**	2016	Kuwait	2003 - 2013	217	X	X	X
**Metcalf et al.[102]**	2016	United States of America	2008 - 2013	1425	X	X	
**Menezes et al.[103]**	2016	Brazil	2008 - 2009	398	X	X	X
**Lyu et al.[104]**	2016	China	2013 - 2014	21	X	X	
**Lindstrand et al.[105]**	2016	Sweden	2011 - 2015	907	X	X	
**Kwambana-Adams et al.[106]**	2016	Ghana	2015 - 2016	6	X	X	
**Kaur et al.[107]**	2016	United States of America	2006 - 2015	648	X	X	
**Janoir et al.[108]**	2016	France	2008 - 2012	328	X	X	X
**Hu et al.[109]**	2016	China	2009 - 2009	102	X	X	
**Horna et al.[110]**	2016	Peru	2007 - 2009	571		X	
**Hanke et al.[111]**	2016	Peru	2009 - 2011	250		X	X
**Hadjipanayis et al.[112]**	2016	Cyprus	2013 - 2014	280	X	X	X
**Hadinegoro et al.[113]**	2016	Indonesia	2012 - 2012	405	X	X	X
**Gopi et al.[114]**	2016	India	2010 - 2011	40	X	X	
**Gharailoo et al.[115]**	2016	Iran	2013 - 2014	42	X	X	
**Dunne et al.[116]**	2016	Australia	1999 - 2003	1051		X	X
**Choe et al.[117]**	2016	South Korea	2014 - 2014	222		X	X
**Chan et al.[118]**	2016	China	2013 - 2014	82	X	X	X
**Almazrou et al.[119]**	2016	Saudi Arabia	2007 - 2009	46		X	
**Zhou et al.[120]**	2016	China	2000 - 2014	201	X	X	X
**Miernyk et al.[121]**	2016	United States of America	2001 - 2013	99			X
**Nakano et al.[122]**	2015	Japan	2012 - 2014	629		X	

**Kang et al.**[123]	2015	China	2010 - 2013	83	X	X	
**Ceyhan et al.**[124]	2015	Turkey	2008 - 2014	335		X	
**Zhou et al.**[125]	2015	United States of America	2012 - 2013	240	X	X	
**Tóthpál et al.**[126]	2015	Hungary	2010 - 2012	358		X	
**Thummeepak et al.**[127]	2015	Thailand	2012 - 2013	38	X	X	
**Steens et al.**[128]	2015	Norway	2013 - 2013	580	X	X	
**Rutebemberwa et al.**[129]	2015	Uganda	2008 - 2008	89	X	X	
**Ramdani-Bouguessa et al.**[130]	2015	Algeria	2005 - 2012	97	X	X	
**Pan et al.**[131]	2015	China	2013 - 2013	284	X	X	X
**Olarte et al.**[132]	2015	United States of America	2007 - 2009	76	X	X	
**Mills et al.**[133]	2015	Ghana	2011 - 2011	207	X	X	
**Luthander et al.**[134]	2015	Sweden	2002 - 2007	68	X	X	
**Keenan et al.**[135]	2015	Ethiopia	2006 - 2007	82	X	X	
**Iregbu et al.**[136]	2015	Nigeria	2010 - 2013	10	X	X	
**Imöhl et al.**[137]	2015	Germany	1992 - 2013	3337	X	X	
**Imöhl et al.**[138]	2015	Germany	1992 - 2013	1317		X	
**Hsiao et al.**[139]	2015	China	2009 - 2013	55	X	X	
**Ho et al.**[140]	2015	China	2010 - 2013	156	X	X	X
**Greenhill et al.**[141]	2015	Papua New Guinea	1996 - 2005	177	X	X	X
**Gounder et al.**[142]	2015	United States of America	2000 - 2010	1344	X	X	X
**El-Nawawy et al.**[143]	2015	Egypt	2013 - 2014	100	X	X	X
**Dunais et al.**[144]	2015	France	1999 - 2012	1969	X	X	X
**Draz et al.**[145]	2015	Egypt	2009 - 2011	12	X	X	
**dos Santos et al.**[146]	2015	Brazil	1996 - 2012	496		X	
**Diawara et al.**[147]	2015	Morocco	2007 - 2014	136		X	
**Daana et al.**[148]	2015	Israel	2009 - 2011	829		X	
**Bles et al.**[149]	2015	Tanzania	2005 - 2009	104	X	X	X
**Balaji et al.**[150]	2015	India	2007 - 2013	114	X	X	X
**Ba et al.**[151]	2015	Senegal	2008 - 2013	102		X	
**Altun et al.**[152]	2015	Turkey	1996 - 2008	135		X	
**Ahl et al.**[153]	2015	Sweden	2000 - 2010	2364		X	X
**Tóthpál et al.**[154]	2015	Hungary	2009 - 2012	61			X
**Mijac et al.**[155]	2014	Bosnia and Herzegovina	2004 - 2009	5207	X	X	
**Deng et al.**[156]	2014	Canada	2007 - 2012	329	X	X	
**Dagan et al.**[157]	2014	Israel	2008 - 2010	5049		X	X
**Yadhav et al.**[158]	2014	India	2012 - 2014	3	X	X	
**von Mollendorf et al.**[159]	2014	South Africa	2003 - 2010	2631		X	
**von Gottberg et al.**[160]	2014	South Africa	2005 - 2008	3427	X	X	X
**Swann et al.**[161]	2014	Malawi	2002 - 2008	93	X	X	
**Safari et al.**[162]	2014	Indonesia	2012 - 2012	42	X	X	X
**Rupa et al.**[163]	2014	India	2009 - 2012	472	X	X	
**Phongsamart et al.**[164]	2014	Thailand	2009 - 2012	82	X	X	X

**Paulke-Korinek et al.[165]**	2014	Austria	2002 - 2012	205		X	
**Okade et al.[166]**	2014	Japan	2008 - 2012	564	X	X	X
**Mirzaei Ghazikalayeh et al.[167]**	2014	Iran	2011 - 2012	291	X	X	X
**Mayanskiy et al.[168]**	2014	Russia	2009 - 2013	835	X	X	X
**Luminos et al.[169]**	2014	Romania	2011 - 2013	564	X	X	X
**Tam et al.[170]**	2014	United States of America	2007 - 2012	253	X	X	
**Iroh Tam et al.[171]**	2014	United States of America	2007 - 2012	239	X	X	
**Gudnason et al.[172]**	2014	Iceland	2000 - 2003	3346	X	X	
**Cho et al.[173]**	2014	South Korea	2006 - 2010	123	X	X	
**Bayram et al.[174]**	2014	Turkey	2007 - 2013	43		X	
**Ba et al.[175]**	2014	Senegal	2007 - 2008	179	X	X	
**Al-Sheikh et al.[176]**	2014	Saudi Arabia	2009 - 2012	78		X	X
**Abdinia et al.[177]**	2014	Iran	2003 - 2013	37	X	X	
**Torres et al.[178]**	2013	Peru	2007-2009	572		X	
**Zuccoti et al.[179]**	2013	Italy	2011 - 2011	343	X	X	X
**Özdemir et al.[180]**	2013	Turkey	2011 - 2011	241	X	X	X
**Lee et al.[181]**	2013	United States of America	2000 - 2013	1496	X	X	
**Yatim et al.[182]**	2013	Malaysia	2010 - 2010	69	X	X	X
**Warda et al.[183]**	2013	Morocco	2007 - 2009	150	X	X	X
**von Gottberg et al.[184]**	2013	South Africa	2003 - 2008	6666	X	X	X
**Skovbjerg et al.[185]**	2013	Sweden	2004 - 2005	297	X	X	X
**Sharma et al.[186]**	2013	United States of America	1995 - 2009	481	X	X	X
**Parra et al.[187]**	2013	Colombia	2005 - 2011	336	X	X	
**Otsuka et al.[188]**	2013	Japan	2008 - 2011	551		X	X
**Niedzielski et al.[189]**	2013	Poland	2011 - 2011	51	X	X	X
**Neves et al.[190]**	2013	Brazil	2010 - 2010	121		X	X
**Nasereddin et al.[191]**	2013	Israel	2012 - 2013	213	X	X	X
**Musiime et al.[192]**	2013	Uganda	2007 - 2012	22	X	X	
**Morozumi et al.[193]**	2013	Japan	2008 - 2009	241	X	X	X
**Ma et al.[194]**	2013	China	2009 - 2012	87	X	X	
**Ma et al.[195]**	2013	China	2006 - 2008	171	X	X	X
**Liu et al.[196]**	2013	China	2009 - 2011	61	X	X	X
**Lee et al.[197]**	2013	South Korea	2006 - 2006	364	X	X	
**Lee et al.[198]**	2013	South Korea	1996 - 2008	74	X	X	
**Lee et al.[199]**	2013	China	2009 - 2012	42	X	X	
**Lee et al.[200]**	2013	South Korea	2010 - 2010	134	X	X	X
**Lalitha et al.[201]**	2013	India	2004 - 2004	776	X	X	
**Kumar et al.[202]**	2013	India	2009 - 2011	40	X	X	X
**Korona-Glowniak et al.[203]**	2013	Poland	2011 - 2011	66	X	X	X
**Kaplan et al.[204]**	2013	United States of America	2008 - 2011	702	X	X	
**Hare et al.[205]**	2013	United States of America	2004 - 2008	381	X	X	

**Githii et al.**[206]	2013	Kenya	2010 - 2010	55	X	X	
**dos Santos et al.**[207]	2013	Brazil	2010 - 2012	133		X	
**Dayie et al.**[208]	2013	Ghana	2011 - 2011	288	X	X	X
**Camilli et al.**[209]	2013	Italy	2011 - 2012	184	X	X	X
**Bautista-Márquez et al.**[210]	2013	Mexico	2010 - 2011	39		X	X
**Assefa et al.**[211]	2013	Ethiopia	2012 - 2012	96		X	
**Al-Waili et al.**[212]	2013	Saudi Arabia	2006 - 2012	408	X	X	
**van der Linden et al.**[213]	2013	Germany	1997 - 2012	204			X
**van der Linden et al.**[214]	2013	Germany	1997 - 2011	159			X
**Rolo et al.**[215]	2013	Spain	1997 - 2011	24			X
**Hulten et al.**[216]	2013	United States of America	1993 - 2011	594			X
**Sakata et al.**[217]	2013	Japan	2006 - 2011	76		X	
**Oftadeh et al.**[218]	2012	Australia	2002 - 2009	1170	X	X	X
**Wroe et al.**[219]	2012	United States of America	2001 - 2009	979		X	
**Tóthpál et al.**[220]	2012	Hungary	2009 - 2010	135		X	
**Shibl et al.**[221]	2012	Saudi Arabia	2005 - 2010	270	X	X	
**Riva et al.**[222]	2012	Italy	2008 - 2009	13	X	X	X
**Regev-Yochay et al.**[223]	2012	Israel	2009 - 2009	186		X	
**Porat et al.**[224]	2012	Israel	1999 - 2008	425	X	X	
**Nielsen et al.**[225]	2012	Ghana	2007 - 2009	22	X	X	
**Moyo et al.**[226]	2012	Tanzania	2010 - 2010	115	X	X	X
**Korona-Glowniak et al.**[227]	2012	Poland	2002 - 2003	356	X	X	
**Kateete et al.**[228]	2012	Uganda	2001 - 2002	38	X	X	
**Hernandez-Bou et al.**[229]	2012	Spain	2004 - 2006	116	X	X	
**Hanna-Wakim et al.**[230]	2012	Lebanon	2005 - 2011	101	X	X	X
**Grivea et al.**[231]	2012	Greece	2005 - 2009	1368	X	X	
**Gherardi et al.**[232]	2012	Italy	2006 - 2010	219		X	X
**Elmdaghri et al.**[233]	2012	Morocco	1994 - 2010	187	X	X	X
**El Mdaghri et al.**[234]	2012	Morocco	2007 - 2008	24	X	X	
**Devi et al.**[235]	2012	India	2009 - 2010	70	X	X	
**de Sevilla et al.**[236]	2012	Spain	2007 - 2009	120	X	X	
**Cho et al.**[237]	2012	South Korea	2009 - 2010	151	X	X	X
**Charfi et al.**[238]	2012	Tunisia	2000 - 2009	510	X	X	
**Benavides et al.**[239]	2012	Colombia	2006 - 2008	62	X	X	
**Barroso et al.**[240]	2012	Brazil	2000 - 2008	31	X	X	
**Arguedas et al.**[241]	2012	Costa Rica	2007 - 2009	21		X	X
**Andrade et al.**[242]	2012	Brazil	2007 - 2009	60	X	X	
**Stacevičienė et al.**[243]	2011	Poland	2002 - 2003	342	X	X	
**Tanaka et al.**[244]	2011	Japan	2008 - 2009	5	X	X	X
**Pourakbari et al.**[245]	2011	Iran	2001 - 2005	46	X	X	
**Hampton et al.**[246]	2011	United States of America	2008 - 2008	457	X	X	X
**Yoshioka et al.**[247]	2011	Brazil	2003 - 2008	107	X	X	X

**Vasoo et al.[248]**	2011	Singapore	1997 - 2008	161	X	X	
**Tóthpál et al.[249]**	2011	Hungary	2003 - 2010	58	X	X	
**Tarragó et al.[250]**	2011	Spain	2000 - 2008	471		X	X
**Sakata et al.[251]**	2011	Japan	2000 - 2010	82	X	X	
**Quintero et al.[252]**	2011	Venezuela	2007 - 2007	65	X	X	X
**Pimenta et al.[253]**	2011	Brazil	2005 - 2005	55	X	X	X
**Oishi et al.[254]**	2011	Japan	2007 - 2009	16	X	X	X
**Kuo et al.[255]**	2011	China	2005 - 2008	857	X	X	
**Korona-Glowniak et al.[256]**	2011	Poland	2002 - 2003	342	X	X	
**Kim et al.[257]**	2011	South Korea	2008 - 2008	94	X	X	X
**Kempf et al.[258]**	2011	France	2007 - 2007	1477		X	
**Kattan et al.[259]**	2011	Israel	2001 - 2010	120		X	X
**Jourdain et al.[260]**	2011	Belgium	2006 - 2008	362		X	X
**Janapatla et al.[261]**	2011	China	2008 - 2009	99	X	X	
**Ho et al.[262]**	2011	China	1999 - 2010	730	X	X	X
**Ho et al.[263]**	2011	China	1995 - 2009	187	X	X	
**Hashida et al.[264]**	2011	Japan	2004 - 2005	157	X	X	
**Grivea et al.[265]**	2011	Greece	2006 - 2009	813	X	X	X
**Grall et al.[266]**	2011	France	2009 - 2009	84	X	X	
**Falup-Pecurariu et al.[267]**	2011	Romania	2008 - 2009	162	X	X	X
**Ercan et al.[268]**	2011	Turkey	2007 - 2008	30		X	X
**Dunais et al.[269]**	2011	France	1999 - 2008	838	X	X	X
**Crump et al.[270]**	2011	Tanzania	2007 - 2008	6	X	X	
**Cornick et al.[271]**	2011	Malawi	2004 - 2006	176	X	X	
**Baş et al.[272]**	2011	Turkey	1998 - 2008	8		X	
**Alvares et al.[273]**	2011	Brazil	1999 - 2009	33	X	X	
**Al Ayed et al.[274]**	2011	Saudi Arabia	2001 - 2007	49	X	X	
**Yasin et al.[275]**	2011	Malaysia	2008 - 2009	12			X
**Picazo et al.[276]**	2011	Spain	2007 - 2009	53			X
**Miller et al.[277]**	2011	United Kingdom	2000 - 2010	211			X
**Bonofiglio et al.[278]**	2011	Argentina	1993 - 2002	93			X
**Bhattacharya et al.[279]**	2010	India	2008 - 2009	42	X	X	
**Arredondo-García et al.[280]**	2010	Mexico	2002 - 2005	150	X	X	
**Xue et al.[281]**	2010	China	2006 - 2008	171	X	X	
**Vestrheim et al.[282]**	2010	Norway	2006 - 2008	1102		X	X
**Vasoo et al.[283]**	2010	Singapore	2007 - 2008	59	X	X	X
**van Gils et al.[284]**	2010	Netherlands	2005 - 2008	123		X	X
**Techasaensiri et al.[285]**	2010	United States of America	1999 - 2008	191	X	X	X
**Talbert et al.[286]**	2010	Kenya	2001 - 2009	33	X	X	
**Srifeungfung et al.[287]**	2010	Thailand	2006 - 2009	106	X	X	
**Skalet et al.[288]**	2010	Ethiopia	2006 - 2007	98	X	X	
**Simões et al.[289]**	2010	Portugal	2006 - 2006	25	X	X	

**Schwarz et al.**[290]	2010	Ghana	2007 - 2009	20	X	X	
**Rijal et al.**[291]	2010	Nepal	2004 - 2008	46	X	X	
**Ochoa et al.**[292]	2010	Peru	2006 - 2008	101	X	X	X
**Mwenya et al.**[293]	2010	Zambia	2001 - 2003	93	X	X	
**Matsumoto et al.**[294]	2010	Japan	2004 - 2006	202	X	X	
**Janapatla et al.**[295]	2010	China	2005 - 2007	95	X	X	X
**Imöhl et al.**[296]	2010	Germany	1992 - 2006	2326		X	
**Imöhl et al.**[297]	2010	Germany	1997 - 2006	2052		X	
**Imöhl et al.**[298]	2010	Germany	1992 - 2008	2977		X	X
**Imöhl et al.**[299]	2010	Germany	1992 - 2008	11428		X	X
**Hsu et al.**[300]	2010	United States of America	2001 - 2007	428	X	X	
**Gómez-Barreto et al.**[301]	2010	Mexico	1997 - 2004	116	X	X	
**Franco et al.**[302]	2010	Brazil	2005 - 2005	686	X	X	X
**Eun et al.**[303]	2010	South Korea	1995 - 2005	131	X	X	
**Bettinger et al.**[304]	2010	Canada	2000 - 2007	4098	X	X	
**Aguiar et al.**[305]	2010	Portugal	1999 - 2008	577	X	X	X
**Liu et al.**[306]	2010	China	2004 – 2009	44	X	X	
**Borg et al.**[307]	2009	Jordan	2017 - 2018	1483	X	X	X
**Rodrigues et al.**[308]	2009	Portugal	2007 – 2007	305	X	X	X
**Karnezis et al.**[309]	2009	United States of America	1995 - 2006	164	X	X	
**Bere et al.**[310]	2009	Burkina Faso	2000 – 2001	232	X	X	
**Ndip et al.**[311]	2009	Cameroon	2004 - 2005	30	X	X	
**Sa-Leao et al.**[312]	2009	Portugal	2001 - 2006	867	X	X	X
**Velasquez et al.**[313]	2009	Brazil	2008-2008	92	X	X	
**Arifeen et al.**[314]	2009	Bangladesh	2004-2007	26	X	X	
**Falade et al.**[315]	2009	Nigeria	2005-2007	11	X	X	
**Perez-Trallero et al.**[316]	2009	Spain	1996-2007	91		X	
**Castaneda et al.**[317]	2009	Chile	2000-2005	8756	X	X	X
**Grivea et al.**[318]	2009	Greece	2005 - 2007	832		X	X
**Zaidi et al.**[319]	2009	Pakistan	2005-2006	15	X	X	
**Saha et al** [320]	2009	Bangladesh	2004-2005	139	X	X	
**Katsarolis et al.**[321]	2009	Greece	2004-2004	746	X	X	
**Otsuka et al.**[322]	2009	Japan	2002-2004	230	X	X	
**Mantese et al.**[323]	2009	Brazil	1998-2008	142	X	X	
**Corsco et al.**[324]	2009	Argentina	1993-2001	1499	X	X	
**Cekmez et al.**[325]	2009	Turkey	2007-2008	25		X	
**Dortet et al.**[326]	2009	France	2007-2007	59		X	X
**Rendi-Wagner et al.**[327]	2009	Austria	2001-2007	172		X	X
**Torun et al.**[328]	2009	Turkey	2008-2008	96		X	
**Leach et al.**[329]	2009	Australia	2003-2005	1362	X	X	
**Greenberg et al.**[330]	2008	Israel	1998-2005	1483	X	X	
**Sanchez-Tatay et al.**[331]	2008	Spain	2005-2006	194	X	X	

**Arnason et al.[332]**	2008	Iceland	1994-2005	272	X	X	
**Nantanda et al.[333]**	2008	Uganda	2005-2006	53	X	X	
**Shibl et al.[334]**	2008	Saudi Arabia	2000-2004	350	X	X	X
**Mokaddas et al.[335]**	2008	Kuwait	2004-2005	195	X		
**Munoz-Almagro et al.[336]**	2008	Spain	1997-2001	198		X	
**Perez et al.[337]**	2008	Spain	2000-2004	117		X	
**Winters et al[338]**	2008	Canada	2002-2005	239		X	
**Daikos et al.[339]**	2008	Greece	1985-2004	380	X	X	
**Dueger et al.[340]**	2008	Guatemala	2001-2006	554	X	X	X
**Brauteset et al.[341]**	2008	Norway	1998-2004	68	X	X	
**Heffernan et al.[342]**	2008	New Zealand	1998-2005	2132	X	X	
**Chong et al.[343]**	2008	Singapore	1997-2004	147	X	X	
**Sombero et al.[344]**	2008	Philippines	1994-2000	1013	X	X	
**Pirez et al.[345]**	2008	Uruguay	1998-2005	168	X	X	
**Kastrin et al.[346]**	2008	Slovenia	1998-2007	339		X	
**Cardosao et al.[347]**	2008	Multiple countries	1998-2002	240	X	X	
**Steenhoff et al.[348]**	2008	United States of America	1989-2006	21		X	
**Vestrheim et al.[349]**	2008	Norway	2006-2006	509		X	X
**Dunais et al.[350]**	2008	France	1996 - 2006	333	X	X	
**Ishiwada et al.[351]**	2008	Japan	2003-2005	115	X	X	
**Guzvinec et al.[352]**	2008	Croatia	2005-2006	100	X	X	X
**Abut et al.[353]**	2008	Turkey	2004-2005	25	X	X	
**Villasenor-Sierra et al.[354]**	2008	Mexico	2000-2005	50	X	X	X
**Liu et al.[355]**	2008	China	2005-2006	31	X	X	
**Montagnani et al.[356]**	2008	Italy	1992-2006	334		X	
**Ozdemir et al.[357]**	2008	Turkey	2003-2004	118	X	X	
**Bayer at al.[358]**	2008	Turkey	2003-2004	175	X	X	
**Grivea et al.[359]**	2008	Greece	2005-2007	867	X	X	
**Sigauque et al.[360]**	2008	Mozambique	1998-2003	26	X	X	
**Park et al.[361]**	2008	United States	2000-2004	355	X	X	X
**Dias et al.[362]**	2007	Portugal	1999-2004	295	X	X	
**Rivera-Olivero et al.[363]**	2007	Venezuela	2004-2005	161	X	X	
**Messina et al.[364]**	2007	United States	1999-2005	121	X	X	X
**Bettinger et al.[365]**	2007	Canada	1998-2003	1870		X	X
**Chen et al.[366]**	2007	Taiwan	2001-2002	43	X	X	
**Uzuner et al.[367]**	2007	Turkey	2004-2004	94	X	X	
**Wattal et al.[368]**	2007	India	2006-2006	13	X	X	
**Nyandiko et al.[369]**	2007	Kenya	2003-2003	27	X	X	
**Poulakous et al.[370]**	2007	Greece	2004-2006	780		X	
**Pagliano et al.[371]**	2007	Italy	1997-2005	64		X	
**Blossom et al.[372]**	2007	Uganda	1995-1995	113	X	X	X
**Tamm et al.[373]**	2007	Estonia	2001-2002	299	X	X	

**Reinert et al.**[374]	2007	Germany	1997-2004	1517	X	X	X
**Brooks et al.**[375]	2007	Bangladesh	2004-2006	34	X	X	
**Roche et al.**[376]	2007	United Kingdom	2003-2003	120	X	X	
**Aristegui et al.**[377]	2007	Spain	1989-2003	277		X	
**Aslan et al.**[378]	2007	Turkey	2003-2004	201	X	X	
**Espinosa de los Monte et al.**[379]	2007	Mexico	2002-2002	829	X	X	X
**Katz et al.**[380]	2007	Russia	2003 - 2003	83	X	X	X
**Vieira et al.**[381]	2007	Brazil	1995 - 2004	67	X	X	
**Souli et al.**[382]	2007	Greece	2000 - 2003	843	X	X	
**Schultsz et al.**[383]	2007	Vietnam	2003 - 2004	536	X	X	
**Phongsamart et al.**[384]	2007	Thailand	2000 - 2005	115	X	X	
**Orrett et al.**[385]	2007	Trinidad and Tobago	1997 - 2002	40	X	X	
**Pineda et al.**[386]	2007	Spain	1990 - 2000	105	X	X	
**Zanchi et al.**[387]	2006	Italy	1992 - 2004	26	X	X	
**Sakata et al.**[388]	2006	Japan	2003 - 2005	16	X	X	
**Whitney et al.**[389]	2006	United States of America	2001 - 2004	782		X	
**Levidiotou et al.**[390]	2006	Greece	2000 - 2004	398	X	X	
**García-Suárez et al.**[391]	2006	Spain	1998 - 2004	39		X	
**Aarson et al.**[392]	2006	Iceland	1993 - 2003	1335	X	X	
**Volonakis et al.**[393]	2006	Greece	2000 - 2003	844	X	X	
**Roca et al.**[394]	2006	Mozambique	2001 - 2003	211	X	X	
**Russell et al.**[395]	2006	Fiji	2003 - 2004	246	X	X	
**Tarallo et al.**[396]	2006	Italy	2002 - 2003	42	X	X	
**Vergison et al.**[397]	2006	Belgium	2002 - 2003	280	X	X	X
**Stratchounski et al.**[398]	2006	Russia	2001 - 2002	2056	X	X	
**Lauderdale et al.**[399]	2006	China	1997 - 2003	541	X	X	
**Chen et al.**[400]	2006	China	2002 - 2003	147	X	X	
**De Schutter et al.**[401]	2006	Belgium	1988 - 2002	70	X	X	
**Wexler et al.**[402]	2006	Israel	1986 - 1997	100	X	X	
**Lin et al.**[403]	2006	China	1999 - 2004	286	X	X	
**Dias et al.**[404]	2006	Portugal	1994 - 2004	330	X	X	
**Žemličková et al.**[405]	2006	Czech Republic	2004 - 2005	165	X	X	
**Paraskakis et al.**[406]	2006	Greece	2001 - 2004	392	X	X	
**Yalçın et al.**[407]	2006	Turkey	2001 - 2004	93	X	X	
**Kyaw et al.**[408]	2006	United States of America	1999 - 2004	1967	X	X	X
**Cardozo et al.**[409]	2006	Brazil	2002 - 2003	83	X	X	X
**Quintero et al.**[410]	2006	Venezuela	2006 - 2006	30	X	X	
**Valles et al.**[411]	2006	Mozambique	2002 - 2003	375	X	X	X
**Steenhoff et al.**[412]	2006	United States of America	1999 - 2005	188	X	X	
**Laval et al.**[413]	2006	Brazil	2000 - 2001	125	X	X	X
**Ahmad Al Khorasani et al.**[414]	2006	Yemen	1999 - 2001	42	X	X	

**Siedler et al.**[415]	2005	Germany	1997 - 2002	1023	X	X	
**Demachy et al.**[416]	2005	France	1999 - 1999	537	X	X	
**Mato et al.**[417]	2005	Portugal	2001 - 2003	3539	X	X	
**Charvériat et al.**[418]	2005	New Caledonia	2002 - 2003	544	X	X	
**Ogunlesi et al.**[419]	2005	Nigeria	1998 - 2003	42	X	X	
**Lauderdale et al.**[420]	2005	China	2002 - 2002	32	X	X	
**Solórzano-Santos et al.**[421]	2005	Mexico	2002 - 2003	122	X	X	
**Babay et al.**[422]	2005	Saudi Arabia	2004 - 2004	8	X	X	
**Hennessy et al.**[423]	2005	United States of America	1998 - 2002	199	X	X	
**Hussain et al.** [424]	2005	United Kingdom	2001 - 2002	932	X	X	
**Guillemot et al.**[425]	2005	France	2000 - 2000	112	X	X	
**Huang et al.**[426]	2005	United States of America	2003 - 2004	232	X	X	
**Arri et al.**[427]	2005	Germany	1993 - 2002	108	X	X	
**Bayraktar et al.**[428]	2005	Turkey	2003 - 2003	848	X	X	
**Thomasson et al.**[429]	2005	Iceland	1992 - 1999	640	X	X	X
**McEllistrem et al.**[430]	2005	United States of America	1995 - 2001	344	X	X	
**Byington et al.**[431]	2005	United States of America	1996 - 2003	234	X	X	
**Ochoa et al.**[432]	2005	Peru	1996 - 2003	272	X	X	
**Wasfy et al.**[433]	2005	Egypt	1998 - 2003	15	X	X	
**Frazão et al.**[434]	2005	Portugal	2001 - 2003	633	X	X	
**Watson et al.**[435]	2005	Australia	2003 - 2003	622	X	X	X
**Jain et al.**[436]	2005	India	2000 - 2002	1287	X	X	
**Grenon et al.**[437]	2005	Argentina	1998 - 2001	101	X	X	
**Pai et al.**[438]	2005	United States of America	1999 - 2004	232			X
**Haddy et al.**[439]	2004	United States of America	1997 - 1999	117	X	X	
**Nunes et al.**[440]	2004	Portugal	1999 - 1999	591	X	X	
**Gazi et al.**[441]	2004	Turkey	2002 - 2003	240	X	X	
**Oteo et al.**[442]	2004	Spain	2001 - 2003	1873		X	
**Ghaffar et al.**[443]	2004	United States of America	2000 - 2001	294		X	
**Sulikowska et al.**[444]	2004	Poland	2000 - 2001	152	X	X	
**Malfroot et al.**[445]	2004	Belgium	2000 - 2001	99	X	X	
**Decousser et al.**[446]	2004	France	2000 - 2002	32	X	X	
**Ho et al.**[447]	2004	China	1994 - 2001	88	X	X	X
**Ho et al.**[448]	2004	China	1999 - 2000	471	X	X	
**Mufson et al.**[449]	2004	United States of America	1978 - 2003	70		X	
**Campbell et al.**[450]	2004	Mali	2002 - 2003	106	X	X	
**Serrano et al.**[451]	2004	Portugal	1992 - 2002	86	X	X	
**Rendi-Wagner et al.**[452]	2004	Austria	2001 - 2003	56		X	
**Immergluck et al.**[453]	2004	United States of America	1998 - 1999	47	X	X	
**McGregor et al.**[454]	2004	Jamaica	1995 - 1999	117	X	X	
**Regev-Yochay et al.**[455]	2004	Israel	2001 - 2001	216	X	X	
**Latorre et al.**[456]	2004	Spain	1989 - 2000	96	X	X	

**Decousser et al.[457]**	2004	France	1997 - 2002	73	X	X	X
**Watson et al.[458]**	2003	Australia	2001 - 2002	1355	X	X	
**Quagliraello et al.[459]**	2003	Vietnam	1996 - 1999	433	X	X	
**Henriques Normark et al.[460]**	2003	Sweden	1997 - 1997	246	X	X	
**Ulloa-Gutierrez et al.[461]**	2003	Costa Rica	1995 - 2001	84	X	X	
**Saha et al.[462]**	2003	Bangladesh	1999 - 2000	1411	X	X	X
**Greenberg et al.[463]**	2003	Israel	1998 - 1999	437	X	X	
**Pantosti et al.[464]**	2003	Italy	1997 - 2000	72	X	X	
**Hoffman et al.[465]**	2003	United States of America	1993 - 2001	21		X	
**Sirinavin et al.[466]**	2003	Thailand	1971 - 2000	191	X	X	
**Klugman et al.[467]**	2003	South Africa	1998 - 2000	421	X	X	
**Hedlund et al.[468]**	2003	Sweden	1998 - 2001	204	X	X	
**Pallares et al.[469]**	2003	Spain	2000 - 2002	782		X	
**Batt et al.[470]**	2003	Tanzania	2000 - 2000	141	X	X	
**Lee et al.[471]**	2003	China	2000 - 2001	22	X	X	
**Zhao et al.[472]**	2003	China	2000 - 2001	112	X	X	
**Waterer et al.[473]**	2003	United States of America	1996 - 2001	486		X	
**Feikin et al.[474]**	2003	Malawi	1997 - 1997	694	X	X	
**Chen et al.[475]**	2003	China	1996 - 2001	117	X	X	
**Dagan et al.[476]**	2003	Israel	1996 - 1997	2446	X	X	X
**Givon-Lavi et al.[477]**	2003	Israel	1996 - 1999	43	X	X	
**Whitney et al.[478]**	2003	United States of America	1999 - 2001	902	X	X	
**Hjaltested et al.[479]**	2003	Iceland	1999 - 1999	406	X	X	
**Berezin et al.[480]**	2002	Brazil	1994 - 1998	55	X	X	X
**Rey et al.[481]**	2002	Brazil	1998 - 1998	241	X	X	
**Denno et al.[482]**	2002	Ghana	1996 - 1996	142	X	X	
**Coles et al.[483]**	2002	India	1998 - 1999	323	X	X	
**Hills et al.[484]**	2002	Australia	2001 - 2001	45	X	X	
**Roche et al.[485]**	2002	Australia	2001 - 2001	188	X	X	
**Lagos et al.[486]**	2002	Chile	1994 - 2001	399	X	X	
**Mwangi et al.[487]**	2002	Kenya	1994 - 1998	81	X	X	
**Cullotta et al.[488]**	2002	Peru	2000 - 2000	146	X	X	
**Syrogiannopoulos et al.[489]**	2002	Greece	1997 - 1999	781	X	X	
**Kellner et al.[490]**	2002	Canada	1991 - 1999	297	X	X	
**Siu et al.[491]**	2002	China	1998 - 1999	99	X	X	
**Arason et al.[492]**	2002	Iceland	1998 - 1998	384	X	X	
**Christie et al.[493]**	2002	Australia	1994 - 2000	76	X	X	
**Quach et al.[494]**	2002	Canada	1989 - 1998	144	X	X	X
**Rey et al.[495]**	2002	Brazil	1998 - 1998	441	X	X	X
**McMaster et al.[496]**	2002	Australia	1994 - 1999	103	X	X	
**Herruzo et al.[497]**	2002	Spain	1999 - 2000	20	X	X	
**Lonks et al.[498]**	2002	Spain	1989 - 2000	69	X	X	

**Masuda et al.**[499]	2002	Japan	1991 - 1991	94	X	X	
**Flamaing et al.**[500]	2002	Belgium	1994 - 2000	843	X	X	
**Bakir et al.**[501]	2002	Turkey	2000 - 2000	118	X	X	
**Hennessy et al.**[502]	2002	United States of America	1998 - 1998	116	X	X	
**Contreras et al.**[503]	2002	Chile	1994 - 1999	78	X	X	
**Petrosillo et al.**[504]	2002	Italy	1999 - 1999	85	X	X	
**Ma et al.**[505]	2002	China	1990 - 2000	72	X	X	
**Gómez-Barreto et al.**[506]	2002	Mexico	1997 - 1999	178	X	X	
**Cizman et al.**[507]	2002	Slovenia	1997 - 2000	151	X	X	
**Nasrin et al.**[508]	2002	Australia	1997 - 1999	631	X	X	
**Greenberg et al.**[509]	2002	Canada	1991 - 1998	1538	X	X	
**Albanese et al.**[510]	2002	United States of America	1995 - 1997	387	X	X	
**Scheifele et al.**[511]	2001	Canada	1991 - 1998	1847	X	X	
**Rossi et al.**[512]	2001	Brazil	1999 - 2000	350	X	X	
**Hortal et al.**[513]	2001	Uruguay	1993 - 1999	2899	X	X	
**Marchisio et al.**[514]	2001	Italy	1996 - 1996	135	X	X	
**Buckingham et al.**[515]	2001	United States of America	1991 - 1999	86	X	X	
**Di Fabio et al.**[516]	2001	Multiple Countries	1994 - 1998	1060	X	X	
**Stovall et al.**[517]	2001	United States of America	1990 - 2000	80		X	
**Boost et al.**[518]	2001	China	2001 - 2001	51	X	X	
**Chiu et al.**[519]	2001	China	1999 - 2000	383	X	X	
**Kacou-N'douba et al.**[520]	2001	Cote d Ivoire	1997 - 1998	138	X	X	
**Fraser et al.**[521]	2001	Israel	1989 - 1998	479	X	X	
**Doern et al.**[522]	2001	United States of America	1999 - 2000	447		X	
**Joloba et al.**[523]	2001	Uganda	1995 - 1995	115	X	X	X
**Kaplan et al.**[524]	2001	United States of America	1993 - 1999	2100	X	X	
**Lee et al.**[525]	2001	Multi-Country	1998 - 1999	1105	X	X	
**Pírez et al.**[526]	2001	Uruguay	1997 - 1998	41	X	X	
**Nascimento-Carvalho et al.**[527]	2001	Brazil	1997 - 1999	19	X	X	X
**Markovska et al.**[528]	2001	Bulgaria	1999 - 1999	85	X	X	
**Soewignjo et al.**[529]	2001	Indonesia	1997 - 1997	221	X	X	
**Ip et al.**[530]	2001	China	1993 - 1997	1922	X	X	
**Porat et al.**[531]	2001	Israel	1995 - 1999	68			X
**Naaher et al.**[532]	2000	Estonia	1999 - 2000	182	X	X	
**Huebner et al.**[533]	2000	South Africa	1995 - 1998	1817	X	X	
**Huebner et al.**[534]	2000	South Africa	2000 - 2000	121	X	X	
**Fenoll et al.**[535]	2000	Spain	1990 - 1999	1398	X	X	
**Kaltoft et al.**[536]	2000	Denmark	1990 - 1999	803	X	X	
**Miller et al.**[537]	2000	United Kingdom	1996 - 1998	1985	X	X	
**Melander et al.**[538]	2000	Sweden	1997 - 1998	68150	X	X	
**Whitney et al.**[539]	2000	United States of America	1995 - 1998	1020	X	X	X
**Perrone et al.**[540]	2000	United States of America	1996 - 1998	12	X	X	

**Westwood et al.[541]**	2000	South Africa	1996 - 1996	16	X	X
**McIntyre et al.[542]**	2000	Australia	1997 - 1999	372	X	X
**Wolf et al.[543]**	2000	Brazil	1998 - 1998	205	X	X
**Syrogiannopoulos et al.[544]**	2000	Greece	1997 - 1999	781	X	X
**Lupisan et al.[545]**	2000	Philippines	1994 - 1996	246	X	X
**Marco et al.[546]**	2000	Spain	1996 - 1997	125	X	X
**von Kries et al.[547]**	2000	Germany	1997 - 1998	304	X	X
**Rowland et al.[548]**	2000	Australia	1997 - 1999	68	X	X
**Sá-Leão et al.[549]**	2000	Portugal	2000 - 2000	177	X	X
**Polack et al.[550]**	2000	United States of America	1997 - 1997	33	X	X
**Scheifele et al.[551]**	2000	Canada	1991 - 1998	1528	X	X
**Borres et al.[552]**	2000	Sweden	1996 - 1997	322	X	X
**Raymond et al.[553]**	2000	France	1996 - 1996	111	X	X
**Syrogiannopoulos et al.[554]**	2000	Greece	1997 - 1998	421	X	X
**Rowe et al.[555]**	2000	Central African Republic	1995 - 1995	289	X	X
**Walsh et al.[556]**	2000	Malawi	2000 - 2000	57	X	X
**Stratchounski et al.[557]**	2000	Russia	2000 - 2000	305	X	X
**Parry et al.[558]**	2000	Vietnam	2000 - 2000	399	X	X

References

1 El-Kholy A, Badawy M, Gad M, Soliman M. Serotypes and Antimicrobial Susceptibility of Nasopharyngeal Isolates of Streptococcus pneumoniae from Children Less Than 5 Years Old in Egypt. *Infect Drug Resist* 2020; **Volume 13**: 3669–77.

2 Al-Lahham A. Multicenter study of pneumococcal carriage in children 2 to 4 years of age in the winter seasons of 2017-2019 in Irbid and Madaba governorates of Jordan. *PLOS ONE* 2020; **15**: e0237247.

3 Vidanapathirana G, Angulmaduwa S, Munasinghe T, *et al.* Pneumococcal colonization among healthy and hospitalized vaccine-naive Sri Lankan children. *Vaccine* 2020; **38**: 7308–15.

4 Uddén F, Filipe M, Slotved H-C, *et al.* Pneumococcal carriage among children aged 4 – 12 years in Angola 4 years after the introduction of a pneumococcal conjugate vaccine. *Vaccine* 2020; **38**: 7928–37.

5 Ustundag G, Karadag-Oncel E, Sen-Tas S, *et al.* One year period of invasive pneumococcal disease in children from a tertiary care hospital in Turkey in the post-vaccine era. *Hum Vaccines Immunother* 2020; : 1–4.

6 Goh SL, Kee BP, Abdul Jabar K, *et al.* Molecular detection and genotypic characterisation of *Streptococcus pneumoniae* isolated from children in Malaysia. *Pathog Glob Health* 2020; **114**: 46–54.

7 Tsai M-H, Liao S-L, Chiu C-Y, *et al.* Longitudinal investigation of nasopharyngeal pneumococcal carriage in early childhood: The PATCH birth cohort study. *PLOS ONE* 2020; **15**: e0237871.

8 Zhao C, Xie Y, Zhang F, *et al.* Investigation of Antibiotic Resistance, Serotype Distribution, and Genetic Characteristics of 164 Invasive Streptococcus pneumoniae from North China Between April 2016 and October 2017. *Infect Drug Resist* 2020; **Volume 13**: 2117–28.

9 Wouters I, Desmet S, Van Heirstraeten L, *et al.* How nasopharyngeal pneumococcal carriage evolved during and after a PCV13-to-PCV10 vaccination programme switch in Belgium, 2016 to 2018. *Eurosurveillance* 2020; **25**. DOI:10.2807/1560-7917.ES.2020.25.5.1900303.

10 Dai VTT, Beissbarth J, Thanh PV, *et al.* Hospital surveillance predicts community pneumococcal antibiotic resistance in Vietnam. *J Antimicrob Chemother* 2020; **75**: 2902–6.

11 Kawaguchiya M, Urushibara N, Aung MS, *et al.* High prevalence of antimicrobial resistance in non-vaccine serotypes of non-invasive/colonization isolates of Streptococcus pneumoniae: A cross-sectional study eight years after the licensure of conjugate vaccine in Japan. *J Infect Public Health* 2020; **13**: 1094–100.

12 Vorobieva S. Jensen V, Furberg A-S, Slotved H-C, *et al.* Epidemiological and molecular characterization of Streptococcus pneumoniae carriage strains in pre-school children in

Arkhangelsk, northern European Russia, prior to the introduction of conjugate pneumococcal vaccines. *BMC Infect Dis* 2020; **20**: 279.

13 Hernstadt H, Cheung A, Hurem D, *et al.* Changing Epidemiology and Predisposing Factors for Invasive Pneumococcal Disease at Two Australian Tertiary Hospitals: *Pediatr Infect Dis J* 2020; **39**: 1–6.

14 Manenzhe RI, Dube FS, Wright M, *et al.* Characterization of Pneumococcal Colonization Dynamics and Antimicrobial Resistance Using Shotgun Metagenomic Sequencing in Intensively Sampled South African Infants. *Front Public Health* 2020; **8**: 543898.

15 Pelkonen T, Urtti S, dos Anjos E, *et al.* Aetiology of bacterial meningitis in infants aged <90 days: Prospective surveillance in Luanda, Angola. *Int J Infect Dis* 2020; **97**: 251–7.

16 Lo SW, Gladstone RA, Tonder AJ van, *et al.* Pneumococcal lineages associated with serotype replacement and antibiotic resistance in childhood invasive pneumococcal disease in the post-PCV13 era: an international whole-genome sequencing study. *Lancet Infect Dis* 2019; **19**: 759–69.

17 Mohammadi Gharibani K, Azami A, Parvizi M, Khademi F, Mousavi SF, Arzanlou M. High Frequency of Macrolide-Resistant Streptococcus pneumoniae Colonization in Respiratory Tract of Healthy Children in Ardabil, Iran. *Tanaffos* 2019; **18**: 118–25.

18 Kaplan SL, Barson WJ, Lin PL, *et al.* Invasive Pneumococcal Disease in Children's Hospitals: 2014-2017. *Pediatrics* 2019; **144**. DOI:10.1542/peds.2019-0567.

19 Danino D, Givon-Lavi N, Ben-Shimol S, Greenberg D, Dagan R. Understanding the Evolution of Antibiotic-nonsusceptible Pneumococcal Nasopharyngeal Colonization Following Pneumococcal Conjugate Vaccine Implementation in Young Children. *Clin Infect Dis* 2019; **69**: 648–56.

20 Nakano S, Fujisawa T, Ito Y, *et al.* Nationwide surveillance of paediatric invasive and non-invasive pneumococcal disease in Japan after the introduction of the 13-valent conjugated vaccine, 2015–2017. *Vaccine* 2020; **38**: 1818–24.

21 Huang Y-C, Lin C-F, Ting P-J, *et al.* Respiratory pathogens – Some altered antibiotic susceptibility after implementation of pneumococcus vaccine and antibiotic control strategies. *J Microbiol Immunol Infect* 2019; : S1684118219301513.

22 Corcoran M, Mereckiene J, Cotter S, Murchan S, Cunney R, Humphreys H. Invasive Streptococcus pneumoniae Infections and Vaccine Failures in Children in Ireland From the Postvaccine Era From 2007 to 2018: *Pediatr Infect Dis J* 2019; : 1.

23 Meropol SB, Jacobs MR, Stange KC, Bajaksouzian S, Bonomo RA. Longitudinal Colonization With Streptococcus pneumoniae During the First Year of Life in a Healthy Newborn Cohort. *J Pediatr Infect Dis Soc* 2019; : piz068.

24 Dilagui I, Moussair FZ, Loqman S, *et al.* Streptococcus pneumoniae carriage among febrile children at the time of PCV-10 immunization in pediatric emergencies at Mohammed VI University Hospital Centre in Marrakesh (Morocco). *Arch Pédiatrie* 2019; **26**: 453–8.

25 Bao Y, Wang Q, Yao K, *et al.* The changing phenotypes and genotypes of invasive pneumococcal isolates from children in Shenzhen during 2013–2017. *Vaccine* 2019; **37**: 7248–55.

26 Kourna Hama M, Khan D, Laouali B, *et al.* Pediatric Bacterial Meningitis Surveillance in Niger: Increased Importance of Neisseria meningitidis Serogroup C, and a Decrease in Streptococcus pneumoniae Following 13-Valent Pneumococcal Conjugate Vaccine Introduction. *Clin Infect Dis* 2019; **69**: S133–9.

27 Dayie NTKD, Tettey EY, Newman MJ, *et al.* Pneumococcal carriage among children under five in Accra, Ghana, five years after the introduction of pneumococcal conjugate vaccine. *BMC Pediatr* 2019; **19**: 316.

28 Chen C-H, Su L-H, Li H-C, *et al.* Evaluation of the impact of 13-valent pneumococcal conjugate vaccine immunization in children by surveillance of culture-confirmed pneumococcal disease: A prospective clinical microbiological study. *Vaccine* 2019; **37**: 5147–52.

29 Sutcliffe CG, Shet A, Varghese R, *et al.* Nasopharyngeal carriage of Streptococcus pneumoniae serotypes among children in India prior to the introduction of pneumococcal conjugate vaccines: a cross-sectional study. *BMC Infect Dis* 2019; **19**: 605.

30 Shen H, Zhu C, Liu X, *et al.* The etiology of acute meningitis and encephalitis syndromes in a sentinel pediatric hospital, Shenzhen, China. *BMC Infect Dis* 2019; **19**: 560.

31 Takeuchi N, Ohkusu M, Wada N, *et al.* Molecular typing, antibiotic susceptibility, and biofilm production in nonencapsulated Streptococcus pneumoniae isolated from children in Japan. *J Infect Chemother* 2019; **25**: 750–7.

32 Malaker R, Saha S, Hanif M, *et al.* Invasive Pneumococcal Infections in Children with Nephrotic Syndrome in Bangladesh: *Pediatr Infect Dis J* 2019; **38**: 798–803.

33 Haile AA, Gidebo DD, Ali MM. Colonization rate of Streptococcus pneumoniae, its associated factors and antimicrobial susceptibility pattern among children attending kindergarten school in Hawassa, southern Ethiopia. *BMC Res Notes* 2019; **12**: 344.

34 Wada FW, Tufa EG, Berheto TM, Solomon FB. Nasopharyngeal carriage of Streptococcus pneumoniae and antimicrobial susceptibility pattern among school children in South Ethiopia: post-vaccination era. *BMC Res Notes* 2019; **12**: 306.

35 Lee M-C, Kuo K-C. The clinical implication of serotype distribution and drug resistance of invasive pneumococcal disease in children: A single center study in southern Taiwan during 2010–2016. *J Microbiol Immunol Infect* 2019; **52**: 937–46.

36 Manenzhe RI, Moodley C, Abdulgader SM, *et al.* Nasopharyngeal Carriage of Antimicrobial-Resistant Pneumococci in an Intensively Sampled South African Birth Cohort. *Front Microbiol* 2019; **10**: 610.

37 Kobayashi M, Bigogo G, Kim L, *et al.* Impact of 10-valent Pneumococcal Conjugate Vaccine Introduction on Pneumococcal Carriage and Antibiotic Susceptibility Patterns among Children aged <5 Years and Adults with HIV Infection, Kenya 2009–2013. *Clin Infect Dis* 2019; published online April 9. DOI:10.1093/cid/ciz285.

38 Mayanskiy N, Kulichenko T, Alyabieva N, *et al.* Changing serotype distribution and resistance patterns among pediatric nasopharyngeal pneumococci collected in Moscow, 2010–2017. *Diagn Microbiol Infect Dis* 2019; **94**: 385–90.

39 Luna-Muschi A, Castillo-Tokumori F, Deza MP, *et al.* Invasive pneumococcal disease in hospitalised children from Lima, Peru before and after introduction of the 7-valent conjugated vaccine. *Epidemiol Infect* 2019; **147**: e91.

40 Negash AA, Asrat D, Abebe W, *et al.* Bacteremic Community-Acquired Pneumonia in Ethiopian Children: Etiology, Antibiotic Resistance, Risk Factors, and Clinical Outcome. *Open Forum Infect Dis* 2019; **6**. DOI:10.1093/ofid/ofz029.

41 Polkowska A, Skoczyńska A, Paradowska-Stankiewicz I, *et al.* Pneumococcal meningitis before the introduction of 10-valent pneumococcal conjugate vaccine into the National Childhood Immunization Program in Poland. *Vaccine* 2019; **37**: 1365–73.

42 Shakur SM, Whitehall J, Mudgil P. Pediatric bloodstream infections in metropolitan Australia. *World J Pediatr* 2019; **15**: 161–7.

43 Yan Z, Cui Y, Zhou W, *et al.* Molecular characterization of Streptococcus pneumoniae in children living in southwest China and assessment of a potential protein vaccine, rPfbA. *Vaccine* 2019; **37**: 721–31.

44 Shi W, Li J, Dong F, *et al.* Serotype distribution, antibiotic resistance pattern, and multilocus sequence types of invasive Streptococcus pneumoniae isolates in two tertiary pediatric hospitals in Beijing prior to PCV13 availability. *Expert Rev Vaccines* 2019; **18**: 89–94.

45 Al-Jardani A, Al Rashdi A, Al Jaaidi A, *et al.* Serotype distribution and antibiotic resistance among invasive Streptococcus pneumoniae from Oman post 13-valent vaccine introduction. *Int J Infect Dis* 2019; **85**: 135–40.

46 Kavalari ID, Fuursted K, Krogfelt KA, Slotved H-C. Molecular characterization and epidemiology of Streptococcus pneumoniae serotype 24F in Denmark. *Sci Rep* 2019; **9**: 5481.

47 Pourakbari B, Mahmoudi S, Moradzadeh M, *et al.* Antimicrobial Resistance Patterns of the Gram-positive Bacteria Isolated from Children with Bloodstream Infection in an Iranian Referral Hospital: A 6-year Study. *Infect Disord Drug Targets* 2018; **18**: 136–44.

48 Yun KW, Choi EH, Lee HJ, *et al.* Genetic structures of invasive Streptococcus pneumoniae isolates from Korean children obtained between 1995 and 2013. *BMC Infect Dis* 2018; **18**: 268.

49 Verani JR, Massora S, Acácio S, *et al.* Nasopharyngeal carriage of Streptococcus pneumoniae among HIV-infected and –uninfected children <5 years of age before introduction of pneumococcal conjugate vaccine in Mozambique. *PLOS ONE* 2018; **13**: e0191113.

50 Ubukata K, Takata M, Morozumi M, *et al.* Effects of Pneumococcal Conjugate Vaccine on Genotypic Penicillin Resistance and Serotype Changes, Japan, 2010–2017. *Emerg Infect Dis* 2018; **24**: 2010–20.

51 Mulu W, Yizengaw E, Alemu M, *et al.* Pharyngeal colonization and drug resistance profiles of Morraxella catarrrhalis, Streptococcus pneumoniae, Staphylococcus aureus, and Haemophilus influenzae among HIV infected children attending ART Clinic of Felegehiwot Referral Hospital, Ethiopia. *PLOS ONE* 2018; **13**: e0196722.

52 Debess Magnussen M, Erlendsdóttir H, Gaini S, Gudnason T, Kristinsson KG. Streptococcus pneumoniae: Antimicrobial Resistance and Serotypes of Strains Carried by Children and Causing Invasive Disease in the Faroe Islands. *Microb Drug Resist* 2018; **24**: 1507–12.

53 Khan F, Khan MA, Ahmed N, *et al.* Molecular Characterization of Pneumococcal Surface Protein A (PspA), Serotype Distribution and Antibiotic Susceptibility of Streptococcus pneumoniae Strains Isolated from Pakistan. *Infect Dis Ther* 2018; **7**: 277–89.

54 Karlowsky JA, Adam HJ, Golden AR, *et al.* Antimicrobial susceptibility testing of invasive isolates of Streptococcus pneumoniae from Canadian patients: the SAVE study, 2011–15. *J Antimicrob Chemother* 2018; **73**: vii5–11.

55 Chan KC, Ip M, Chong PS, Li AM, Lam HS, Nelson EA. Nasopharyngeal colonisation and antimicrobial resistance of Streptococcus pneumoniae in Hong Kong children younger than 2 years. *Hong Kong Med J Xianggang Yi Xue Za Zhi* 2018; **24 Suppl 6**: 4–7.

56 Bojang A, Camara B, Jagne Cox I, *et al.* Long-term Impact of Oral Azithromycin Taken by Gambian Women During Labor on Prevalence and Antibiotic Susceptibility of Streptococcus pneumoniae and Staphylococcus aureus in Their Infants: Follow-up of a Randomized Clinical Trial. *Clin Infect Dis* 2018; **67**: 1191–7.

57 Santana Hernández M, Aguiar-Santana IA, Artiles Campelo F, Colino Gil E. Paediatric invasive pneumococcal disease on the island of Gran Canaria: 16-year prospective study (2001–2016). *Enfermedades Infecc Microbiol Clínica* 2018; **36**: 607–11.

58 Arushothy R, Ahmad N, Amran F, Hashim R, Samsudin N, Azih CRC. Pneumococcal serotype distribution and antibiotic susceptibility in Malaysia: A four-year study (2014–2017) on invasive paediatric isolates. *Int J Infect Dis* 2019; **80**: 129–33.

59 Cai K, Wang Y, Guo Z, Xu X, Li H, Zhang Q. Clinical characteristics and antimicrobial resistance of pneumococcal isolates of pediatric invasive pneumococcal disease in China. *Infect Drug Resist* 2018; **Volume 11**: 2461–9.

60 Berger Y, Adler A, Ariel T, Rokney A, Averbuch D, Grisaru-Soen G. Paediatric community-acquired bacteraemia, pneumococcal invasive disease and antibiotic resistance fell after the pneumococcal conjugate vaccine was introduced. *Acta Paediatr* 2019; **108**: 1321–8.

61 Birindwa AM, Emgård M, Nordén R, *et al.* High rate of antibiotic resistance among pneumococci carried by healthy children in the eastern part of the Democratic Republic of the Congo. *BMC Pediatr* 2018; **18**: 361.

62 Kastrin T, Paragi M, Erčulj V, Žohar Čretnik T, Bajec T, Čižman M. Lack of correlation between reduced outpatient consumption of macrolides and macrolide resistance of invasive Streptococcus pneumoniae isolates in Slovenia during 1997–2017. *J Glob Antimicrob Resist* 2019; **16**: 242–8.

63 Quirk SJ, Haraldsson G, Erlendsdóttir H, *et al.* Effect of Vaccination on Pneumococci Isolated from the Nasopharynx of Healthy Children and the Middle Ear of Children with Otitis Media in Iceland. *J Clin Microbiol* 2018; **56**: e01046-18, /jcm/56/12/e01046-18.atom.

64 Ho P-L, Law PY-T, Chiu SS. Increase in incidence of invasive pneumococcal disease caused by serotype 3 in children eight years after the introduction of the pneumococcal conjugate vaccine in Hong Kong. *Hum Vaccines Immunother* 2019; **15**: 455–8.

65 Cassiolato AP, Almeida SCG, Andrade AL, Minamisava R, Brandileone MC de C. Expansion of the multidrug-resistant clonal complex 320 among invasive Streptococcus pneumoniae serotype 19A after the introduction of a ten-valent pneumococcal conjugate vaccine in Brazil. *PloS One* 2018; **13**: e0208211.

66 Rockett RJ, Oftadeh S, Bachmann NL, *et al.* Genome-wide analysis of Streptococcus pneumoniae serogroup 19 in the decade after the introduction of pneumococcal conjugate vaccines in Australia. *Sci Rep* 2018; **8**: 16969.

67 John J, Varghese R, Lionell J, Neeravi A, Veeraraghavan B. Non-vaccine Pneumococcal Serotypes Among Children with Invasive Pneumococcal Disease. *Indian Pediatr* 2018; **55**: 874–6.

68 Chen H-H, Hsu M-H, Wu T-L, *et al.* Non-typeable Streptococcus pneumoniae infection in a medical center in Taiwan after wide use of pneumococcal conjugate vaccine. *J Microbiol Immunol Infect Wei Mian Yu Gan Ran Za Zhi* 2020; **53**: 94–8.

69 Zhang X, Tian J, Shan W, *et al.* Characteristics of pediatric invasive pneumococcal diseases and the pneumococcal isolates in Suzhou, China before introduction of PCV13. *Vaccine* 2017; **35**: 4119–25.

70 Wang J, Liu F, Ao P, *et al.* Detection of Serotype Distribution and Drug Resistance of Streptococcus Pneumoniae Isolated From Pediatric Patients. *Lab Med* 2017; **48**: 39–45.

71 Toledo ME, Casanova MF, Linares-Pérez N, *et al.* Prevalence of Pneumococcal Nasopharyngeal Carriage Among Children 2–18 Months of Age: Baseline Study Pre Introduction of Pneumococcal Vaccination in Cuba. *Pediatr Infect Dis J* 2017; **36**: e22–8.

72 Sihvonen R, Siira L, Toropainen M, Kuusela P, Pätäri-Sampo A. Streptococcus pneumoniae antimicrobial resistance decreased in the Helsinki Metropolitan Area after routine 10-valent pneumococcal conjugate vaccination of infants in Finland. *Eur J Clin Microbiol Infect Dis* 2017; **36**: 2109–16.

73 Sigurdsson S, Erlendsdóttir H, Quirk SJ, *et al.* Pneumococcal vaccination: Direct and herd effect on carriage of vaccine types and antibiotic resistance in Icelandic children. *Vaccine* 2017; **35**: 5242–8.

74 Sampane-Donkor E, Badoe EV, Annan JA, Nii-Trebi N. Colonisation of antibiotic resistant bacteria in a cohort of HIV infected children in Ghana. *Pan Afr Med J* 2017; **26**: 60.

75 Raman R, Sankar J, Putlibai S, Raghavan V. Demographic profile of healthy children with nasopharyngeal colonisation of Streptococcus pneumoniae: A research paper. *Indian J Med Microbiol* 2017; **35**: 607.

76 Neves FPG, Cardoso NT, Snyder RE, *et al.* Pneumococcal carriage among children after four years of routine 10-valent pneumococcal conjugate vaccine use in Brazil: The emergence of multidrug resistant serotype 6C. *Vaccine* 2017; **35**: 2794–800.

77 Nagaraj S, Kalal BS, Manoharan A, Shet A. Streptococcus pneumoniae serotype prevalence and antibiotic resistance among young children with invasive pneumococcal disease: experience from a tertiary care center in South India. *GERMS* 2017; **7**: 78–85.

78 Mayanskiy N, Savinova T, Alyabieva N, *et al.* Antimicrobial resistance, penicillin-binding protein sequences, and pilus islet carriage in relation to clonal evolution of Streptococcus pneumoniae serotype 19A in Russia, 2002–2013. *Epidemiol Infect* 2017; **145**: 1708–19.

79 Manoharan A, Manchanda V, Balasubramanian S, *et al.* Invasive pneumococcal disease in children aged younger than 5 years in India: a surveillance study. *Lancet Infect Dis* 2017; **17**: 305–12.

80 Lee JK, Yun KW, Choi EH, Kim SJ, Lee SY, Lee HJ. Changes in the Serotype Distribution among Antibiotic Resistant Carriage Streptococcus pneumoniae Isolates in Children after the

Introduction of the Extended-Valency Pneumococcal Conjugate Vaccine. *J Korean Med Sci* 2017; **32**: 1431.

81 Kobayashi M, Conklin LM, Bigogo G, *et al.* Pneumococcal carriage and antibiotic susceptibility patterns from two cross-sectional colonization surveys among children aged <5 years prior to the introduction of 10-valent pneumococcal conjugate vaccine — Kenya, 2009–2010. *BMC Infect Dis* 2017; **17**: 25.

82 Jiang H, Su M, Kui L, *et al.* Prevalence and antibiotic resistance profiles of cerebrospinal fluid pathogens in children with acute bacterial meningitis in Yunnan province, China, 2012-2015. *PLOS ONE* 2017; **12**: e0180161.

83 Houri H, Tabatabaei SR, Saee Y, Fallah F, Rahbar M, Karimi A. Distribution of capsular types and drug resistance patterns of invasive pediatric Streptococcus pneumoniae isolates in Teheran, Iran. *Int J Infect Dis* 2017; **57**: 21–6.

84 Gebre T, Tadesse M, Aragaw D, *et al.* Nasopharyngeal Carriage and Antimicrobial Susceptibility Patterns of Streptococcus pneumoniae among Children under Five in Southwest Ethiopia. *Children* 2017; **4**: 27.

85 Gaviria-Agudelo CL, Jordan-Villegas A, Garcia C, McCracken GH. The Effect of 13-Valent Pneumococcal Conjugate Vaccine on the Serotype Distribution and Antibiotic Resistance Profiles in Children With Invasive Pneumococcal Disease. *J Pediatr Infect Dis Soc* 2017; **6**: 253–9.

86 Cho Y-C, Chiu N-C, Lu C-Y, *et al.* Redistribution of Streptococcus pneumoniae Serotypes After Nationwide 13-valent Pneumococcal Conjugate Vaccine Program in Children in Northern Taiwan. *Pediatr Infect Dis J* 2017; **36**: e334–40.

87 Bloch EM, Mrango Z, Munoz B, *et al.* Antibiotic Resistance in Young Children in Kilosa District, Tanzania 4 Years after Mass Distribution of Azithromycin for Trachoma Control. *Am J Trop Med Hyg* 2017; **97**: 815–8.

88 Arvas A, Çokuğraş H, Gür E, Gönüllü N, Taner Z, Tokman HB. Pneumococcal Nasopharyngeal Carriage in Young Healthy Children After Pneumococcal Conjugate Vaccine in Turkey. *Balk Med J* 2017; published online March 9. DOI:10.4274/balkanmedj.2016.1256.

89 Ziane H, Manageiro V, Ferreira E, *et al.* Serotypes and Antibiotic Susceptibility of Streptococcus pneumoniae Isolates from Invasive Pneumococcal Disease and Asymptomatic Carriage in a Pre-vaccination Period, in Algeria. *Front Microbiol* 2016; **7**. DOI:10.3389/fmicb.2016.00803.

90 Wysocki J, Sluzewski W, Gutterman E, Jouve S, Moscariello M, Balter I. Active hospital-based surveillance of invasive pneumococcal disease and clinical pneumonia in infants and young children in two Polish counties. *Arch Med Sci* 2016; **3**: 629–38.

91 Tomczyk S, Lynfield R, Schaffner W, *et al.* Prevention of Antibiotic-Nonsusceptible Invasive Pneumococcal Disease With the 13-Valent Pneumococcal Conjugate Vaccine. *Clin Infect Dis* 2016; **62**: 1119–25.

92 Tantivitayakul P, Lapirattanakul J, Vichayanrat T, Muadchiengka T. Antibiotic Resistance Patterns and Related Mobile Genetic Elements of Pneumococci and β-Hemolytic Streptococci in Thai Healthy Children. *Indian J Microbiol* 2016; **56**: 417–25.

93 Swedan S, Hayajneh W, Bshara G. Genotyping and serotyping of macrolide and multidrug resistant Streptococcus pneumoniae isolated from carrier children. *Indian J Med Microbiol* 2016; **34**: 159.

94 Soysal A, Karabağ-Yılmaz E, Kepenekli E, *et al.* The impact of a pneumococcal conjugate vaccination program on the nasopharyngeal carriage, serotype distribution and antimicrobial resistance of Streptococcus pneumoniae among healthy children in Turkey. *Vaccine* 2016; **34**: 3894–900.

95 Soto-Noguerón A, Carnalla-Barajas MN, Solórzano-Santos F, *et al.* Streptococcus pneumoniae as cause of infection in infants less than 60 days of age: serotypes and antimicrobial susceptibility. *Int J Infect Dis IJID Off Publ Int Soc Infect Dis* 2016; **42**: 69–73.

96 Saha SK, Hossain B, Islam M, *et al.* Epidemiology of Invasive Pneumococcal Disease in Bangladeshi Children Before Introduction of Pneumococcal Conjugate Vaccine. *Pediatr Infect Dis J* 2016; **35**: 655–61.

97 Nunes S, Félix S, Valente C, *et al.* The impact of private use of PCV7 in 2009 and 2010 on serotypes and antimicrobial resistance of Streptococcus pneumoniae carried by young children in Portugal: Comparison with data obtained since 1996 generating a 15-year study prior to PCV13 introduction. *Vaccine* 2016; **34**: 1648–56.

98 Nor Azizah A, Fadzilah MN, Mariam M, *et al.* Community-acquired bacteremia in Paediatrics: Epidemiology, aetiology and patterns of antimicrobial resistance in a tertiary care centre, Malaysia. *Med J Malaysia* 2016; **71**: 117–21.

99 Nhantumbo AA, Gudo ES, Caierão J, *et al.* Serotype distribution and antimicrobial resistance of Streptococcus pneumoniae in children with acute bacterial meningitis in Mozambique: implications for a national immunization strategy. *BMC Microbiol* 2016; **16**: 134.

100 Moore CE, Giess A, Soeng S, *et al.* Characterisation of Invasive Streptococcus pneumoniae Isolated from Cambodian Children between 2007 – 2012. *PLoS ONE* 2016; **11**. DOI:10.1371/journal.pone.0159358.

101 Mokaddas E, Albert MJ. Serotype distribution and penicillin-non-susceptibility of Streptococcus pneumoniae causing invasive diseases in Kuwait: A 10-year study of impact of pneumococcal conjugate vaccines. *Expert Rev Vaccines* 2016; **15**: 1337–45.

102 Metcalf BJ, Gertz RE, Gladstone RA, *et al.* Strain features and distributions in pneumococci from children with invasive disease before and after 13-valent conjugate vaccine implementation in the USA. *Clin Microbiol Infect Off Publ Eur Soc Clin Microbiol Infect Dis* 2016; **22**: 60.e9-60.e29.

103 Menezes AP de O, Azevedo J, Leite MC, *et al.* Nasopharyngeal carriage of Streptococcus pneumoniae among children in an urban setting in Brazil prior to PCV10 introduction. *Vaccine* 2016; **34**: 791–7.

104 Lyu S, Yao K, Dong F, *et al.* Vaccine Serotypes of Streptococcus pneumoniae with High-level Antibiotic Resistance Isolated More Frequently Seven Years After the Licensure of PCV7 in Beijing. *Pediatr Infect Dis J* 2016; **35**: 316–21.

105 Lindstrand A, Galanis I, Darenberg J, *et al.* Unaltered pneumococcal carriage prevalence due to expansion of non-vaccine types of low invasive potential 8years after vaccine introduction in Stockholm, Sweden. *Vaccine* 2016; **34**: 4565–71.

106 Kwambana-Adams BA, Asiedu-Bekoe F, Sarkodie B, *et al.* An outbreak of pneumococcal meningitis among older children (≥5 years) and adults after the implementation of an infant vaccination programme with the 13-valent pneumococcal conjugate vaccine in Ghana. *BMC Infect Dis* 2016; **16**: 575.

107 Kaur R, Casey JR, Pichichero ME. Emerging Streptococcus pneumoniae Strains Colonizing the Nasopharynx in Children After 13-valent Pneumococcal Conjugate Vaccination in Comparison to the 7-valent Era, 2006–2015: *Pediatr Infect Dis J* 2016; **35**: 901–6.

108 Janoir C, Lepoutre A, Gutmann L, Varon E. Insight Into Resistance Phenotypes of Emergent Non 13-valent Pneumococcal Conjugate Vaccine Type Pneumococci Isolated From Invasive Disease After 13-valent Pneumococcal Conjugate Vaccine Implementation in France. *Open Forum Infect Dis* 2016; **3**: ofw020.

109 Hu J, Sun X, Huang Z, *et al.* Streptococcus pneumoniae and Haemophilus influenzae type b carriage in Chinese children aged 12–18 months in Shanghai, China: a cross-sectional study. *BMC Infect Dis* 2016; **16**: 149.

110 Horna G, Molero ML, Benites L, *et al.* Oxacillin disk diffusion testing for the prediction of penicillin resistance in Streptococcus pneumoniae. *Rev Panam Salud Publica Pan Am J Public Health* 2016; **40**: 57–63.

111 Hanke CR, Grijalva CG, Chochua S, *et al.* Bacterial Density, Serotype Distribution and Antibiotic Resistance of Pneumococcal Strains from the Nasopharynx of Peruvian Children Before and After Pneumococcal Conjugate Vaccine 7: *Pediatr Infect Dis J* 2016; **35**: 432–9.

112 Hadjipanayis A, Efstathiou E, Alexandrou M, *et al.* Nasopharyngeal Pneumococcal Carriage among Healthy Children in Cyprus Post Widespread Simultaneous Implementation of PCV10 and PCV13 Vaccines. *PLOS ONE* 2016; **11**: e0163269.

113 Hadinegoro SR, Prayitno A, Khoeri MM, *et al.* Nasopharyngeal Carriage of Streptococcus Pneumoniae in Healthy Children Under Five Years Old in Centeral Lombok Regency, Indonesia. *Southeast Asian J Trop Med Public Health* 2016; **47**: 485–93.

114 Gopi T, Ranjith J, Anandan S, Balaji V. Epidemiological characterisation of Streptococcus pneumoniae from India using multilocus sequence typing. *Indian J Med Microbiol* 2016; **34**: 17.

115 Gharailoo Z, Mousavi SF, Halvani N, Feizabadi MM. Antimicrobial Resistant Pattern and Capsular Typing of Streptococcus Pneumoniae Isolated from Children in Sistan -Baluchestan. *Maedica* 2016; **11**: 203–7.

116 Dunne EM, Carville K, Riley TV, *et al.* Aboriginal and non-Aboriginal children in Western Australia carry different serotypes of pneumococci with different antimicrobial susceptibility profiles. *Pneumonia* 2016; **8**: 15.

117 Choe YJ, Lee HJ, Lee H, *et al.* Emergence of antibiotic-resistant non-vaccine serotype pneumococci in nasopharyngeal carriage in children after the use of extended-valency pneumococcal conjugate vaccines in Korea. *Vaccine* 2016; **34**: 4771–6.

118 Chan KCC, Subramanian R, Chong P, *et al.* Pneumococcal carriage in young children after introduction of PCV13 in Hong Kong. *Vaccine* 2016; **34**: 3867–74.

119 Almazrou Y, Shibl AM, Alkhlaif R, *et al.* Epidemiology of invasive pneumococcal disease in Saudi Arabian children younger than 5 years of age. *J Epidemiol Glob Health* 2015; **6**: 95.

120 Zhou X, Liu J, Zhang Z, Liu Y, Wang Y, Liu Y. Molecular characteristics of penicillin-binding protein 2b, 2x and 1a sequences in Streptococcus pneumoniae isolates causing invasive diseases among children in Northeast China. *Eur J Clin Microbiol Infect Dis* 2016; **35**: 633–45.

121 Miernyk KM, Bulkow LR, Case SL, *et al.* Population structure of invasive Streptococcus pneumoniae isolates among Alaskan children in the conjugate vaccine era, 2001 to 2013. *Diagn Microbiol Infect Dis* 2016; **86**: 224–30.

122 Nakano S, Fujisawa T, Ito Y, *et al.* Serotypes, antimicrobial susceptibility, and molecular epidemiology of invasive and non-invasive Streptococcus pneumoniae isolates in paediatric patients after the introduction of 13-valent conjugate vaccine in a nationwide surveillance study conducted in Japan in 2012–2014. *Vaccine* 2016; **34**: 67–76.

123 Kang L-H, Liu M-J, Xu W-C, *et al.* Molecular epidemiology of pneumococcal isolates from children in China. *Saudi Med J* 2016; **37**: 403–13.

124 Ceyhan M, Ozsurekci Y, Gürler N, *et al.* Serotype distribution of Streptococcus pneumoniae in children with invasive diseases in Turkey: 2008–2014. *Hum Vaccines Immunother* 2016; **12**: 308–13.

125 Zhou JY, Isaacson-Schmid M, Utterson EC, *et al.* Prevalence of nasopharyngeal pneumococcal colonization in children and antimicrobial susceptibility profiles of carriage isolates. *Int J Infect Dis* 2015; **39**: 50–2.

126 Tóthpál A, Kardos S, Laub K, *et al.* Radical serotype rearrangement of carried pneumococci in the first 3 years after intensive vaccination started in Hungary. *Eur J Pediatr* 2015; **174**: 373–81.

127 Thummeepak R, Leerach N, Kunthalert D, Tangchaisuriya U, Thanwisai A, Sitthisak S. High prevalence of multi-drug resistant Streptococcus pneumoniae among healthy children in Thailand. *J Infect Public Health* 2015; **8**: 274–81.

128 Steens A, Caugant DA, Aaberge IS, Vestrheim DF. Decreased Carriage and Genetic Shifts in the Streptococcus pneumoniae Population After Changing the Seven-valent to the Thirteen-valent Pneumococcal Vaccine in Norway: *Pediatr Infect Dis J* 2015; **34**: 875–83.

129 Rutebemberwa E, Mpeka B, Pariyo G, *et al.* High prevalence of antibiotic resistance in nasopharyngeal bacterial isolates from healthy children in rural Uganda: A cross-sectional study. *Ups J Med Sci* 2015; **120**: 249–56.

130 Ramdani-Bouguessa N, Ziane H, Bekhoucha S, *et al.* Evolution of antimicrobial resistance and serotype distribution of Streptococcus pneumoniae isolated from children with invasive and noninvasive pneumococcal diseases in Algeria from 2005 to 2012. *New Microbes New Infect* 2015; **6**: 42–8.

131 Pan F, Han L, Huang W, *et al.* Serotype Distribution, Antimicrobial Susceptibility, and Molecular Epidemiology of Streptococcus pneumoniae Isolated from Children in Shanghai, China. *PLOS ONE* 2015; **10**: e0142892.

132 Olarte L, Barson WJ, Barson RM, *et al.* Impact of the 13-Valent Pneumococcal Conjugate Vaccine on Pneumococcal Meningitis in US Children. *Clin Infect Dis* 2015; **61**: 767–75.

133 Mills RO, Twum-Danso K, Owusu-Agyei S, Donkor ES. Epidemiology of pneumococcal carriage in children under five years of age in Accra, Ghana. *Infect Dis* 2015; **47**: 326–31.

134 Luthander J, Bennet R, Giske CG, Nilsson A, Eriksson M. The aetiology of paediatric bloodstream infections changes after pneumococcal vaccination and group B streptococcusprophylaxis. *Acta Paediatr* 2015; **104**: 933–9.

135 Keenan JD, Klugman KP, McGee L, *et al.* Evidence for Clonal Expansion After Antibiotic Selection Pressure: Pneumococcal Multilocus Sequence Types Before and After Mass Azithromycin Treatments. *J Infect Dis* 2015; **211**: 988–94.

136 Iregbu K, Abdullahi N. Profiles of acute bacterial meningitis isolates in children in National Hospital, Abuja. *Niger Med J* 2015; **56**: 297.

137 Imöhl M, Reinert RR, van der Linden M. Antibiotic susceptibility rates of invasive pneumococci before and after the introduction of pneumococcal conjugate vaccination in Germany. *Int J Med Microbiol* 2015; **305**: 776–83.

138 Imöhl M, Möller J, Reinert RR, Perniciaro S, van der Linden M, Aktas O. Pneumococcal meningitis and vaccine effects in the era of conjugate vaccination: results of 20 years of nationwide surveillance in Germany. *BMC Infect Dis* 2015; **15**: 61.

139 Hsiao H-J, Wu C-T, Huang J-L, *et al.* Clinical features and outcomes of invasive pneumococcal disease in a pediatric intensive care unit. *BMC Pediatr* 2015; **15**: 85.

140 Ho P-L, Chiu SS, Law PY, Chan EL, Lai EL, Chow K-H. Increase in the nasopharyngeal carriage of non-vaccine serogroup 15 Streptococcus pneumoniae after introduction of children pneumococcal conjugate vaccination in Hong Kong. *Diagn Microbiol Infect Dis* 2015; **81**: 145–8.

141 Greenhill AR, Phuanukoonnon S, Michael A, *et al.* Streptococcus pneumoniae and Haemophilus influenzae in paediatric meningitis patients at Goroka General Hospital, Papua New Guinea: serotype distribution and antimicrobial susceptibility in the pre-vaccine era. *BMC Infect Dis* 2015; **15**: 485.

142 Gounder PP, Brewster M, Bruce MG, *et al.* Impact of the Pneumococcal Conjugate Vaccine and Antibiotic Use on Nasopharyngeal Colonization by Antibiotic Nonsusceptible Streptococcus pneumoniae, Alaska, 2000[FIGURE DASH]2010. *Pediatr Infect Dis J* 2015; **34**: 1223–9.

143 El-Nawawy AA, Hafez SF, Meheissen MA, Shahtout NM, Mohammed EE. Nasopharyngeal Carriage, Capsular and Molecular Serotyping and Antimicrobial Susceptibility of Streptococcus pneumoniae among Asymptomatic Healthy Children in Egypt. *J Trop Pediatr* 2015; : fmv060.

144 Dunais B, Bruno P, Touboul P, *et al.* Impact of the 13-valent Pneumococcal Conjugate Vaccine on Nasopharyngeal Carriage of Streptococcus pneumoniae Among Children Attending Group Daycare in Southeastern France: *Pediatr Infect Dis J* 2015; **34**: 286–8.

145 Draz IH, Halawa EF, Wahby G, Ismail DK, Meligy BS. Pneumococcal infection among hospitalized Egyptian children. *J Egypt Public Health Assoc* 2015; **90**: 52–7.

146 dos Santos MS, Azevedo J, Menezes AP de O, *et al.* Temporal trends and clonal diversity of penicillin non-susceptible pneumococci from meningitis cases from 1996 to 2012, in Salvador, Brazil. *BMC Infect Dis* 2015; **15**: 302.

147 Diawara I, Zerouali K, Katfy K, *et al.* Invasive pneumococcal disease among children younger than 5 years of age before and after introduction of pneumococcal conjugate vaccine in Casablanca, Morocco. *Int J Infect Dis* 2015; **40**: 95–101.

148 Daana M, Rahav G, Hamdan A, *et al.* Measuring the effects of pneumococcal conjugate vaccine (PCV7) on Streptococcus pneumoniae carriage and antibiotic resistance: The Palestinian-Israeli Collaborative Research (PICR). *Vaccine* 2015; **33**: 1021–6.

149 Bles P, de Mast Q, van der Gaast-de Jongh CE, *et al.* Antibiotic resistance of Streptococcus pneumoniae colonising the nasopharynx of HIV-exposed Tanzanian infants. *Trop Med Int Health* 2015; **20**: 1559–63.

150 Balaji V, Jayaraman R, Verghese V, Baliga P, Kurien T. Pneumococcal serotypes associated with invasive disease in under five children in India & implications for vaccine policy. *Indian J Med Res* 2015; **142**: 286.

151 Ba ID, Ba A, Faye PM, *et al.* Pediatric invasive pneumococcal disease in Senegal. *Médecine Mal Infect* 2015; **45**: 463–9.

152 Altun HU, Hascelik G, Gür D, Eser ÖK. Invasive pneumococci before the introduction of pneumococcal conjugate vaccine in Turkey: antimicrobial susceptibility, serotype distribution, and molecular identification of macrolide resistance. *J Chemother* 2015; **27**: 74–9.

153 Ahl J, Melander E, Odenholt I, *et al.* Prevalence of penicillin-non-susceptible Streptococcus pneumoniae in children in day-care centres subjected to an intervention to prevent dispersion. *Infect Dis* 2015; **47**: 338–44.

154 Tóthpál A, Laub K, Kardos S, *et al.* Epidemiological analysis of pneumococcal serotype 19A in healthy children following PCV7 vaccination. *Epidemiol Infect* 2016; **144**: 1563–73.

155 Mijac V, Opavski N, Markovic M, *et al.* Trends in macrolide resistance of respiratory tract pathogens in the paediatric population in Serbia from 2004 to 2009. *Epidemiol Infect* 2015; **143**: 648–52.

156 Deng X, Arya G, Memari N, *et al.* Genetic Analysis of Invasive Pneumococcal Isolates from Children in Ontario, Canada, 2007–2012. *Pediatr Infect Dis J* 2015; **34**: 594–8.

157 Dagan R, Juergens C, Trammel J, *et al.* Efficacy of 13-Valent Pneumococcal Conjugate Vaccine (PCV13) Versus That of 7-Valent PCV (PCV7) Against Nasopharyngeal Colonization of Antibiotic-Nonsusceptible Streptococcus pneumoniae. *J Infect Dis* 2015; **211**: 1144–53.

158 Yadhav M. Study of Bacterial Meningitis in Children Below 5 Years with Comparative Evaluation of Gram Staining, Culture and Bacterial Antigen Detection. *J Clin Diagn Res* 2014. DOI:10.7860/JCDR/2014/6767.4215.

159 von Mollendorf C, Cohen C, de Gouveia L, *et al.* Factors Associated with Ceftriaxone Nonsusceptibility of Streptococcus pneumoniae: Analysis of South African National Surveillance Data, 2003 to 2010. *Antimicrob Agents Chemother* 2014; **58**: 3293–305.

160 von Gottberg A, de Gouveia L, Tempia S, *et al.* Effects of Vaccination on Invasive Pneumococcal Disease in South Africa. *N Engl J Med* 2014; **371**: 1889–99.

161 Swann O, Everett DB, Furyk JS, *et al.* Bacterial Meningitis in Malawian Infants <2 Months of Age: Etiology and Susceptibility to World Health Organization First-Line Antibiotics. *Pediatr Infect Dis J* 2014; **33**: 560–5.

162 Safari D, Kurniati N, Waslia L, *et al.* Serotype Distribution and Antibiotic Susceptibility of Streptococcus pneumoniae Strains Carried by Children Infected with Human Immunodeficiency Virus. *PLoS ONE* 2014; **9**: e110526.

163 Rupa V, Isaac R, Jalagandeeswaran R, Manoharan A, Rebekah G. Epidemiology of nasopharyngeal colonization by S. pneumoniae in Indian infants in the first 2 years of life. *Int J Pediatr Otorhinolaryngol* 2014; **78**: 1701–6.

164 Phongsamart W, Srifeungfung S, Chatsuwan T, *et al.* Changing trends in serotype distribution and antimicrobial susceptibility of Streptococcus pneumoniae causing invasive diseases in Central Thailand, 2009–2012. *Hum Vaccines Immunother* 2014; **10**: 1866–73.

165 Paulke-Korinek M, Kollaritsch H, Kundi M, *et al.* Characteristics of invasive pneumococcal disease in hospitalized children in Austria. *Eur J Pediatr* 2014; **173**: 469–76.

166 Okade H, Funatsu T, Eto M, *et al.* Impact of the pneumococcal conjugate vaccine on serotype distribution and susceptibility trends of pediatric non-invasive Streptococcus pneumoniae isolates in Tokai, Japan over a 5-year period. *J Infect Chemother* 2014; **20**: 423–8.

167 Mirzaei Ghazikalayeh H, Moniri R, Moosavi SGA, Rezaei M, Yasini M, Valipour M. Serotyping, Antibiotic Susceptibility and Related Risk Factors Aspects of Nasopharyngeal Carriage of Streptococcus pneumoniae in Healthy School Students. *Iran J Public Health* 2014; **43**: 1284–90.

168 Mayanskiy N, Alyabieva N, Ponomarenko O, *et al.* Serotypes and antibiotic resistance of non-invasive Streptococcus pneumoniae circulating in pediatric hospitals in Moscow, Russia. *Int J Infect Dis* 2014; **20**: 58–62.

169 Luminos M, Dorobat O, Jugulete G, *et al.* Nasopharyngeal carriage of Streptococcus pneumoniae in Romanian children before the introduction of the pneumococcal conjugated vaccination into the national immunization programme: a national, multi-centre, cross-sectional observational study. *Int J Infect Dis* 2014; **29**: 169–73.

170 Tam P-YI, Madoff LC, Coombes B, Pelton SI. Invasive Pneumococcal Disease After Implementation of 13-Valent Conjugate Vaccine. *PEDIATRICS* 2014; **134**: 210–7.

171 Iroh Tam P-Y, Coombes B, Madoff L, Pelton SI. Severity of Invasive Pneumococcal Disease in Children Caused by Susceptible and Nonsusceptible Isolates. *Pediatr Infect Dis J* 2014; **33**: 1206–7.

172 Gudnason T, Hrafnkelsson B, Laxdal B, Kristinsson KG. Risk factors for nasopharyngeal carriage of Streptococcus pneumoniae and effects of a hygiene intervention: repeated cross-sectional cohort study at day care centres. *Scand J Infect Dis* 2014; **46**: 493–501.

173 Cho EY, Lee H, Choi EH, *et al.* Serotype distribution and antibiotic resistance of Streptococcus pneumoniae isolated from invasive infections after optional use of the 7-valent conjugate vaccine in Korea, 2006–2010. *Diagn Microbiol Infect Dis* 2014; **78**: 481–6.

174 Bayram N, Apa H, Gülfidan G, Günay İ, Ünal N, Devrim İ. Changing Patterns of Antimicrobial Susceptibility of Invasive Pneumococcal Diseases after Introduction of Pneumococcal Conjugate Vaccine. *Indian J Pediatr* 2014; **81**: 1124–5.

175 Ba F, Seck A, Bâ M, *et al.* Identifying an appropriate PCV for use in Senegal, recent insights concerning Streptococcus pneumoniaeNP carriage and IPD in Dakar. *BMC Infect Dis* 2014; **14**: 627.

176 Al-Sheikh YA, Gowda LK, Marie MAM, John J, Dabwan KHM, Cs P. Distribution of Serotypes and Antibiotic Susceptibility Patterns Among Invasive Pneumococcal Diseases in Saudi Arabia. *Ann Lab Med* 2014; **34**: 210.

177 Abdinia B, Ahangarzadeh Rezaee M, Abdoli Oskouie S. Etiology and Antimicrobial Resistance Patterns of Acute Bacterial Meningitis in Children: A 10-Year Referral Hospital-Based Study in Northwest Iran. *Iran Red Crescent Med J* 2014; **16**. DOI:10.5812/ircmj.17616.

178 Torres N, Velásquez R, Mercado EH, *et al.* [Antibiotic resistance of streptococcus pneumoniae among healthy nasopharyngeal carriers in seven regions of Peru]. *Rev Peru Med Exp Salud Publica* 2013; **30**: 575–82.

179 Zuccotti G, Mameli C, Daprai L, *et al.* Serotype distribution and antimicrobial susceptibilities of nasopharyngeal isolates of Streptococcus pneumoniae from healthy children in the 13-valent pneumococcal conjugate vaccine era. *Vaccine* 2014; **32**: 527–34.

180 Özdemir H, Çiftçi E, Durmaz R, *et al.* Nasopharyngeal carriage of Streptococcus pneumoniae in healthy Turkish children after the addition of PCV7 to the national vaccine schedule. *Eur J Pediatr* 2014; **173**: 313–20.

181 Lee GM, Kleinman K, Pelton SI, *et al.* Impact of 13-Valent Pneumococcal Conjugate Vaccination on Streptococcus pneumoniae Carriage in Young Children in Massachusetts. *J Pediatr Infect Dis Soc* 2014; **3**: 23–32.

182 Yatim MM, Masri SN, Desa MNM, Taib NM, Nordin SA, Jamal F. Determination of phenotypes and pneumococcal surface protein A family types of Streptococcus pneumoniae from Malaysian healthy children. *J Microbiol Immunol Infect* 2013; **46**: 180–6.

183 Warda K, Oufdou K, Zahlane K, Bouskraoui M. Antibiotic resistance and serotype distribution of nasopharyngeal isolates of Streptococcus pneumoniae from children in Marrakech region (Morocco). *J Infect Public Health* 2013; **6**: 473–81.

184 von Gottberg A, Cohen C, de Gouveia L, *et al.* Epidemiology of invasive pneumococcal disease in the pre-conjugate vaccine era: South Africa, 2003–2008. *Vaccine* 2013; **31**: 4200–8.

185 Skovbjerg S, Söderström A, Hynsjö L, Normark BH, Ekdahl K, Åhrén C. Low rate of pneumococci non-susceptible to penicillin in healthy Swedish toddlers. *Scand J Infect Dis* 2013; **45**: 279–84.

186 Sharma D, Baughman W, Holst A, *et al.* Pneumococcal Carriage and Invasive Disease in Children Before Introduction of the 13-valent Conjugate Vaccine: Comparison With the Era Before 7-valent Conjugate Vaccine. *Pediatr Infect Dis J* 2013; **32**: 196.

187 Parra EL, De La Hoz F, Díaz PL, Sanabria O, Realpe ME, Moreno J. Changes in Streptococcus pneumoniae serotype distribution in invasive disease and nasopharyngeal carriage after the heptavalent pneumococcal conjugate vaccine introduction in Bogotá, Colombia. *Vaccine* 2013; **31**: 4033–8.

188 Otsuka T, Chang B, Shirai T, *et al.* Individual Risk Factors Associated With Nasopharyngeal Colonization With Streptococcus pneumoniae and Haemophilus influenzae: A Japanese Birth Cohort Study. *Pediatr Infect Dis J* 2013; **32**: 709–14.

189 Niedzielski A, Korona-Glowniak I, Malm A. High prevalence of Streptococcus pneumoniae in adenoids and nasopharynx in preschool children with recurrent upper respiratory tract infections in Poland – distribution of serotypes and drug resistance patterns. *Med Sci Monit* 2013; **19**: 54–60.

190 Neves FPG, Pinto TCA, Corrêa MA, *et al.* Nasopharyngeal carriage, serotype distribution and antimicrobial resistance of Streptococcus pneumoniae among children from Brazil before the introduction of the 10-valent conjugate vaccine. *BMC Infect Dis* 2013; **13**: 318.

191 Nasereddin A, Shtayeh I, Ramlawi A, Salman N, Salem I, Abdeen Z. Streptococcus pneumoniae from Palestinian Nasopharyngeal Carriers: Serotype Distribution and Antimicrobial Resistance. *PLoS ONE* 2013; **8**: e82047.

192 Musiime V, Cook A, Bakeera-Kitaka S, *et al.* Bacteremia, Causative Agents and Antimicrobial Susceptibility among HIV-1 Infected Children on Antiretroviral Therapy in Uganda and Zimbabwe: *Pediatr Infect Dis J* 2013; : 1.

193 Morozumi M, Chiba N, Ubukata K, *et al.* Antibiotic susceptibility in relation to genotype of Streptococcus pneumoniae, Haemophilus influenzae, and Mycoplasma pneumoniae responsible for community-acquired pneumonia in children. *J Infect Chemother* 2013; **19**: 432–40.

194 Ma X, Zhao R, Ma Z, *et al.* Serotype Distribution and Antimicrobial Resistance of Streptococcus pneumoniae Isolates Causing Invasive Diseases from Shenzhen Children's Hospital. *PLoS ONE* 2013; **8**: e67507.

195 Ma X, Yao K, Xie G, *et al.* Characterization of erythromycin-resistant Streptococcus pneumoniae isolates causing invasive diseases in Chinese children. *Chin Med J (Engl)* 2013; **126**: 1522–7.

196 Liu C, Xiong X, Xu W, Sun J, Wang L, Li J. Serotypes and Patterns of Antibiotic Resistance in Strains Causing Invasive Pneumococcal Disease in Children Less than 5 Years of Age. *PLoS ONE* 2013; **8**: e54254.

197 Lee S, Kim J-H, Kim S-H, Park M, Bae S. Prevalent Multidrug-resistant Nonvaccine Serotypes in Pneumococcal Carriage of Healthy Korean Children Associated with the Low Coverage of the Seven-valent Pneumococcal Conjugate Vaccine. *Osong Public Health Res Perspect* 2013; **4**: 316–22.

198 Lee S, Bae S, Lee K-J, Yu J-Y, Kang Y. Changes in serotype prevalence and antimicrobial resistance among invasive Streptococcus pneumoniae isolates in Korea, 1996–2008. *J Med Microbiol* 2013; **62**: 1204–10.

199 Lee M-R, Chen C-M, Chuang T-Y, Huang Y-T, Hsueh P-R. Capsular serotypes and antimicrobial susceptibilities of Streptococcus pneumoniae causing invasive pneumococcal disease from 2009–2012 with an emphasis on serotype 19A in bacteraemic pneumonia and empyema and β-lactam resistance. *Int J Antimicrob Agents* 2013; **42**: 395–402.

200 Lee EK, Jun JK, Choi UY, Kwon H-J, Kim K-H, Kang JH. Nasopharyngeal Carriage Rate and Serotypes of Streptococcus pneumoniae and Antimicrobial Susceptibility in Healthy Korean Children Younger than 5 Years Old: Focus on Influence of Pneumococcal Conjugate Vaccination. *Infect Chemother* 2013; **45**: 76.

201 Lalitha MK, David T, Thomas K. Nasopharyngeal swabs of school children, useful in rapid assessment of community antimicrobial resistance patterns in Streptococcus pneumoniae and Haemophilus influenzae. *J Clin Epidemiol* 2013; **66**: 44–51.

202 Kumar KLR. Circulating Serotypes and Trends in Antibiotic Resistance of Invasive Streptococcus Pneumoniae from Children under Five in Bangalore. *J Clin Diagn Res* 2013. DOI:10.7860/JCDR/2013/6384.3741.

203 Korona-Glowniak I, Niedzielski A, Malm A, Niedzielska G. Serotypes and antibiotic resistance of Streptococcus pneumoniae from adenoids in preschool children with recurrent upper respiratory tract infections. *Pol J Microbiol* 2013; **62**: 385–90.

204 Kaplan SL, Barson WJ, Lin PL, *et al.* Early Trends for Invasive Pneumococcal Infections in Children After the Introduction of the 13-valent Pneumococcal Conjugate Vaccine: *Pediatr Infect Dis J* 2013; **32**: 203–7.

205 Hare KM, Singleton RJ, Grimwood K, *et al.* Longitudinal Nasopharyngeal Carriage and Antibiotic Resistance of Respiratory Bacteria in Indigenous Australian and Alaska Native Children with Bronchiectasis. *PLoS ONE* 2013; **8**: e70478.

206 Githii S, Revathi G, Muigai A, Kariuki S. Carriage rate and serotypes of Streptococcus pneumoniae amongst children in Thika Hospital, Kenya. *Afr J Lab Med* 2013; **2**: 5 pages.

207 dos Santos SR, Passadore LF, Takagi EH, *et al.* Serotype distribution of Streptococcus pneumoniae isolated from patients with invasive pneumococcal disease in Brazil before and after ten-pneumococcal conjugate vaccine implementation. *Vaccine* 2013; **31**: 6150–4.

208 Dayie NT, Arhin RE, Newman MJ, *et al.* Penicillin resistance and serotype distribution of Streptococcus pneumoniaein Ghanaian children less than six years of age. *BMC Infect Dis* 2013; **13**: 490.

209 Camilli R, Daprai L, Cavrini F, *et al.* Pneumococcal Carriage in Young Children One Year after Introduction of the 13-Valent Conjugate Vaccine in Italy. *PLoS ONE* 2013; **8**: e76309.

210 Bautista-Márquez A, Richardson V, Ortiz-Orozco O, *et al.* Prevalence of Pneumococcal Disease, Serotype Distribution, and Antimicrobial Susceptibility in Mexican Children Younger than 5 Years of Age. *Arch Med Res* 2013; **44**: 142–50.

211 Assefa A, Gelaw B, Shiferaw Y, Tigabu Z. Nasopharyngeal Carriage and Antimicrobial Susceptibility Pattern of Streptococcus Pneumoniae among Pediatric Outpatients at Gondar University Hospital, North West Ethiopia. *Pediatr Neonatol* 2013; **54**: 315–21.

212 Al-Waili BR, Al-Thawadi S, Al Hajjar S. Impact of the revised penicillin susceptibility breakpoints for Streptococcus pneumoniae on antimicrobial resistance rates of meningeal and non-meningeal pneumococcal strains. *Ann Saudi Med* 2013; **33**: 111–5.

213 Linden M van der, Winkel N, Küntzel S, *et al.* Epidemiology of Streptococcus pneumoniae Serogroup 6 Isolates from IPD in Children and Adults in Germany. *PLOS ONE* 2013; **8**: e60848.

214 van der Linden M, Reinert RR, Kern WV, Imöhl M. Epidemiology of serotype 19A isolates from invasive pneumococcal disease in German children. *BMC Infect Dis* 2013; **13**: 70.

215 Rolo D, Fenoll A, Fontanals D, *et al.* Serotype 5 Pneumococci Causing Invasive Pneumococcal Disease Outbreaks in Barcelona, Spain (1997 to 2011). *J Clin Microbiol* 2013; **51**: 3585–90.

216 Hulten KG, Kaplan SL, Lamberth LB, *et al.* Changes in Streptococcus pneumoniae Serotype 19A Invasive Infections in Children from 1993 to 2011. *J Clin Microbiol* 2013; **51**: 1294–7.

217 Sakata H. Susceptibility in Parenteral Antibiotics in Streptococcus pneumoniae Isolated from Children with Invasive Pneumococcal Disease. *Kansenshogaku Zasshi* 2013; **87**: 1–5.

218 Oftadeh S, Gidding HF, Gilbert GL. Laboratory surveillance of invasive pneumococcal disease in New South Wales, Australia, before and after introduction of 7-valent conjugate vaccine: reduced disease, but not antibiotic resistance rates. *Epidemiol Infect* 2013; **141**: 1797–806.

219 Wroe PC, Lee GM, Finkelstein JA, *et al.* Pneumococcal Carriage and Antibiotic Resistance in Young Children Before 13-valent Conjugate Vaccine. *Pediatr Infect Dis J* 2012; **31**: 249–54.

220 Tóthpál A, Kardos S, Hajdú E, Nagy K, Linden M, Dobay O. Nasal carriage of Streptococcus pneumoniae among Hungarian children before the wide use of the conjugate vaccine. *Acta Microbiol Immunol Hung* 2012; **59**: 107–18.

221 Shibl AM, Memish ZA, Al-Kattan KM. Antibiotic resistance and serotype distribution of invasive pneumococcal diseases before and after introduction of pneumococcal conjugate vaccine in the Kingdom of Saudi Arabia (KSA). *Vaccine* 2012; **30**: G32–6.

222 Riva E, Salvini F, Garlaschi ML, Radaelli G, Giovannini M. The status of invasive pneumococcal disease among children younger than 5 years of age in north-west Lombardy, Italy. *BMC Infect Dis* 2012; **12**: 106.

223 Regev-Yochay G, Abullaish I, Malley R, *et al.* Streptococcus pneumoniae Carriage in the Gaza Strip. *PLoS ONE* 2012; **7**: e35061.

224 Porat N, Benisty R, Trefler R, Givon-Lavi N, Dagan R. Clonal Distribution of Common Pneumococcal Serotypes Not Included in the 7-Valent Conjugate Vaccine (PCV7): Marked Differences between Two Ethnic Populations in Southern Israel. *J Clin Microbiol* 2012; **50**: 3472–7.

225 Nielsen MV, Sarpong N, Krumkamp R, *et al.* Incidence and Characteristics of Bacteremia among Children in Rural Ghana. *PLoS ONE* 2012; **7**: e44063.

226 Moyo SJ, Steinbakk M, Aboud S, *et al.* Penicillin resistance and serotype distribution of Streptococcus pneumoniae in nasopharyngeal carrier children under 5 years of age in Dar es Salaam, Tanzania. *J Med Microbiol* 2012; **61**: 952–9.

227 Korona-Glowniak I, Malm A. Characteristics of Streptococcus pneumoniae Strains Colonizing Upper Respiratory Tract of Healthy Preschool Children in Poland. *Sci World J* 2012; **2012**: 1–10.

228 Kateete DP, Kajumbula H, Kaddu-Mulindwa DH, Ssevviri AK. Nasopharyngeal carriage rate of Streptococcus pneumoniae in Ugandan children with Sickle Cell Disease. *BMC Res Notes* 2012; **5**: 28.

229 Hernandez-Bou S, Garcia-Garcia JJ, Gene A, Esteva C, del Amo E, Muñoz-Almagro C. Pneumococcal carriage in children attending a hospital outpatient clinic in the era of pneumococcal conjugate vaccines in Barcelona. *Diagn Microbiol Infect Dis* 2012; **74**: 258–62.

230 Hanna-Wakim R, Chehab H, Mahfouz I, *et al.* Epidemiologic characteristics, serotypes, and antimicrobial susceptibilities of invasive Streptococcus pneumoniae isolates in a nationwide surveillance study in Lebanon. *Vaccine* 2012; **30**: G11–7.

231 Grivea IN, Sourla A, Ntokou E, Chryssanthopoulou DC, Tsantouli AG, Syrogiannopoulos GA. Macrolide resistance determinants among Streptococcus pneumoniae isolates from carriers in Central Greece. *BMC Infect Dis* 2012; **12**: 255.

232 Gherardi G, D'Ambrosio F, Visaggio D, Dicuonzo G, Del Grosso M, Pantosti A. Serotype and Clonal Evolution of Penicillin-Nonsusceptible Invasive Streptococcus pneumoniae in the 7-Valent Pneumococcal Conjugate Vaccine Era in Italy. *Antimicrob Agents Chemother* 2012; **56**: 4965–8.

233 Elmdaghri N, Benbachir M, Belabbes H, Zaki B, Benzaid H. Changing epidemiology of pediatric Streptococcus pneumoniae isolates before vaccine introduction in Casablanca (Morocco). *Vaccine* 2012; **30**: G46–50.

234 El Mdaghri N, Jilali N, Belabbes H, Jouhadi Z, Lahssoune M, Zaid S. Epidemiological profile of invasive bacterial diseases in children in Casablanca, Morocco: antimicrobial susceptibilities and serotype distribution. *East Mediterr Health J* 2012; **18**: 1097–101.

235 Devi U, Ayyagari A, Devi KR, *et al.* Serotype distribution & sensitivity pattern of nasopharyngeal colonizing Streptococcus pneumoniae among rural children of eastern India. *Indian J Med Res* 2012; **136**: 495–8.

236 de Sevilla MF, García-García J-J, Esteva C, *et al.* Clinical Presentation of Invasive Pneumococcal Disease in Spain in the Era of Heptavalent Conjugate Vaccine: *Pediatr Infect Dis J* 2012; **31**: 124–8.

237 Cho EY, Kang HM, Lee J, Kang JH, Choi EH, Lee HJ. Changes in Serotype Distribution and Antibiotic Resistance of Nasopharyngeal Isolates of Streptococcus pneumoniae from Children in Korea, after Optional Use of the 7-Valent Conjugate Vaccine. *J Korean Med Sci* 2012; **27**: 716.

238 Charfi F, Smaoui H, Kechrid A. Non-susceptibility trends and serotype coverage by conjugate pneumococcal vaccines in a Tunisian paediatric population: A 10-year study. *Vaccine* 2012; **30**: G18–24.

239 Benavides JA, Ovalle OO, Salvador GR, Gray S, Isaacman D, Rodgers GL. Population-based surveillance for invasive pneumococcal disease and pneumonia in infants and young children in Bogotá, Colombia. *Vaccine* 2012; **30**: 5886–92.

240 Barroso DE, Godoy D, Castiñeiras TMPP, Tulenko MM, Rebelo MC, Harrison LH. β-lactam Resistance, Serotype Distribution, and Genotypes of Meningitis-causing Streptococcus pneumoniae, Rio de Janeiro, Brazil. *Pediatr Infect Dis J* 2012; **31**: 30–6.

241 Arguedas A, Abdelnour A, Soley C, *et al.* Prospective epidemiologic surveillance of invasive pneumococcal disease and pneumonia in children in San José, Costa Rica. *Vaccine* 2012; **30**: 2342–8.

242 Andrade AL, Oliveira R, Vieira MA, *et al.* Population-based surveillance for invasive pneumococcal disease and pneumonia in infants and young children in Goiânia, Brazil. *Vaccine* 2012; **30**: 1901–9.

243 Stacevičienė I, Petraitienė S, Vaičiūnienė D, Alasevičius T, Kirslienė J, Usonis V. Antibiotic resistance of Streptococcus pneumoniae, isolated from nasopharynx of preschool children with acute respiratory tract infection in Lithuania. *BMC Infect Dis* 2016; **16**: 216.

244 Tanaka J, Ishiwada N, Wada A, *et al.* Incidence of childhood pneumonia and serotype and sequence-type distribution in Streptococcus pneumonia isolates in Japan. *Epidemiol Infect* 2012; **140**: 1111–21.

245 Pourakbari B, Sadr A, Haghi Ashtiani MT, *et al.* Five-year evaluation of the antimicrobial susceptibility patterns of bacteria causing bloodstream infections in Iran. *J Infect Dev Ctries* 2011; **6**: 120–5.

246 Hampton LM, Farley MM, Schaffner W, *et al.* Prevention of Antibiotic-Nonsusceptible Streptococcus pneumoniae With Conjugate Vaccines. *J Infect Dis* 2012; **205**: 401–11.

247 Yoshioka CRM, Brandileone MCC, Ragazzi SB, *et al.* Analysis of invasive pneumonia-causing strains of Streptococcus pneumoniae: serotypes and antimicrobial susceptibility. *J Pediatr (Rio J)* 2011; **87**: 70–5.

248 Vasoo S, Singh K, Hsu LY, *et al.* Increasing antibiotic resistance in Streptococcus pneumoniae colonizing children attending day-care centres in Singapore: Singapore children pneumococcal carriage. *Respirology* 2011; **16**: 1241–8.

249 Tóthpál A, Ordas A, Hajdú E, *et al.* A marked shift in the serotypes of pneumococci isolated from healthy children in Szeged, Hungary, over a 6-year period. *Acta Microbiol Immunol Hung* 2011; **58**: 239–46.

250 Tarragó D, Aguilar L, García R, Gimenez M-J, Granizo J-J, Fenoll A. Evolution of Clonal and Susceptibility Profiles of Serotype 19A Streptococcus pneumoniae among Invasive Isolates from Children in Spain, 1990 to 2008. *Antimicrob Agents Chemother* 2011; **55**: 2297–302.

251 Sakata H. Invasive Streptococcus pneumoniae infections in children in Kamikawa and Soya subprefecture, Hokkaido, Japan, 2000–2010, before the introduction of the 7-valent pneumococcal conjugate vaccine. *J Infect Chemother* 2011; **17**: 799–802.

252 Quintero B, Araque M, van der Gaast-de Jongh C, *et al.* Epidemiology of Streptococcus pneumoniae and Staphylococcus aureus colonization in healthy Venezuelan children. *Eur J Clin Microbiol Infect Dis* 2011; **30**: 7–19.

253 Pimenta FC, Carvalho M d. GS, Gertz RE, *et al.* Serotype and genotype distributions of pneumococcal carriage isolates recovered from Brazilian children attending day-care centres. *J Med Microbiol* 2011; **60**: 1455–9.

254 Oishi T, Wada A, Chang B, Toyabe S, Uchiyama M. Serotyping and multilocus sequence typing of Streptococcus pneumoniae isolates from the blood and posterior nares of Japanese children prior to the introduction of 7-valent pneumococcal conjugate vaccine. *Jpn J Infect Dis* 2011; **64**: 341–4.

255 Kuo C-Y, Hwang K-P, Hsieh Y-C, *et al.* Nasopharyngeal carriage of Streptococcus pneumoniae in Taiwan before and after the introduction of a conjugate vaccine. *Vaccine* 2011; **29**: 5171–7.

256 Korona-Glowniak I, Niedzielski A, Malm A. Upper respiratory colonization by Streptococcus pneumoniae in healthy pre-school children in south-east Poland. *Int J Pediatr Otorhinolaryngol* 2011; **75**: 1529–34.

257 Kim K-H, Hong JY, Lee H, *et al.* Nasopharyngeal Pneumococcal Carriage of Children Attending Day Care Centers in Korea: Comparison between Children Immunized with 7-valent Pneumococcal Conjugate Vaccine and Non-immunized. *J Korean Med Sci* 2011; **26**: 184.

258 Kempf M, Baraduc R, Bonnabau H, *et al.* Epidemiology and Antimicrobial Resistance of Streptococcus pneumoniae in France in 2007: Data from the Pneumococcus Surveillance Network. *Microb Drug Resist* 2011; **17**: 31–6.

259 Kattan R, Abu Rayyan A, Zheiman I, *et al.* Serotype Distribution and Drug Resistance in Streptococcus pneumoniae , Palestinian Territories. *Emerg Infect Dis* 2011; **17**: 94–6.

260 Jourdain S, Smeesters PR, Denis O, *et al.* Differences in nasopharyngeal bacterial carriage in preschool children from different socio-economic origins. *Clin Microbiol Infect* 2011; **17**: 907–14.

261 Janapatla R-P, Chang H-J, Hsu M-H, Hsieh Y-C, Lin T-Y, Chiu C-H. Nasopharyngeal carriage of Streptococcus pneumoniae, Haemophilus influenzae, Moraxella catarrhalis, and Alloiococcus otitidis in young children in the era of pneumococcal immunization, Taiwan. *Scand J Infect Dis* 2011; **43**: 937–42.

262 Ho P-L, Chiu SS, Chan MY, Ang I, Chow K-H, Lau Y-L. Changes in nasopharyngeal carriage and serotype distribution of antibiotic-resistant Streptococcus pneumoniae before and after the introduction of 7-valent pneumococcal conjugate vaccine in Hong Kong. *Diagn Microbiol Infect Dis* 2011; **71**: 327–34.

263 Ho P-L, Chiu SS, Ang I, Lau Y-L. Serotypes and antimicrobial susceptibilities of invasive Streptococcus pneumoniae before and after introduction of 7-valent pneumococcal conjugate vaccine, Hong Kong, 1995-2009. *Vaccine* 2011; **29**: 3270–5.

264 Hashida K, Shiomori T, Hohchi N, *et al.* Nasopharyngeal Streptococcus pneumoniae carriage in Japanese children attending day-care centers. *Int J Pediatr Otorhinolaryngol* 2011; **75**: 664–9.

265 Grivea IN, Tsantouli AG, Michoula AN, Syrogiannopoulos GA. Dynamics of Streptococcus pneumoniae nasopharyngeal carriage with high heptavalent pneumococcal conjugate vaccine coverage in Central Greece. *Vaccine* 2011; **29**: 8882–7.

266 Grall N, Longo M, Hurmic O, *et al.* Epidemiology of Streptococcus pneumoniae in France before introduction of the PCV-13 vaccine. *Eur J Clin Microbiol Infect Dis* 2011; **30**: 1511–9.

267 Falup-Pecurariu O, Bleotu L, Zavarache C, *et al.* Streptococcus Pneumoniae Nasopharyngeal Colonization in Children in Brasov, Central Romania: High Antibiotic Resistance and Coverage by Conjugate Vaccines. *Pediatr Infect Dis J* 2011; **30**: 76–8.

268 Ercan TE, Severge B, Topkaya A, Ercan RG, Altınkaya N. Effect of the pneumococcal conjugate vaccine on pneumococcal carriage in Turkish children: Pneumococcal conjugate vaccine on carriage of S. pneumoniae. *Pediatr Int* 2011; **53**: 224–30.

269 Dunais B, Bruno-Bazureault P, Carsenti-Dellamonica H, Touboul P, Pradier C. A decade-long surveillance of nasopharyngeal colonisation with Streptococcus pneumoniae among children attending day-care centres in south-eastern France: 1999–2008. *Eur J Clin Microbiol Infect Dis* 2011; **30**: 837–43.

270 Crump JA, Ramadhani HO, Morrissey AB, *et al.* Invasive bacterial and fungal infections among hospitalized HIV-infected and HIV-uninfected children and infants in northern Tanzania: Paediatric invasive infections in Tanzania. *Trop Med Int Health* 2011; **16**: 830–7.

271 Cornick JE, Everett DB, Broughton C, *et al.* Invasive Streptococcus pneumoniae in Children, Malawi, 2004–2006. *Emerg Infect Dis* 2011; **17**: 1107–9.

272 Baş AY, Demirel N, Aydin M, Zenciroglu A, Tonbul A, Tanir G. Pneumococcal meningitis in the newborn period in a prevaccination era: a 10-year experience at a tertiary intensive care unit. *Turk J Pediatr* 2011; **53**: 142–8.

273 Alvares JR, Mantese OC, Paula A de, *et al.* Prevalence of pneumococcal serotypes and resistance to antimicrobial agents in patients with meningitis: ten-year analysis. *Braz J Infect Dis* 2011; **15**: 22–7.

274 Al Ayed MS, Hawan AA. Retrospective review of invasive pediatric pneumococcal diseases in a military hospital in the southern region of Saudi Arabia. *Ann Saudi Med* 2011; **31**: 469–72.

275 Yasin RMD, Zin NM, Hussin A, *et al.* Current trend of pneumococcal serotypes distribution and antibiotic susceptibility pattern in Malaysian hospitals. *Vaccine* 2011; **29**: 5688–93.

276 Picazo J, Ruiz-Contreras J, Hernandez B, *et al.* Clonal and clinical profile of Streptococcus pneumoniae serotype 19A causing pediatric invasive infections: a 2-year (2007-2009) laboratory-based surveillance in Madrid. *Vaccine* 2011; **29**: 1770–6.

277 Miller E, Andrews NJ, Waight PA, Slack MP, George RC. Herd immunity and serotype replacement 4 years after seven-valent pneumococcal conjugate vaccination in England and Wales: an observational cohort study. *Lancet Infect Dis* 2011; **11**: 760–8.

278 Bonofiglio L, Regueira M, Pace J, Corso A, García E, Mollerach M. Dissemination of an erythromycin-resistant penicillin-nonsusceptible Streptococcus pneumoniae Poland(6B)-20 clone in Argentina. *Microb Drug Resist Larchmt N* 2011; **17**: 75–81.

279 Bhattacharya SD, Niyogi SK, Bhattacharyya S, *et al.* High Rates of Colonization with Drug Resistant Hemophilus Influenzae Type B and Streptococcus Pneumoniae in Unvaccinated HIV Infected Children from West Bengal. *Indian J Pediatr* 2011; **78**: 423–9.

280 Arredondo-García JL, Calderón E, Echániz-Aviles G, Soto-Noguerón A, Arzate P, Amabile-Cuevas CF. Serotypes and antibiotic susceptibility of Streptococcus pneumoniae isolates causative of invasive diseases in Mexican children. *J Infect Dev Ctries* 2010; **5**: 119–22.

281 Xue L, Yao K, Xie G, *et al.* Serotype Distribution and Antimicrobial Resistance of Streptococcus pneumoniae Isolates That Cause Invasive Disease among Chinese Children. *Clin Infect Dis* 2010; **50**: 741–4.

282 Vestrheim DF, Hoiby EA, Aaberge IS, Caugant DA. Impact of a Pneumococcal Conjugate Vaccination Program on Carriage among Children in Norway. *Clin Vaccine Immunol* 2010; **17**: 325–34.

283 Vasoo S, Singh K, Chow C, Lin RTP, Hsu LY, Tambyah PA. Pneumococcal carriage and resistance in children attending day care centers in Singapore in an early era of PCV-7 uptake. *J Infect* 2010; **60**: 507–9.

284 van Gils EJM, Veenhoven RH, Hak E, *et al.* Pneumococcal conjugate vaccination and nasopharyngeal acquisition of pneumococcal serotype 19A strains. *JAMA* 2010; **304**: 1099–106.

285 Techasaensiri C, Messina AF, Katz K, Ahmad N, Huang R, McCracken GH. Epidemiology and Evolution of Invasive Pneumococcal Disease Caused by Multidrug Resistant Serotypes of 19A in the 8 Years After Implementation of Pneumococcal Conjugate Vaccine Immunization in Dallas, Texas: *Pediatr Infect Dis J* 2009; : 1.

286 Talbert AWA, Mwaniki M, Mwarumba S, Newton CRJC, Berkley JA. Invasive Bacterial Infections in Neonates and Young Infants Born Outside Hospital Admitted to a Rural Hospital in Kenya: *Pediatr Infect Dis J* 2010; **29**: 945–9.

287 Srifeungfung S, Tribuddharat C, Comerungsee S, *et al.* Serotype coverage of pneumococcal conjugate vaccine and drug susceptibility of Streptococcus pneumoniae isolated from invasive or non-invasive diseases in central Thailand, 2006–2009. *Vaccine* 2010; **28**: 3440–4.

288 Skalet AH, Cevallos V, Ayele B, *et al.* Antibiotic Selection Pressure and Macrolide Resistance in Nasopharyngeal Streptococcus pneumoniae: A Cluster-Randomized Clinical Trial. *PLoS Med* 2010; **7**: e1000377.

289 Simões AS, Sá-Leão R, Eleveld MJ, *et al.* Highly Penicillin-Resistant Multidrug-Resistant Pneumococcus-Like Strains Colonizing Children in Oeiras, Portugal: Genomic Characteristics and Implications for Surveillance. *J Clin Microbiol* 2010; **48**: 238–46.

290 Schwarz NG, Sarpong N, Hünger F, *et al.* Systemic bacteraemia in children presenting with clinical pneumonia and the impact of non-typhoid salmonella (NTS). *BMC Infect Dis* 2010; **10**: 319.

291 Rijal B, Tandukar S, Adhikari R, *et al.* Antimicrobial susceptibility pattern and serotyping of Streptococcus pneumoniae isolated from Kanti Children Hospital in Nepal. *Kathmandu Univ Med J* 1970; **8**: 164–8.

292 Ochoa TJ, Egoavil M, Castillo ME, *et al.* Invasive pneumococcal diseases among hospitalized children in Lima, Peru. *Rev Panam Salud Pública* 2010; **28**: 121–7.

293 Mwenya DM, Charalambous BM, Phillips PPJ, *et al.* Impact of Cotrimoxazole on Carriage and Antibiotic Resistance of Streptococcus pneumoniae and Haemophilus influenzae in HIV-Infected Children in Zambia. *Antimicrob Agents Chemother* 2010; **54**: 3756–62.

294 Matsumoto A, Hashimoto K, Ito M, *et al.* The trend of drug-resistant Streptococcus pneumoniae from nasopharynx of children. *J Infect Chemother* 2010; **16**: 255–9.

295 Janapatla R-P, Hsu M-H, Du J-F, Hsieh Y-C, Lin T-Y, Chiu C-H. Sequence types and Antimicrobial Susceptibility of Invasive Streptococcus Pneumoniae Isolate from a region wtih high antibiotic selective pressure and suboptimal vaccine coverage. *Pediatr Infect Dis J* 2010; **29**: 467–9.

296 Imöhl M, van der Linden M, Mutscher C, Reinert RR. Serotype distribution of invasive pneumococcal disease during the first 60 days of life. *Vaccine* 2010; **28**: 4758–62.

297 Imöhl M, Reinert RR, van der Linden M. Regional differences in serotype distribution, pneumococcal vaccine coverage, and antimicrobial resistance of invasive pneumococcal disease among German federal states. *Int J Med Microbiol* 2010; **300**: 237–47.

298 Imöhl M, René Reinert R, van der Linden M. Serotype-specific penicillin resistance of Streptococcus pneumoniae in Germany from 1992 to 2008. *Int J Med Microbiol* 2010; **300**: 324–30.

299 Imöhl M, Reinert R, Mutscher C, van der Linden M. Macrolide susceptibility and serotype specific macrolide resistance of invasive isolates of Streptococcus pneumoniae in Germany from 1992 to 2008. *BMC Microbiol* 2010; **10**: 299.

300 Hsu KK, Shea KM, Stevenson AE, Pelton SI. Changing Serotypes Causing Childhood Invasive Pneumococcal Disease: Massachusetts, 2001–2007. *Pediatr Infect Dis J* 2009; : 1.

301 Gómez-Barreto D, Espinosa-Monteros LE, López-Enríquez C, Jiménez-Rojas V, Rodríguez-Suárez R. Invasive pneumococcal disease in a third level pediatric hospital in Mexico City: epidemiology and mortality risk factors. *Salud Pública México* 2010; **52**: 391–7.

302 Franco CM, Andrade ALS, Andrade JG, *et al.* Survey of Nonsusceptible Nasopharyngeal Streptococcus Pneumoniae Isolates in Children Attending Day-Care centers in Brazil. *Pediatr Infect Dis J* 2010; **29**: 77–9.

303 Eun BW, Kim SJ, Cho EY, Lee J, Choi EH, Lee HJ. Genetic structure of Streptococcus pneumoniae isolated from children in a tertiary care university hospital, in Korea, 1995 to 2005. *Diagn Microbiol Infect Dis* 2010; **68**: 345–51.

304 Bettinger JA, Scheifele DW, Kellner JD, *et al.* The effect of routine vaccination on invasive pneumococcal infections in Canadian children, Immunization Monitoring Program, Active 2000–2007. *Vaccine* 2010; **28**: 2130–6.

305 Aguiar SI, Brito MJ, Gonçalo-Marques J, Melo-Cristino J, Ramirez M. Serotypes 1, 7F and 19A became the leading causes of pediatric invasive pneumococcal infections in Portugal after 7 years of heptavalent conjugate vaccine use. *Vaccine* 2010; **28**: 5167–73.

306 Liu S, Dong L, Yang J. [Clinical characteristics and antimicrobial resistance of invasive pneumococcal disease in children]. *Zhonghua Er Ke Za Zhi Chin J Pediatr* 2010; **48**: 95–9.

307 Borg MA, Tiemersma E, Scicluna E, *et al.* Prevalence of penicillin and erythromycin resistance among invasive Streptococcus pneumoniae isolates reported by laboratories in the southern and eastern Mediterranean region. *Clin Microbiol Infect Off Publ Eur Soc Clin Microbiol Infect Dis* 2009; **15**: 232–7.

308 Rodrigues F, Nunes S, Sá-Leão R, Gonçalves G, Lemos L, de Lencastre H. Streptococcus pneumoniae nasopharyngeal carriage in children attending day-care centers in the central region of Portugal, in the era of 7-valent pneumococcal conjugate vaccine. *Microb Drug Resist Larchmt N* 2009; **15**: 269–77.

309 Karnezis TT, Smith A, Whittier S, Haddad J, Saiman L. Antimicrobial resistance among isolates causing invasive pneumococcal disease before and after licensure of heptavalent conjugate pneumococcal vaccine. *PloS One* 2009; **4**: e5965.

310 C. Bere L, Simpore J, D. Karou S, *et al.* Antimicrobial Resistance and Serotype Distribution of Streptococcus pneumoniae Strains Causing Childhood Infection in Burkina Faso. *Pak J Biol Sci* 2009; **12**: 1282–6.

311 Ndip RN, Ntiege EA, Ndip LM, Nkwelang G, Akoachere JFTK, Akenji T N. Antimicrobial resistance of bacterial agents of the upper respiratory tract of school children in Buea, Cameroon. *J Health Popul Nutr* 2008; **26**: 397–404.

312 Sá-Leão R, Nunes S, Brito-Avô A, *et al.* Changes in pneumococcal serotypes and antibiotypes carried by vaccinated and unvaccinated day-care centre attendees in Portugal, a country with widespread use of the seven-valent pneumococcal conjugate vaccine. *Clin Microbiol Infect Off Publ Eur Soc Clin Microbiol Infect Dis* 2009; **15**: 1002–7.

313 Velasquez PAG, Parussolo L, Cardoso CL, Tognim MCB, Garcia LB. High prevalence of children colonized with penicillin-resistant Streptococcus pneumoniae in public day-care centers. *J Pediatr (Rio J)* 2009; **85**: 516–22.

314 Arifeen SE, Saha SK, Rahman S, *et al.* Invasive pneumococcal disease among children in rural Bangladesh: results from a population-based surveillance. *Clin Infect Dis Off Publ Infect Dis Soc Am* 2009; **48 Suppl 2**: S103-113.

315 Falade AG, Lagunju IA, Bakare RA, Odekanmi AA, Adegbola RA. Invasive pneumococcal disease in children aged <5 years admitted to 3 urban hospitals in Ibadan, Nigeria. *Clin Infect Dis Off Publ Infect Dis Soc Am* 2009; **48 Suppl 2**: S190-196.

316 Pérez-Trallero E, Marimon JM, Ercibengoa M, Vicente D, Pérez-Yarza EG. Invasive Streptococcus pneumoniae infections in children and older adults in the north of Spain before and after the introduction of the heptavalent pneumococcal conjugate vaccine. *Eur J Clin Microbiol Infect Dis Off Publ Eur Soc Clin Microbiol* 2009; **28**: 731–8.

317 Castañeda E, Agudelo CI, Regueira M, *et al.* Laboratory-based surveillance of Streptococcus pneumoniae invasive disease in children in 10 Latin American countries: a SIREVA II project, 2000-2005. *Pediatr Infect Dis J* 2009; **28**: e265-270.

318 Grivea IN, Tsantouli AG, Chryssanthopoulou DC, Syrogiannopoulos GA. Interaction of the heptavalent pneumococcal conjugate vaccine and the use of individual antibiotics among children on nasopharyngeal colonization with erythromycin-resistant Streptococcus pneumoniae. *Eur J Clin Microbiol Infect Dis* 2010; **29**: 97–105.

319 Zaidi AKM, Khan H, Lasi R, Mahesar W, Sindh Meningitis Group. Surveillance of pneumococcal meningitis among children in Sindh, southern Pakistan. *Clin Infect Dis Off Publ Infect Dis Soc Am* 2009; **48 Suppl 2**: S129-135.

320 Saha SK, Naheed A, El Arifeen S, *et al.* Surveillance for invasive Streptococcus pneumoniae disease among hospitalized children in Bangladesh: antimicrobial susceptibility and serotype distribution. *Clin Infect Dis Off Publ Infect Dis Soc Am* 2009; **48 Suppl 2**: S75-81.

321 Katsarolis I, Poulakou G, Analitis A, *et al.* Risk factors for nasopharyngeal carriage of drug-resistant Streptococcus pneumoniae: data from a nation-wide surveillance study in Greece. *BMC Infect Dis* 2009; **9**: 120.

322 Otsuka T, Ono T, Okazaki M. Resistance of nasopharyngeal pathogens and antimicrobial prescription rates for children in an area under controlled antimicrobial use. *Pediatr Infect Dis J* 2009; **28**: 128–30.

323 Mantese OC, Paula A de, Almeida VVP, *et al.* Prevalence of serotypes and antimicrobial resistance of invasive strains of pneumococcus in children: analysis of 9 years. *J Pediatr (Rio J)* 2009; **85**: 495–502.

324 Corso A, Faccone D, Gagetti P, *et al.* Prevalence of mef and ermB genes in invasive pediatric erythromycin-resistant Streptococcus pneumoniae isolates from Argentina. *Rev Argent Microbiol* 2009; **41**: 29–33.

325 Cekmez F, Karademir F, Tanju IA, *et al.* Pneumococcal serotypes recovered from health children and their possible association with risk factor in istanbul, Turkey. *Int J Biomed Sci IJBS* 2009; **5**: 101–4.

326 Dortet L, Ploy M-C, Poyart C, Raymond J, ORP Ile de France Ouest. Emergence of Streptococcus pneumoniae of serotype 19A in France: molecular capsular serotyping, antimicrobial susceptibilities, and epidemiology. *Diagn Microbiol Infect Dis* 2009; **65**: 49–57.

327 Rendi-Wagner P, Paulke-Korinek M, Kundi M, *et al.* National paediatric immunization program of high risk groups: no effect on the incidence of invasive pneumococcal diseases. *Vaccine* 2009; **27**: 3963–8.

328 Torun MM, Namal N, Demirci M, Bahar H. Nasopharyngeal carriage and antibiotic resistance of Haemophilus influenzae, Streptococcus pneumoniae and Moraxella catarrhalis in healthy school children in Turkey. *Indian J Med Microbiol* 2009; **27**: 86–8.

329 Leach AJ, Morris PS, McCallum GB, *et al.* Emerging pneumococcal carriage serotypes in a high-risk population receiving universal 7-valent pneumococcal conjugate vaccine and 23-valent polysaccharide vaccine since 2001. *BMC Infect Dis* 2009; **9**: 121.

330 Greenberg D, Givon-Lavi N, Sharf AZ, Vardy D, Dagan R. The association between antibiotic use in the community and nasopharyngeal carriage of antibiotic-resistant Streptococcus pneumoniae in Bedouin children. *Pediatr Infect Dis J* 2008; **27**: 776–82.

331 Sánchez-Tatay D, Arroyo LA, Tarragó D, *et al.* Antibiotic susceptibility and molecular epidemiology of nasopharyngeal pneumococci from Spanish children. *Clin Microbiol Infect Off Publ Eur Soc Clin Microbiol Infect Dis* 2008; **14**: 797–801.

332 Arnason S, Thors VS, Gudnason T, Kristinsson KG, Haraldsson A. [Bacteraemia in children in Iceland 1994-2005]. *Laeknabladid* 2008; **94**: 523–9.

333 Nantanda R, Hildenwall H, Peterson S, Kaddu-Mulindwa D, Kalyesubula I, Tumwine JK. Bacterial aetiology and outcome in children with severe pneumonia in Uganda. *Ann Trop Paediatr* 2008; **28**: 253–60.

334 Shibl AM. Distribution of serotypes and antibiotic resistance of invasive pneumococcal disease isolates among children aged 5 years and under in Saudi Arabia (2000-2004). *Clin Microbiol Infect Off Publ Eur Soc Clin Microbiol Infect Dis* 2008; **14**: 876–9.

335 Mokaddas EM, Rotimi VO, Albert MJ. Implications of Streptococcus pneumoniae penicillin resistance and serotype distribution in Kuwait for disease treatment and prevention. *Clin Vaccine Immunol CVI* 2008; **15**: 203–7.

336 Munoz-Almagro C, Jordan I, Gene A, Latorre C, Garcia-Garcia JJ, Pallares R. Emergence of Invasive Pneumococcal Disease Caused by Nonvaccine Serotypes in the Era of 7-Valent Conjugate Vaccine. *Clin Infect Dis* 2008; **46**: 174–82.

337 Pérez A, Herranz M, Segura M, *et al.* Epidemiologic impact of blood culture practices and antibiotic consumption on pneumococcal bacteraemia in children. *Eur J Clin Microbiol Infect Dis Off Publ Eur Soc Clin Microbiol* 2008; **27**: 717–24.

338 Winters M, Patrick DM, Marra F, *et al.* Epidemiology of invasive pneumococcal disease in BC during the introduction of conjugated pneumococcal vaccine. *Can J Public Health Rev Can Sante Publique* 2008; **99**: 57–61.

339 Daikos GL, Koutsolioutsou A, Tsiodras S, *et al.* Evolution of macrolide resistance in Streptococcus pneumoniae clinical isolates in the prevaccine era. *Diagn Microbiol Infect Dis* 2008; **60**: 393–8.

340 Dueger EL, Asturias EJ, Matheu J, Gordillo R, Torres O, Halsey N. Increasing penicillin and trimethoprim-sulfamethoxazole resistance in nasopharyngeal Streptococcus pneumoniae isolates from Guatemalan children, 2001--2006. *Int J Infect Dis IJID Off Publ Int Soc Infect Dis* 2008; **12**: 289–97.

341 Brauteset LV, Høiby EA, Syversen G, Surén P, Wathne K-O. [Invasive pneumoccal disease in children in Oslo 1998-2004]. *Tidsskr Den Nor Laegeforening Tidsskr Prakt Med Ny Raekke* 2008; **128**: 1380–3.

342 Heffernan HM, Martin DR, Woodhouse RE, Morgan J, Blackmore TK. Invasive pneumococcal disease in New Zealand 1998-2005: capsular serotypes and antimicrobial resistance. *Epidemiol Infect* 2008; **136**: 352–9.

343 Chong C-Y, Koh-Cheng T, Yee-Hui M, Nancy TW-S. Invasive pneumococcal disease in Singapore children. *Vaccine* 2008; **26**: 3427–31.

344 Sombrero L, Nissinen A, Esparar G, *et al.* Low incidence of antibiotic resistance among invasive and nasopharyngeal isolates of Streptococcus pneumoniae from children in rural Philippines between 1994 and 2000. *Eur J Clin Microbiol Infect Dis Off Publ Eur Soc Clin Microbiol* 2008; **27**: 929–35.

345 Pírez García MC, Giachetto Larraz G, Romero Rostagno C, *et al.* Neumonía neumocócica invasiva en niños de 0 a 24 meses: ¿influye la resistencia bacteriana en la evolución? *An Pediatría* 2008; **69**: 205–9.

346 Kastrin T, Gubina M, Paragi M, *et al.* Macrolide resistance among invasive Streptococcus pneumoniae in Slovenia. *J Antimicrob Chemother* 2008; **62**: 628–9.

347 Cardoso MRA, Nascimento-Carvalho CM, Ferrero F, *et al.* Penicillin-resistant pneumococcus and risk of treatment failure in pneumonia. *Arch Dis Child* 2008; **93**: 221–5.

348 Steenhoff AP, Wood SM, Rutstein RM, Wahl A, McGowan KL, Shah SS. Invasive pneumococcal disease among human immunodeficiency virus-infected children, 1989-2006. *Pediatr Infect Dis J* 2008; **27**: 886–91.

349 Vestrheim DF, Høiby EA, Aaberge IS, Caugant DA. Phenotypic and genotypic characterization of Streptococcus pneumoniae strains colonizing children attending day-care centers in Norway. *J Clin Microbiol* 2008; **46**: 2508–18.

350 Dunais B, Bruno P, Carsenti-Dellamonica H, Touboul P, Dellamonica P, Pradier C. Trends in nasopharyngeal carriage of Streptococcus pneumoniae among children attending daycare centers in southeastern France from 1999 to 2006. *Pediatr Infect Dis J* 2008; **27**: 1033–5.

351 Ishiwada N, Kurosaki T, Terashima I, Kohno Y. The incidence of pediatric invasive pneumococcal disease in Chiba prefecture, Japan (2003-2005). *J Infect* 2008; **57**: 455–8.

352 Guzvinec M, Tesović G, Tambić-Andrasević A, Zidovec-Lepej S, Vukić BT, Begovac J. The epidemiology of invasive Streptococcus pneumoniae disease in Croatian children. *Med Sci Monit Int Med J Exp Clin Res* 2008; **14**: PH59-64.

353 Abut LI, Apan T, Otlu B, Calişkan A, Durmaz R. The characteristics of nasopharyngeal Streptococcus pneumoniae in children attending a daycare unit. *New Microbiol* 2008; **31**: 357–62.

354 Villaseñor-Sierra A, Lomas-Bautista M, Aguilar-Benavides S, Martínez-Aguilar G. Serotypes and susceptibility of Streptococcus pneumoniae strains isolated from children in Mexico. *Salud Publica Mex* 2008; **50**: 330–3.

355 Liu Y, Wang H, Chen M, *et al.* Serotype distribution and antimicrobial resistance patterns of Streptococcus pneumoniae isolated from children in China younger than 5 years. *Diagn Microbiol Infect Dis* 2008; **61**: 256–63.

356 Montagnani F, Fanetti A, Stolzuoli L, *et al.* Pneumococcal disease in a paediatric population in a hospital of central Italy: a clinical and microbiological case series from 1992 to 2006. *J Infect* 2008; **56**: 179–84.

357 Ozdemir B, Beyazova U, Camurdan AD, Sultan N, Ozkan S, Sahin F. Nasopharyngeal carriage of Streptococcus pneumoniae in healthy Turkish infants. *J Infect* 2008; **56**: 332–9.

358 Bayer M, Aslan G, Emekdaş G, Kuyucu N, Kanik A. [Nasopharyngeal carriage of Streptococcus pneumoniae in healthy children and multidrug resistance]. *Mikrobiyol Bul* 2008; **42**: 223–30.

359 Grivea IN, Panagiotou M, Tsantouli AG, Syrogiannopoulos GA. Impact of heptavalent pneumococcal conjugate vaccine on nasopharyngeal carriage of penicillin-resistant Streptococcus pneumoniae among day-care center attendees in central Greece. *Pediatr Infect Dis J* 2008; **27**: 519–25.

360 Sigaúque B, Roca A, Sanz S, *et al.* Acute bacterial meningitis among children, in Manhiça, a rural area in Southern Mozambique. *Acta Trop* 2008; **105**: 21–7.

361 Park SY, Moore MR, Bruden DL, *et al.* Impact of conjugate vaccine on transmission of antimicrobial-resistant Streptococcus pneumoniae among Alaskan children. *Pediatr Infect Dis J* 2008; **27**: 335–40.

362 Dias R, Caniça M. Invasive pneumococcal disease in Portugal prior to and after the introduction of pneumococcal heptavalent conjugate vaccine. *FEMS Immunol Med Microbiol* 2007; **51**: 35–42.

363 Rivera-Olivero IA, Bogaert D, Bello T, *et al.* CSE Global Theme Issue on Poverty and Human Development Pneumococcal Carriage among Indigenous Warao Children in Venezuela: Serotypes, Susceptibility Patterns, and Molecular Epidemiology. *Clin Infect Dis* 2007; **45**: 1427–34.

364 Messina AF, Katz-Gaynor K, Barton T, *et al.* Impact of the pneumococcal conjugate vaccine on serotype distribution and antimicrobial resistance of invasive Streptococcus pneumoniae isolates in Dallas, TX, children from 1999 through 2005. *Pediatr Infect Dis J* 2007; **26**: 461–7.

365 Members of the Canadian Paediatric Society's Immunization Monitoring Program, Active (IMPACT), Bettinger JA, Scheifele DW, Halperin SA, Kellner JD, Tyrrell G. Invasive Pneumococcal Infections in Canadian Children, 1998–2003 Implications for New Vaccination Programs. *Can J Public Health* 2007; **98**: 111–5.

366 Chen C-J, Huang Y-C, Su L-H, Lin T-Y. Nasal carriage of Streptococcus pneumoniae in healthy children and adults in northern Taiwan. *Diagn Microbiol Infect Dis* 2007; **59**: 265–9.

367 Uzuner A, Ilki A, Akman M, *et al.* Nasopharyngeal carriage of penicillin-resistant Streptococcus pneumoniae in healthy children. *Turk J Pediatr* 2007; **49**: 370–8.

368 Wattal C, Oberoi JK, Pruthi PK, Gupta S. Nasopharyngeal carriage of Streptococcus pneumoniae. *Indian J Pediatr* 2007; **74**: 905–7.

369 Nyandiko WM, Greenberg D, Shany E, Yiannoutsos CT, Musick B, Mwangi AW. Nasopharyngeal Streptococcus pneumoniae among under-five year old children at the Moi Teaching and Referral Hospital, Eldoret, Kenya. *East Afr Med J* 2007; **84**: 156–62.

370 Poulakou G, Katsarolis I, Matthaiopoulou I, *et al.* Nationwide surveillance of Streptococcus pneumoniae in Greece: patterns of resistance and serotype epidemiology. *Int J Antimicrob Agents* 2007; **30**: 87–92.

371 Pagliano P, Fusco U, Attanasio V, *et al.* Pneumococcal meningitis in childhood: a longitudinal prospective study. *FEMS Immunol Med Microbiol* 2007; **51**: 488–95.

372 Blossom DB, Cordeiro SM, Bajaksouzian S, *et al.* Characterization of penicillin intermediate serotypes of Streptococcus pneumoniae carried by human immunodeficiency virus-infected adults and healthy children in Uganda. *Microb Drug Resist Larchmt N* 2007; **13**: 21–8.

373 Tamm E, Naaber P, Maimets M, Oona M, Kõljalg S, Lutsar I. Antimicrobial susceptibility and serogroup/serotype distribution of nasopharyngeal isolates of Streptococcus pneumoniae in healthy Estonian children in 1999–2003. *Clin Microbiol Infect* 2007; **13**: 824–6.

374 Reinert RR, van der Linden M, Seegmüller I, *et al.* Molecular epidemiology of penicillin-non-susceptible Streptococcus pneumoniae isolates from children with invasive pneumococcal disease in Germany. *Clin Microbiol Infect Off Publ Eur Soc Clin Microbiol Infect Dis* 2007; **13**: 363–8.

375 Brooks WA, Breiman RF, Goswami D, *et al.* Invasive pneumococcal disease burden and implications for vaccine policy in urban Bangladesh. *Am J Trop Med Hyg* 2007; **77**: 795–801.

376 Roche A, Heath PT, Sharland M, *et al.* Prevalence of nasopharyngeal carriage of pneumococcus in preschool children attending day care in London. *Arch Dis Child* 2007; **92**: 1073–6.

377 Aristegui J, Bernaola E, Pocheville I, *et al.* Reduction in pediatric invasive pneumococcal disease in the Basque Country and Navarre, Spain, after introduction of the heptavalent pneumococcal conjugate vaccine. *Eur J Clin Microbiol Infect Dis Off Publ Eur Soc Clin Microbiol* 2007; **26**: 303–10.

378 Aslan G, Emekdas G, Bayer M, Serin MS, Kuyucu N, Kanik A. Serotype distribution of Streptococcus pneumoniae strains in the nasopharynx of healthy Turkish children. *Indian J Med Res* 2007; **125**: 582–7.

379 Espinosa-de Los Monteros LE, Jiménez-Rojas V, Aguilar-Ituarte F, *et al.* Streptococcus pneumoniae isolates in healthy children attending day-care centers in 12 states in Mexico. *Salud Publica Mex* 2007; **49**: 249–55.

380 Katz A, Leibovitz E, Timchenko VN, *et al.* Antibiotic susceptibility, serotype distribution and vaccine coverage of nasopharyngeal and oropharyngeal Streptococcus pneumoniae in a day-care centre in St. Petersburg, Russia. *Scand J Infect Dis* 2007; **39**: 293–8.

381 Vieira AC, Gomes MC, Filho MR, Filho JE, Bello EJM, Figueiredo RB de. Streptococcus pneumoniae: a study of strains isolated from cerebrospinal fluid. *J Pediatr (Rio J)* 2007; **83**: 71–8.

382 Souli M, Volonakis K, Kapaskelis A, *et al.* Characterisation of macrolide-non-susceptible Streptococcus pneumoniae colonising children attending day-care centres in Athens, Greece during 2000 and 2003. *Clin Microbiol Infect* 2007; **13**: 70–7.

383 Schultsz C, Vien LM, Campbell JI, *et al.* Changes in the nasal carriage of drug-resistant Streptococcus pneumoniae in urban and rural Vietnamese schoolchildren. *Trans R Soc Trop Med Hyg* 2007; **101**: 484–92.

384 Phongsamart W, Srifeungfung S, Dejsirilert S, *et al.* Serotype distribution and antimicrobial susceptibility of S. pneumoniae causing invasive disease in Thai children younger than 5 years old, 2000–2005. *Vaccine* 2007; **25**: 1275–80.

385 Orrett FA, Changoor E. Bacteremia in children at a regional hospital in Trinidad. *Int J Infect Dis* 2007; **11**: 145–51.

386 Pineda V, Fontanals D, Larramona H, Domingo M, Anton J, Segura F. Epidemiology of invasive Streptococcus pneumoniae infections in children in an area of Barcelona, Spain. *Acta Paediatr* 2007; **91**: 1251–6.

387 Zanchi A, Montagnani F, Stolzuoli L, Cellesi C. Serotype distribution, clonality and antimicrobial resistance of invasive pneumococcal isolates in a central Italian region: implications for vaccine strategies. *Eur J Pediatr* 2007; **166**: 875–7.

388 Sakata H. Bactericidal activities of parenteral antibiotics and genotype of penicillin-binding protein in Streptococcus pneumoniae and Haemophilus influenzae isolated from children's blood. *J Infect Chemother* 2006; **12**: 338–42.

389 Whitney CG, Pilishvili T, Farley MM, *et al.* Effectiveness of seven-valent pneumococcal conjugate vaccine against invasive pneumococcal disease: a matched case-control study. *The Lancet* 2006; **368**: 1495–502.

390 Levidiotou S, Vrioni G, Tzanakaki G, *et al.* Serotype distribution of Streptococcus pneumoniae in north-western Greece and implications for a vaccination programme. *FEMS Immunol Med Microbiol* 2006; **48**: 179–82.

391 García-Suárez MDM, Villaverde R, Caldevilla AF, Méndez FJ, Vázquez F. Serotype distribution and antimicrobial resistance of invasive and non-invasive pneumococccal isolates in Asturias, Spain. *Jpn J Infect Dis* 2006; **59**: 299–205.

392 Arason VA, Sigurdsson JA, Erlendsdottir H, Gudmundsson S, Kristinsson KG. The Role of Antimicrobial Use in the Epidemiology of Resistant Pneumococci: A 10-Year Follow Up. *Microb Drug Resist* 2006; **12**: 169–76.

393 Volonakis K, Souli M, Kapaskelis A, *et al.* Evolution of resistance patterns and identification of risk factors for Streptococcus pneumoniae colonisation in daycare centre attendees in Athens, Greece. *Int J Antimicrob Agents* 2006; **28**: 297–301.

394 Roca A, Sigauque B, Quinto Ll, *et al.* Invasive pneumococcal disease in children <5 years of age in rural Mozambique. *Trop Med Int Health* 2006; **11**: 1422–31.

395 Russell FM, Carapetis JR, Ketaiwai S, *et al.* Pneumococcal nasopharyngeal carriage and patterns of penicillin resistance in young children in Fiji. *Ann Trop Paediatr* 2006; **26**: 187–97.

396 Tarallo L, Tancredi F, Schito G, Marchese A, Bella A. Active surveillance of Streptococcus pneumoniae bacteremia in Italian children. *Vaccine* 2006; **24**: 6938–43.

397 Vergison A, Tuerlinckx D, Verhaegen J, Malfroot A, for the Belgian Invasive Pneumococcal Disease Study Group. Epidemiologic Features of Invasive Pneumococcal Disease in Belgian Children: Passive Surveillance Is Not Enough. *PEDIATRICS* 2006; **118**: e801–9.

398 Stratchounski LS, Kozlov RS, Appelbaum PC, Kretchikova OI, Kosowska-Shick K. Antimicrobial resistance of nasopharyngeal pneumococci from children from day-care centres and orphanages in Russia: results of a unique prospective multicentre study. *Clin Microbiol Infect* 2006; **12**: 853–66.

399 Lauderdale T-L, Wagener MM, Lin HM, *et al.* Serotype and antimicrobial resistance patterns of Streptococcus pneumoniae isolated from Taiwanese children: comparison of nasopharyngeal and clinical isolates. *Diagn Microbiol Infect Dis* 2006; **56**: 421–6.

400 Chen Y-Y, Yao S-M, Chou C-Y, *et al.* Surveillance of invasive Streptococcus pneumoniae in Taiwan, 2002-2003. *J Med Microbiol* 2006; **55**: 1109–14.

401 De Schutter I, Malfroot A, Piérard D, Lauwers S. Pneumococcal serogroups and serotypes in severe pneumococcal pneumonia in Belgian children: Theoretical coverage of the 7-valent and 9-valent pneumococcal conjugate vaccines. *Pediatr Pulmonol* 2006; **41**: 765–70.

402 Wexler ID, Knoll S, Picard E, *et al.* Clinical characteristics and outcome of complicated pneumococcal pneumonia in a pediatric population. *Pediatr Pulmonol* 2006; **41**: 726–34.

403 Lin W-J, Lo W-T, Chou C-Y, *et al.* Antimicrobial resistance patterns and serotype distribution of invasive Streptococcus pneumoniae isolates from children in Taiwan from 1999 to 2004. *Diagn Microbiol Infect Dis* 2006; **56**: 189–96.

404 Dias R, Louro D, the Antimicrobial Resistance Surveillance Program in Portugal, Canica M. Antimicrobial Susceptibility of Invasive Streptococcus pneumoniae Isolates in Portugal over an 11-Year Period. *Antimicrob Agents Chemother* 2006; **50**: 2098–105.

405 Žemličková H, URBÁšKOVÁ P, Adámková V, Motlová J, Lebedová V, Procházka B. Characteristics of Streptococcus pneumoniae, Haemophilus influenzae , Moraxella catarrhalis and Staphylococcus aureus isolated from the nasopharynx of healthy children attending day-care centres in the Czech Republic. *Epidemiol Infect* 2006; **134**: 1179–87.

406 Paraskakis I, Kafetzis DA, Chrisakis A, *et al.* Serotypes and antimicrobial susceptibilities of 1033 pneumococci isolated from children in Greece during 2001–2004. *Clin Microbiol Infect* 2006; **12**: 490–3.

407 Yalçın I, Gürler N, Alhan E, *et al.* Serotype distribution and antibiotic susceptibility of invasive Streptococcus pneumoniae disease isolates from children in Turkey, 2001–2004. *Eur J Pediatr* 2006; **165**: 654–7.

408 Kyaw MH, Lynfield R, Schaffner W, *et al.* Effect of Introduction of the Pneumococcal Conjugate Vaccine on Drug-Resistant Streptococcus pneumoniae. *N Engl J Med* 2006; **354**: 1455–63.

409 Cardozo DM, Nascimento-Carvalho CM, Brandão MA, *et al.* Antimicrobial Resistance and Serotypes of Nasopharyngeal Strains of Streptococcus pneumoniae in Brazilian Adolescents. *Microb Drug Resist* 2006; **12**: 29–32.

410 Quintero B, Araque M. Serotype profile and antibiotyping of Streptococcus pneumoniae strains isolated from nasal carriage in pediatric patients. *Invest Clin* 2006; **47**: 17–26.

411 Valles X, Flannery B, Roca A, *et al.* Serotype distribution and antibiotic susceptibility of invasive and nasopharyngeal isolates of Streptococcus pneumoniae among children in rural Mozambique. *Trop Med Int Health* 2006; **11**: 358–66.

412 Steenhoff AP, Shah SS, Ratner AJ, Patil SM, McGowan KL. Emergence of Vaccine-Related Pneumococcal Serotypes as a Cause of Bacteremia. *Clin Infect Dis* 2006; **42**: 907–14.

413 Laval CB, de Andrade ALSS, Pimenta FC, *et al.* Serotypes of carriage and invasive isolates of Streptococcus pneumoniae in Brazilian children in the era of pneumococcal vaccines. *Clin Microbiol Infect* 2006; **12**: 50–5.

414 Al Khorasani A, Banajeh S. Bacterial profile and clinical outcome of childhood meningitis in rural Yemen: A 2-year hospital-based study. *J Infect* 2006; **53**: 228–34.

415 Siedler A, Reinert RR, Toschke M, Al-Lahham A. Regional Differences in the Epidemiology of Invasive Pneumococcal Disease in Toddlers in Germany. *Pediatr Infect Dis J* 2005; **24**: 1114–5.

416 Demachy M-C, Vernet-Garnier V, Cottin J, *et al.* Antimicrobial Resistance Data on 16,756 Streptococcus pneumoniae Isolates in 1999: A Pan-Regional Multicenter Surveillance Study in France. *Microb Drug Resist* 2005; **11**: 323–9.

417 Mato R, Sanches IS, Simas C, *et al.* Natural History of Drug-Resistant Clones of Streptococcus pneumoniae Colonizing Healthy Children in Portugal. *Microb Drug Resist* 2005; **11**: 309–22.

418 Charvériat MA, Chomarat M, Watson M, Garin B. Study of the nasopharyngeal carriage of Streptococcus pneumoniae in healthy children aged 2 to 24 months in New Caledonia. *Médecine Mal Infect* 2005; **35**: 500–6.

419 Ogunlesi TA, Okeniyi J a. O, Oyelami OA. Pyogenic meningitis in Ilesa, Nigeria. *Indian Pediatr* 2005; **42**: 1019–23.

420 Lauderdale T-L, Lee WY, Cheng MF, *et al.* High carriage rate of high-level penicillin-resistant Streptococcus pneumoniaein a Taiwan kindergarten associated with a case of pneumococcal meningitis. *BMC Infect Dis* 2005; **5**: 96.

421 Solórzano-Santos F, Ortiz-Ocampo LA, Miranda-Novales MG, Echániz-Avilés G, Soto-Noguerón A, Guiscafré-Gallardo H. Prevalence of Streptococcus pneumoniae serotypes on nasopharyngeal colonization in children of Mexico City. *Salud Pública México* 2005; **47**: 276–81.

422 Babay HA, Twum-Danso K, Kambal AM, Al-Otaibi FE. Bloodstream infections in pediatric patients. *Saudi Med J* 2005; **26**: 1555–61.

423 Hennessy TW, Singleton RJ, Bulkow LR, *et al.* Impact of heptavalent pneumococcal conjugate vaccine on invasive disease, antimicrobial resistance and colonization in Alaska Natives: progress towards elimination of a health disparity. *Vaccine* 2005; **23**: 5464–73.

424 Hussain M, Melegaro A, Pebody RG, *et al.* A longitudinal household study of Streptococcus pneumoniae nasopharyngeal carriage in a UK setting. *Epidemiol Infect* 2005; **133**: 891–8.

425 Guillemot D, Varon E, Bernede C, *et al.* Reduction of Antibiotic Use in the Community Reduces the Rate of Colonization with Penicillin G--Nonsusceptible Streptococcus pneumoniae. *Clin Infect Dis* 2005; **41**: 930–8.

426 Huang SS, Platt R, Rifas-Shiman SL, Pelton SI, Goldmann D, Finkelstein JA. Post-PCV7 changes in colonizing pneumococcal serotypes in 16 Massachusetts communities, 2001 and 2004. *Pediatrics* 2005; **116**: e408-413.

427 Arri SJ, Fluegge K, Mueller U, Berner R. Antibiotic resistance patterns among respiratory pathogens at a german university children's hospital over a period of 10 years. *Eur J Pediatr* 2006; **165**: 9–13.

428 Bayraktar M, Durmaz B, Kalcioglu M, Durmaz R, Cizmeci Z, Aktas E. Nasopharyngeal carriage, antimicrobial susceptibility, serotype distribution and clonal relatedness of isolates in healthy children in Malatya, Turkey. *Int J Antimicrob Agents* 2005; **26**: 241–6.

429 Tomasson G, Gudnason T, Kristinsson KG. Dynamics of pneumococcal carriage among healthy Icelandic children attending day-care centres. *Scand J Infect Dis* 2005; **37**: 422–8.

430 McEllistrem MC, Adams JM, Shutt K, *et al.* Erythromycin-nonsusceptible Streptococcus pneumoniae in Children, 1999–2001. *Emerg Infect Dis* 2005; **11**: 969–72.

431 Byington CL, Samore MH, Stoddard GJ, *et al.* Temporal Trends of Invasive Disease Due to Streptococcus pneumoniae among Children in the Intermountain West: Emergence of Nonvaccine Serogroups. *Clin Infect Dis* 2005; **41**: 21–9.

432 Ochoa TJ, Rupa R, Guerra H, *et al.* Penicillin resistance and serotypes/serogroups of Streptococcus pneumoniae in nasopharyngeal carrier children younger than 2 years in Lima, Peru. *Diagn Microbiol Infect Dis* 2005; **52**: 59–64.

433 Wasfy MO, Pimentel G, Abdel-Maksoud M, *et al.* Antimicrobial susceptibility and serotype distribution of Streptococcus pneumoniae causing meningitis in Egypt, 1998–2003. *J Antimicrob Chemother* 2005; **55**: 958–64.

434 Frazão N, Brito-Avô A, Simas C, *et al.* Effect of the seven-valent conjugate pneumococcal vaccine on carriage and drug resistance of Streptococcus pneumoniae in healthy children attending day-care centers in Lisbon. *Pediatr Infect Dis J* 2005; **24**: 243–52.

435 Watson M, Roche P, Bayley K, *et al.* Laboratory surveillance of invasive pneumococcal disease in Australia, 2003 predicting the future impact of the universal childhood conjugate vaccine program. *Commun Dis Intell Q Rep* 2004; **28**: 455–64.

436 Jain A, Kumar P, Awasthi S. High nasopharyngeal carriage of drug resistant Streptococcus pneumoniae and Haemophilus influenzae in North Indian schoolchildren. *Trop Med Int Health* 2005; **10**: 234–9.

437 Grenon S, Von Specht M, Corso A, Pace J, Regueira M. Distribución de serotipos y perfiles de sensibilidad a los antimicrobianos de cepas de Streptococcus pneumoniae aisladas en niños en Misiones, Argentina. *Enfermedades Infecc Microbiol Clínica* 2005; **23**: 10–4.

438 Pai R, Moore MR, Pilishvili T, *et al.* Postvaccine genetic structure of Streptococcus pneumoniae serotype 19A from children in the United States. *J Infect Dis* 2005; **192**: 1988–95.

439 Haddy RI, Perry K, Chacko CE, *et al.* Comparison of Incidence of Invasive Streptococcus pneumoniae Disease Among Children Before and After Introduction of Conjugated Pneumococcal Vaccine. *Pediatr Infect Dis J* 2005; **24**: 320–3.

440 Nunes S, Sa-Leao R, Carrico J, *et al.* Trends in Drug Resistance, Serotypes, and Molecular Types of Streptococcus pneumoniae Colonizing Preschool-Age Children Attending Day Care Centers in Lisbon, Portugal: a Summary of 4 Years of Annual Surveillance. *J Clin Microbiol* 2005; **43**: 1285–93.

441 Gazi H, Kurutepe S, Sürücüoğlu S, Teker A, Ozbakkaloglŭ B. Antimicrobial susceptibility of bacterial pathogens in the oropharynx of healthy school children in Turkey. *Indian J Med Res* 2004; **120**: 489–94.

442 Oteo J, Lazaro E, de Abajo FJ, Baquero F, Campos J, Spanish Members of the European Antimicrobial Resistance Surveillance System. Trends in Antimicrobial Resistance in 1,968 Invasive Streptococcus pneumoniae Strains Isolated in Spanish Hospitals (2001 to 2003): Decreasing Penicillin Resistance in Children's Isolates. *J Clin Microbiol* 2004; **42**: 5571–7.

443 Ghaffar F, Barton T, Lozano J, *et al.* Effect of the 7-Valent Pneumococcal Conjugate Vaccine on Nasopharyngeal Colonization by Streptococcus pneumoniae in the First 2 Years of Life. *Clin Infect Dis* 2004; **39**: 930–8.

444 Sulikowska A, Grzesiowski P, Sadowy E, Fiett J, Hryniewicz W. Characteristics of Streptococcus pneumoniae, Haemophilus influenzae, and Moraxella catarrhalis Isolated from the Nasopharynges of Asymptomatic Children and Molecular Analysis of S. pneumoniae and H. influenzae Strain Replacement in the Nasopharynx. *J Clin Microbiol* 2004; **42**: 3942–9.

445 Malfroot A, Verhaegen J, Dubru J-M, Van Kerschaver E, Leyman S. A cross-sectional survey of the prevalence of Streptococcus pneumoniae nasopharyngeal carriage in Belgian infants attending day care centres. *Clin Microbiol Infect* 2004; **10**: 797–803.

446 Decousser J-W, Pina P, Viguier F, Picot F, Courvalin P, Allouch P. Invasive Streptococcus pneumoniae in France: Antimicrobial Resistance, Serotype, and Molecular Epidemiology Findings from a Monthly National Study in 2000 to 2002. *Antimicrob Agents Chemother* 2004; **48**: 3636–9.

447 Ho P-L, Que T-L, Chiu SS, *et al.* Fluoroquinolone and Other Antimicrobial Resistance in Invasive Pneumococci, Hong Kong, 1995–2001. *Emerg Infect Dis* 2004; **10**: 1250–7.

448 Ho PL, Lam KF, Chow FKH, *et al.* Serotype distribution and antimicrobial resistance patterns of nasopharyngeal and invasive Streptococcus pneumoniae isolates in Hong Kong children. *Vaccine* 2004; **22**: 3334–9.

449 Mufson MA, Stanek RJ. Epidemiology of Invaisve Streptococcus Pneumoniae Infections and Vaccine Implications Among Children in a Wester Virginia Community, 1978- 2003. *Pediatr Infect Dis J* 2004; **23**: 779–81.

450 Campbell JD, Kotloff KL, Sow SO, *et al.* Invasive Pneumococcal Infections Among Hospitalized Children in Bamako, Mali. *Pediatr Infect Dis J* 2004; **23**: 642–9.

451 Serrano I, Ramirez M, The Portuguese Surveillance Group for the Study of Respiratory Pathogens, Melo-Cristino J. Invasive Streptococcus pneumoniae from Portugal: implications for vaccination and antimicrobial therapy. *Clin Microbiol Infect* 2004; **10**: 652–6.

452 Rendi-Wagner P. Prospective surveillance of incidence, serotypes and antimicrobial susceptibility of invasive Streptococcus pneumoniae among hospitalized children in Austria. *J Antimicrob Chemother* 2004; **53**: 826–31.

453 Immergluck LC, Kanungo S, Schwartz A, McINTYRE A, Schreckenberger PC, Diaz PS. Prevalence of Streptococcus pneumoniae and Staphylococcus aureus nasopharyngeal colonization in healthy children in the United States. *Epidemiol Infect* 2004; **132**: 159–66.

454 McGregor D, Barton M, Thomas S, Christie CD. Invasive pneumococcal disease in Jamaican children. *Ann Trop Paediatr* 2004; **24**: 33–40.

455 Regev-Yochay G, Raz M, Dagan R, *et al.* Nasopharyngeal Carriage of Streptococcus pneumoniae by Adults and Children in Community and Family Settings. *Clin Infect Dis* 2004; **38**: 632–9.

456 Latorre C, Gené A, Juncosa T, Muñoz-Almagro C, González-Cuevas A. Characterisation of invasive pneumococcal isolates in Catalan children up to 5 years of age, 1989–2000. *Clin Microbiol Infect* 2004; **10**: 177–81.

457 Decousser J-W, Collignon A, Chaplain C, *et al.* Multicentre Study of the Molecular Epidemiology, Serotypes and Antimicrobial Susceptibility Patterns of Invasive Streptococcus pneumoniae Invasive Isolated from Children in the Ille de France Area. *Eur J Clin Microbiol Infect Dis* 2004; **23**: 27–33.

458 Watson M, Bayley K, Bell JM, *et al.* Laboratory surveillance of invasive pneumococcal disease in Australia in 2001 to 2002--implications for vaccine serotype coverage. *Commun Dis Intell Q Rep* 2003; **27**: 478–87.

459 Quagliarello AB, Parry CM, Hien TT, Farrar JJ. Factors associated with carriage of penicillin-resistant Streptococcus pneumoniae among Vietnamese children: a rural-urban divide. *J Health Popul Nutr* 2003; **21**: 316–24.

460 Henriques Normark B, Christensson B, Sandgren A, *et al.* Clonal Analysis of Streptococcus pneumoniae Nonsusceptible to Penicillin at Day-Care Centers with Index Cases, in a Region with Low Incidence of Resistance: Emergence of an Invasive Type 35B Clone among Carriers. *Microb Drug Resist* 2003; **9**: 337–44.

461 Ulloa-Gutierrez R, Avila-Aguero ML, Herrera ML, Herrera JF, Arguedas A. Invasive pneumococcal disease in Costa Rican children: a seven year survey: *Pediatr Infect Dis J* 2003; **22**: 1069–74.

462 Saha SK, Baqui AH, Darmstadt GL, *et al.* Comparison of Antibiotic Resistance and Serotype Composition of Carriage and Invasive Pneumococci among Bangladeshi Children: Implications for Treatment Policy and Vaccine Formulation. *J Clin Microbiol* 2003; **41**: 5582–7.

463 Greenberg D, Dagan R, Muallem M, Porat N. Antibiotic-Resistant Invasive Pediatric Streptococcus pneumoniae Clones in Israel. *J Clin Microbiol* 2003; **41**: 5541–5.

464 Pantosti A, Boccia D, D’Ambrosio F, Recchia S, Orefici G, Moro ML. Inferring the Potential Success of Pneumococcal Vaccination in Italy: Serotypes and Antibiotic Resistance of Streptococcus pneumoniae Isolates from Invasive Diseases. *Microb Drug Resist* 2003; **9**: 61–8.

465 Hoffman JA, Mason EO, Schutze GE, *et al.* Streptococcus pneumoniae Infections in the Neonate. *PEDIATRICS* 2003; **112**: 1095–102.

466 Sirinavin S, Vorachit M, Thakkinstian A, Hongsanguensri S, Wittayawongsruji P. Pediatric invasive pneumococcal disease in a teaching hospital in Bangkok. *Int J Infect Dis* 2003; **7**: 183–9.

467 Klugman KP, Madhi SA, Huebner RE, Kohberger R, Mbelle N, Pierce N. A Trial of a 9-Valent Pneumococcal Conjugate Vaccine in Children with and Those without HIV Infection. *N Engl J Med* 2003; **349**: 1341–8.

468 Hedlund J, Sörberg M, Normark BH, Kronvall G. Capsular Types and Antibiotic Susceptibility of Invasive Streptococcus pneumoniae Among Children in Sweden. *Scand J Infect Dis* 2003; **35**: 452–8.

469 Pallares R, Fenoll A, Liñares J. The epidemiology of antibiotic resistance in Streptococcus pneumoniae and the clinical relevance of resistance to cephalosporins, macrolides and quinolones. *Int J Antimicrob Agents* 2003; **22**: 15–24.

470 Batt SL, Charalambous BM, Solomon AW, *et al.* Impact of Azithromycin Administration for Trachoma Control on the Carriage of Antibiotic-Resistant Streptococcus pneumoniae. *Antimicrob Agents Chemother* 2003; **47**: 2765–9.

471 Lee C-Y, Chiu C-H, Huang Y-C, *et al.* Invasive pneumococcal infections: a clinical and microbiological analysis of 53 patients in Taiwan. *Clin Microbiol Infect* 2003; **9**: 614–8.

472 Zhao G-M, Black S, Shinefield H, *et al.* Serotype distribution and antimicrobial resistance patterns in Streptococcus pneumoniae isolates from hospitalized pediatric patients with respiratory infections in Shanghai, China. *Pediatr Infect Dis J* 2003; **22**: 739–42.

473 Waterer GW, Buckingham SC, Kessler LA, Quasney MW, Wunderink RG. Decreasing β-Lactam Resistance in Pneumococci From the Memphis Region*. *Chest* 2003; **124**: 519–25.

474 Feikin DR, Davis M, Nwanyanwu OC, *et al.* Antibiotic resistance and serotype distribution of Streptococcus pneumoniae colonizing rural Malawian children. *Pediatr Infect Dis J* 2003; **22**: 564–7.

475 Chen J-Y, Fung CP, Wang CC, Chu ML, Siu LK. Susceptibility of Six Fluoroquinolones against Invasive Streptococcus pneumoniae Isolated from 1996 to 2001 in Taiwan. *Microb Drug Resist* 2003; **9**: 211–7.

476 Dagan R, Givon-Lavi N, Zamir O, Fraser D. Effect of a nonavalent conjugate vaccine on carriage of antibiotic-resistant Streptococcus pneumoniae in day-care centers: *Pediatr Infect Dis J* 2003; **22**: 532–9.

477 Givon-Lavi N, Fraser D, Dagan R. Vaccination of day-care center attendees reduces carriage of Streptococcus pneumoniae among their younger siblings. *Pediatr Infect Dis J* 2003; **22**: 524–31.

478 Whitney CG, Farley MM, Hadler J, *et al.* Decline in Invasive Pneumococcal Disease after the Introduction of Protein–Polysaccharide Conjugate Vaccine. *N Engl J Med* 2003; **348**: 1737–46.

479 Hjaltested EKR, Bernatoniene J, Erlendsdottir H, *et al.* Resistance in Respiratory Tract Pathogens and Antimicrobial Use in Icelandic and Lithuanian Children. *Scand J Infect Dis* 2003; **35**: 21–6.

480 Berezin EN, Falleiros-Carvalho LH, Lopes CR, *et al.* Pneumococcal meningitis in children: clinical findings, most frequent serotypes and outcome. *J Pediatr (Rio J)* 2002; **78**: 19–23.

481 Rey LC, Wolf B, Moreira JLB, Verhoef J, Farhat CK. Nasopharyngeal isolates of S. pneumoniae from healthy carriers and children with pneumonia: colonization rates and antimicrobial susceptibility. *J Pediatr (Rio J)* 2002; **78**: 105–12.

482 Denno DM, Frimpong E, Gregory M, Steele RW. Nasopharyngeal carriage and susceptibility patterns of Streptococcus pneumoniae in Kumasi, Ghana. *West Afr J Med* 2002; **21**: 233–6.

483 Coles CL, Rahmathullah L, Kanungo R, *et al.* Nasopharyngeal carriage of resistant pneumococci in young South Indian infants. *Epidemiol Infect* 2002; **129**: 491–7.

484 Hills SL, Hanna JN, Murphy D. Invasive pneumococcal disease in north Queensland, 2001. *Commun Dis Intell Q Rep* 2002; **26**: 520–4.

485 Roche P, Krause V, Enhanced Pneumococcal Surveillance Group of the Pneumococcal Working Party of the Communicable Diseases Network Australia. Invasive pneumococcal disease in Australia, 2001. *Commun Dis Intell Q Rep* 2002; **26**: 505–19.

486 Lagos R, Muñoz A, Valenzuela MT, Heitmann I, Levine MM. Population-based surveillance for hospitalized and ambulatory pediatric invasive pneumococcal disease in Santiago, Chile: *Pediatr Infect Dis J* 2002; **21**: 1115–23.

487 Mwangi I, Berkley J, Lowe B, Peshu N, Marsh K, Newton CRJC. Acute bacterial meningitis in children admitted to a rural Kenyan hospital: increasing antibiotic resistance and outcome: *Pediatr Infect Dis J* 2002; **21**: 1042–8.

488 Cullotta AR, Kalter HD, Delgado J, *et al.* Antimicrobial Susceptibilities and Serotype Distribution of Streptococcus pneumoniae Isolates from a Low Socioeconomic Area in Lima, Peru. *Clin Vaccine Immunol* 2002; **9**: 1328–31.

489 Syrogiannopoulos GA, Katopodis GD, Grivea IN, Beratis NG. Antimicrobial Use and Serotype Distribution of Nasopharyngeal Streptococcus pneumoniae Isolates Recovered from Greek Children Younger than 2 Years Old. *Clin Infect Dis* 2002; **35**: 1174–82.

490 Kellner JD, Scheifele DW, Halperin SA, *et al.* Outcome of penicillin-nonsusceptible Streptococcus pneumoniae meningitis: a nested case-control study. *Pediatr Infect Dis J* 2002; **21**: 903–9.

491 Siu LK, Chu M-L, Ho M, Lee Y-S, Wang C-C. Epidemiology of Invasive Pneumococcal Infection in Taiwan: Antibiotic Resistance, Serogroup Distribution, and Ribotypes Analyses. *Microb Drug Resist* 2002; **8**: 201–8.

492 Arason VA, Gunnlaugsson A, Sigurdsson JA, Erlendsdottir H, Gudmundsson S, Kristinsson KG. Clonal Spread of Resistant Pneumococci Despite Diminished Antimicrobial Use. *Microb Drug Resist* 2002; **8**: 187–92.

493 Christie D, Coleman D, Wan X, Jacobs M, Carapetis J. Childhood invasive pneumococcal disease in Tasmania, 1994-2000. *J Paediatr Child Health* 2002; **38**: 445–9.

494 Quach C, Weiss K, Moore D, Rubin E, McGeer A, Low DE. Clinical aspects and cost of invasive Streptococcus pneumoniae infections in children: resistant vs. susceptible strains. *Int J Antimicrob Agents* 2002; **20**: 113–8.

495 Rey LC, Wolf B, Moreira JLB, Milatovic D, Verhoef J, Farhat CK. Antimicrobial susceptibility and serotypes of nasopharyngeal Streptococcus pneumoniae in children with pneumonia and in children attending day-care centres in Fortaleza, Brazil. *Int J Antimicrob Agents* 2002; **20**: 86–92.

496 McMaster P. The emergence of resistant pneumococcal meningitis--implications for empiric therapy. *Arch Dis Child* 2002; **87**: 207–10.

497 Herruzo R, Chamorro L, García ME, *et al.* Prevalence and antimicrobial-resistance of S. pneumoniae and S. pyogenes in healthy children in the region of Madrid. *Int J Pediatr Otorhinolaryngol* 2002; **65**: 117–23.

498 Lonks JR, Garau J, Gomez L, *et al.* Failure of Macrolide Antibiotic Treatment in Patients with Bacteremia Due to Erythromycin-Resistant Streptococcus pneumoniae. *Clin Infect Dis* 2002; **35**: 556–64.

499 Masuda K, Masuda R, Nishi J-I, Tokuda K, Yoshinaga M, Miyata K. Incidences of nasopharyngeal colonization of respiratory bacterial pathogens in Japanese children attending day-care centers. *Pediatr Int* 2002; **44**: 376–80.

500 Flamaing J. Streptococcus pneumoniae bacteraemia in Belgium: differential characteristics in children and the elderly population and implications for vaccine use. *J Antimicrob Chemother* 2002; **50**: 43–50.

501 Bakir M, Yagci A, Akbenlioglu C, Ilki A, Ulger N, Soyletir G. Epidemiology of Streptococcus pneumoniae pharyngeal carriage among healthy Turkish infants and children. *Eur J Pediatr* 2002; **161**: 165–6.

502 Hennessy TW, Bruden D, Petersen KM, *et al.* Effect of high-dose amoxicillin on the prevalence of penicillin-resistant Streptococcus pneumoniae in rural Alaska. *JAMA* 2002; **287**: 2078–9.

503 Contreras L, Fica A, Figueroa O, Enríquez N, Urrutia P, Herrera P. Resistance to Streptococcus pneumoniae to penicillin and its association with clinical and epidemiological factors. *Rev Med Chil* 2002; **130**: 26–34.

504 Petrosillo N, Pantosti A, Bordi E, *et al.* Prevalence, Determinants, and Molecular Epidemiology of Streptococcus pneumoniae Isolates Colonizing the Nasopharynx of Healthy Children in Rome. *Eur J Clin Microbiol Infect Dis* 2002; **21**: 181–8.

505 Ma J-S, Chen P-Y, Mak S-C, Chi C-S, Lau Y-J. Clinical outcome of invasive pneumococcal infection in children: a 10-year retrospective analysis. *J Microbiol Immunol Infect Wei Mian Yu Gan Ran Za Zhi* 2002; **35**: 23–8.

506 Gómez-Barreto D, Calderón-Jaimes E, S Rodríguez R, Espinosa LE, Viña-Flores L, Jiménez-Rojas V. Carriage of antibiotic-resistant pneumococci in a cohort of a daycare center. *Salud Pública México* 2002; **44**. DOI:10.1590/S0036-36342002000100004.

507 Cizman M, Pokorn M, Paragi M. Antimicrobial resistance of invasive Streptococcus pneumoniae in Slovenia from 1997 to 2000. *J Antimicrob Chemother* 2002; **49**: 582–4.

508 Nasrin D, Collignon PJ, Roberts L, Wilson EJ, Pilotto LS, Douglas RM. Effect of beta lactam antibiotic use in children on pneumococcal resistance to penicillin: prospective cohort study. *BMJ* 2002; **324**: 28–28.

509 Greenberg D, Speert DP, Mahenthiralingam E, *et al.* Emergence of Penicillin-Nonsusceptible Streptococcus pneumoniae Invasive Clones in Canada. *J Clin Microbiol* 2002; **40**: 68–74.

510 Albanese BA, Roche JC, Margaret P, *et al.* Geographic, Demographic, and Seasonal Differences in Penicillin-Resistant Streptococcus pneumoniae in Baltimore. *Clin Infect Dis* 2002; **34**: 15–21.

511 Scheifele D, Halperin S, Pelletier L, *et al.* Reduced Susceptibility to Penicillin among Pneumococci Causing Invasive Infection in Children - Canada, 1991-1998. *Can J Infect Dis* 2001; **12**: 241–6.

512 Rossi F, Andreazzi D, Maffucci M, Pereira AA. Susceptibility of S. pneumoniae to various antibiotics among strains isolated from patients and healthy carriers in different regions of Brazil (1999-2000). *Braz J Infect Dis* 2001; **5**. DOI:10.1590/S1413-86702001000600003.

513 Hortal M, Lovgren M, de la Hoz F, *et al.* Antibiotic Resistance in Streptococcus pneumoniae in Six Latin American Countries: 1993-1999 Surveillance. *Microb Drug Resist* 2001; **7**: 391–401.

514 Marchisio P, Gironi S, Esposito S, Schito GC, Mannelli S, Principi N. Seasonal variations in nasopharyngeal carriage of respiratory pathogens in healthy Italian children attending day-care centres or schools. *J Med Microbiol* 2001; **50**: 1095–9.

515 Buckingham SC, Mccullers JA, Luján-Zilbermann J, Knapp KM, Orman KL, English BK. Pneumococcal meningitis in children: relationship of antibiotic resistance to clinical characteristics and outcomes: *Pediatr Infect Dis J* 2001; **20**: 837–43.

516 Di Fabio JL, Castañeda E, Agudelo CI, *et al.* Evolution of S treptococcus pneumoniae serotypes and penicillin susceptibility in Latin America, Sireva-Vigía Group, 1993 to 1999. *Pediatr Infect Dis J* 2001; **20**: 959–67.

517 Stovall SH, Ainley KA, Mason EO, *et al.* Invasive pneumococcal infections in pediatric cardiac transplant patients: *Pediatr Infect Dis J* 2001; **20**: 946–50.

518 Boost MV, O'Donoghue MM, Dooley JS. Prevalence of carriage of antimicrobial resistant strains of Streptococcus pneumoniae in primary school children in Hong Kong. *Epidemiol Infect* 2001; **127**. DOI:10.1017/S0950268801005647.

519 Chiu SS, Ho PL, Chow FKH, Yuen KY, Lau YL. Nasopharyngeal Carriage of Antimicrobial-Resistant Streptococcus pneumoniae among Young Children Attending 79 Kindergartens and Day Care Centers in Hong Kong. *Antimicrob Agents Chemother* 2001; **45**: 2765–70.

520 Kacou-N'douba A, Bouzid SA, Guessennd KN, Kouassi-M'bengue AA, Faye-Kette AYH, Dosso M. Antimicrobial resistance of nasopharyngeal isolates of Streptococcus pneumonia in healthy carriers: report of a study in 5-year-olds in Marcory, Abidjan, Côte d'Ivoire. *Ann Trop Paediatr* 2001; **21**: 149–54.

521 Fraser D, Givon-Lavi N, Bilenko N, Dagan R. A Decade (1989–1998) of Pediatric Invasive Pneumococcal Disease in 2 Populations Residing in 1 Geographic Location: Implications for Vaccine Choice. *Clin Infect Dis* 2001; **33**: 421–7.

522 Doern GV, Heilmann KP, Huynh HK, Rhomberg PR, Coffman SL, Brueggemann AB. Antimicrobial Resistance among Clinical Isolates of Streptococcus pneumoniae in the United States during 1999-2000, Including a Comparison of Resistance Rates since 1994-1995. *Antimicrob Agents Chemother* 2001; **45**: 1721–9.

523 Joloba ML, Bajaksouzian S, Palavecino E, Whalen C, Jacobs MR. High prevalence of carriage of antibiotic-resistant Streptococcus pneumoniae in children in Kampala Uganda. *Int J Antimicrob Agents* 2001; **17**: 395–400.

524 Kaplan SL, Mason EO, Barson WJ, *et al.* Outcome of invasive infections outside the central nervous system caused by Streptococcus pneumoniae isolates nonsusceptible to ceftriaxone in children treated with beta-lactam antibiotics. *Pediatr Infect Dis J* 2001; **20**: 392–6.

525 Lee NY, Song J-H, Kim S, *et al.* Carriage of Antibiotic-Resistant Pneumococci among Asian Children: A Multinational Surveillance by the Asian Network for Surveillance of Resistant Pathogens (ANSORP). *Clin Infect Dis* 2001; **32**: 1463–9.

526 Pírez MC, Martínez O, Ferrari AM, *et al.* Standard case management of pneumonia in hospitalized children in Uruguay, 1997 to 1998. *Pediatr Infect Dis J* 2001; **20**: 283–9.

527 Nascimento-Carvalho CMC, Lopes AA, Gomes MDBS, *et al.* Community acquired pneumonia among pediatric outpatients in Salvador, Northeast Brazil, with emphasis on the role of pneumococcus. *Braz J Infect Dis* 2001; **5**: 13–20.

528 Markovska R, Strateva T, Gergova G, Keuleyan E. Nasopharyngeal carriage of penicillin-resistant, macrolide-resistant and multiply-resistant Streptococcus pneumoniae in day-care centers in Sofia, Bulgaria. *Clin Microbiol Infect Off Publ Eur Soc Clin Microbiol Infect Dis* 2001; **7**: 42–6.

529 Soewignjo S, Gessner BD, Sutanto A, *et al.* Streptococcus pneumoniae Nasopharyngeal Carriage Prevalence, Serotype Distribution, and Resistance Patterns among Children on Lombok Island, Indonesia. *Clin Infect Dis* 2001; **32**: 1039–43.

530 Ip M, Lyon DJ, Cheng AFB. Patterns of Antibiotic Resistance, Serotype Distribution, and Patient Demographics of Streptococcus pneumoniae in Hong Kong. *Chemotherapy* 2001; **47**: 110–6.

531 Porat N, Trefler R, Dagan R. Persistence of two invasive Streptococcus pneumoniae clones of serotypes 1 and 5 in comparison to that of multiple clones of serotypes 6B and 23F among children in southern Israel. *J Clin Microbiol* 2001; **39**: 1827–32.

532 Naaher P, Tamm E, Pütsepp A, Kõljalg S, Maimets M. Nasopharyngeal carriage and antibacterial susceptibility of Streptococcus pneumoniae, Haemophilus influenzae and Moraxella catarrhalis in Estonian children. *Clin Microbiol Infect* 2000; **6**: 675–7.

533 Huebner RE, Wasas AD, Klugman KE. Trends in antimicrobial resistance and serotype distribution of blood and cerebrospinal fluid isolates of streptococcus pneumoniae in South Africa, 1991–1998. *Int J Infect Dis* 2000; **4**: 214–8.

534 Huebner RE, Wasas AD, Klugman KP, Paediatric Study Group. Prevalence of nasopharyngeal antibiotic-resistant pneumococcal carriage in children attending private paediatric practices in Johannesburg. *South Afr Med J Suid-Afr Tydskr Vir Geneeskd* 2000; **90**: 1116–21.

535 Fenoll A, Jado I, Vicioso D, Berrón S, Yuste J, Casal J. Streptococcus pneumoniae in children in Spain: 1990-1999. *Acta Paediatr* 2007; **89**: 44–50.

536 Kaltoft M, Zeuthen N, Konradsen H. Epidemiology of invasive pneumococcal infections in children aged 0-6 years in Denmark: a 19-year nationwide surveillance study. *Acta Paediatr* 2007; **89**: 3–10.

537 Miller E, Waight P, Efstratiou A, Brisson M, Johnson A, George R. Epidemiology of invasive and other pneumococcal disease in children in England and Wales 1996-1998. *Acta Paediatr* 2007; **89**: 11–6.

538 Melander E, Md KE, J??Nsson G, M??Lstad S. Frequency of penicillin-resistant pneumococci in children is correlated to community utilization of antibiotics: *Pediatr Infect Dis J* 2000; **19**: 1172–7.

539 Whitney CG, Farley MM, Hadler J, *et al.* Increasing Prevalence of Multidrug-Resistant Streptococcus pneumoniae in the United States. *N Engl J Med* 2000; **343**: 1917–24.

540 Perrone C, Perrone P, Kopetz V, Nedunchezian D, Leggiadro R. Prevalence of penicillin-nonsusceptible pneumococcal bacteremia in a Staten Island community hospital. *South Med J* 2000; **93**: 1078–80.

541 Westwood ATR, Eley BS, Gilbert RD, Hanslo D. Bacterial infection in children with HIV: a prospective study from Cape Town, South Africa. *Ann Trop Paediatr* 2000; **20**: 193–8.

542 McIntyre PB, Gilmour RE, Gilbert GL, Kakakios AM, Mellis CM. Epidemiology of invasive pneumococcal disease in urban New South Wales, 1997–1999. *Med J Aust* 2000; **173**. DOI:10.5694/j.1326-5377.2000.tb139409.x.

543 Wolf B. Molecular epidemiology of penicillin-resistant Streptococcus pneumoniae colonizing children with community-acquired pneumonia and children attending day-care centres in Fortaleza, Brazil. *J Antimicrob Chemother* 2000; **46**: 757–65.

544 Syrogiannopoulos GA, Grivea IN, Davies TA, Katopodis GD, Appelbaum PC, Beratis NG. Antimicrobial Use and Colonization with Erythromycin-Resistant Streptococcus pneumoniae in Greece during the First 2 Years of Life. *Clin Infect Dis* 2000; **31**: 887–93.

545 Lupisan SP, Herva E, Sombrero LT, *et al.* Invasive bacterial infections of children in a rural province in the central Philippines. *Am J Trop Med Hyg* 2000; **62**: 341–6.

546 Marco F, Bouza E, Garcia-de-Lomas J, Aguilar L. Streptococcus pneumoniae in community-acquired respiratory tract infections in Spain: the impact of serotype and geographical, seasonal and clinical factors on its susceptibility to the most commonly prescribed antibiotics. *J Antimicrob Chemother* 2000; **46**: 557–64.

547 von Kries R, Siedler A, Schmitt HJ, Reinert RR. Proportion of Invasive Pneumococcal Infections in German Children Preventable by Pneumococcal Conjugate Vaccines. *Clin Infect Dis* 2000; **31**: 482–7.

548 Rowland KE, Turnidge JD. The impact of penicillin resistance on the outcome of invasive Streptococcus pneumoniae infection in children. *Aust N Z J Med* 2000; **30**: 441–9.

549 Sá-Leão R, Tomasz A, Sanches IS, *et al.* Carriage of Internationally Spread Clones of Streptococcus pneumoniae with Unusual Drug Resistance Patterns in Children Attending Day Care Centers in Lisbon, Portugal. *J Infect Dis* 2000; **182**: 1153–60.

550 Polack FP, Flayhart DC, Zahurak ML, Dick JD, Willoughby RE. Colonization by Streptococcus pneumoniae in human immunodeficiency virus-infected children: *Pediatr Infect Dis J* 2000; **19**: 608–12.

551 Scheifele D, Halperin S, Pelletier L, Talbot J, Members of the Canadian Paediatric Society/Laboratory Centre for Disease Control Immunization Monitoring Program, Active (IMPACT). Invasive Pneumococcal Infections in Canadian Children, 1991-1998: Implications for New Vaccination Strategies. *Clin Infect Dis* 2000; **31**: 58–64.

552 Borres MP, Alestig K, Krantz I, Larsson P, Norvenius G, Stenqvist K. Carriage of penicillin-susceptible and non-susceptible pneumococci in healthy young children in Göteborg, Sweden. *J Infect* 2000; **40**: 141–4.

553 Raymond J, Le Thomas I, Moulin F, Commeau A, Gendrel D, Berche P. Sequential Colonization by Streptococcus pneumoniae of Healthy Children Living in an Orphanage. *J Infect Dis* 2000; **181**: 1983–8.

554 Syrogiannopoulos GA, Grivea IN, Katopodis GD, Geslin P, Jacobs MR, Beratis NG. Carriage of Antibiotic-Resistant Streptococcus pneumoniae in Greek Infants and Toddlers. *Eur J Clin Microbiol Infect Dis* 2000; **19**: 288–93.

555 Rowe AK, Deming MS, Schwartz B, *et al.* Antimicrobial resistance of nasopharyngeal isolates of Streptococcus pneumoniae and Haemophilus influenzae from children in the Central African Republic: *Pediatr Infect Dis J* 2000; **19**: 438–44.

556 Walsh AL, Phiri AJ, Graham SM, Molyneux EM, Molyneux ME. Bacteremia in febrile Malawian children: clinical and microbiologic features: *Pediatr Infect Dis J* 2000; **19**: 312–8.

557 Stratchounski LS, Kretchikova OI, Kozlov RS, *et al.* Antimicrobial resistance of Streptococcus pneumoniae isolated from healthy children in day-care centers: results of a multicenter study in Russia: *Pediatr Infect Dis J* 2000; **19**: 196–200.

558 Parry CM, Diep TS, Wain J, *et al.* Nasal Carriage in Vietnamese Children of Streptococcus pneumoniae Resistant to Multiple Antimicrobial Agents. *Antimicrob Agents Chemother* 2000; **44**: 484–8.
